# Current Knowledge of the Genus *Satureja*: A Comprehensive Review of Its Traditional Use, Phytochemistry, Pharmacological Activity and Non-Medical Applications

**DOI:** 10.3390/ph19060875

**Published:** 2026-05-31

**Authors:** Marah Alburqan, Katalin Veres, Judit Hohmann

**Affiliations:** 1 Institute of Pharmacognosy, University of Szeged, Eötvös Str. 6, 6720 Szeged, Hungary; 2 HUN-REN–USZ Biologically Active Natural Products Research Group, University of Szeged, Eötvös Str. 6, 6720 Szeged, Hungary

**Keywords:** *Satureja*, Lamiaceae family, essential oil, secondary metabolites, biological activity

## Abstract

**Background:** The genus *Satureja* L. (savory) includes approximately 200 aromatic herb and shrub species distributed worldwide. These plants are widely used in traditional and modern medicine, culinary practices, and agriculture. This review summarises knowledge on the traditional uses, phytochemistry, and pharmacological activities of *Satureja* species published between March 2014 and 2025. **Methods:** Peer-reviewed literature was searched on Web of Knowledge, PubMed, Scopus, and SciFinder using the keywords “*Satureja*” and “savory.” A total of 171 relevant articles were analyzed, focusing on ethnomedicinal use, chemical constituents, and pharmacological effects. **Results:** Recent ethnobotanical studies documented the use of local medicinal plants, including *Satureja*, in several European regions. Phytochemical research identified major groups of compounds such as essential oils, flavonoids, phenolic acids, jasmonates, di- and triterpenes, and steroids. Essential oils are the most studied and show high variability among species due to environmental and genetic factors. Pharmacological research largely highlights antimicrobial, antioxidant, and antitumor activities, as well as protective effects against chemotherapy-induced side effects. Additional studies report neurological benefits, including prevention of opioid analgesic tolerance, antiepileptic activity, and memory-enhancing effects. *Satureja* species have been the subject of various innovative developments aimed at preserving food quality, improving coating materials in the food industry, and developing new environmentally friendly biopesticides. **Conclusions:** Future research should prioritize the study of individual bioactive compounds, their mechanisms of action, and structure–activity relationships. Advances in nanoformulations and modern extraction technologies offer promising directions to support the medicinal and food-industry applications of *Satureja*-derived products.

## 1. Introduction

The genus *Satureja* L. (savory) is a member of the subfamily Nepetoideae and the tribe Mentheae within the family Lamiaceae. This genus includes approximately 200 wild species of aromatic herbs and shrubs that are widely distributed across Mediterranean regions, ranging from Southern Europe to North Africa and West Asia, the Canary Islands, and South America [1]. The name *Satureja* L. derives from the Latin *satureia* and was first named by the Roman writer Pliny [2]. The name, which translates to ‘herb of satyrs’, led to it being banned in monasteries [3].

The diverse uses of these plants extend to traditional and modern medicine, culinary arts, and agricultural practices. Traditionally, *Satureja* species have been used to treat multiple diseases, predominantly those with symptoms related to gastrointestinal and inflammatory disorders. The main indications include the relief of muscle pain and respiratory diseases and as antispasmodic, carminative, emmenagogic, and tonic agents, as well as the treatment of digestive problems, such as nausea, cramps, indigestion, and diarrhea [4,5,6].

The chemical composition of *Satureja* species is characterised by the presence of diverse and complex chemical constituents. Interestingly, essential oils (EOs), diterpenoids, steroids, flavonoids, caffeic acid polymers, and other phenolic compounds have emerged as vital constituents that significantly affect the biological activity of the genus [5]. Polyphenols, flavonoids, and volatile oils are responsible for the significant antioxidant, antimicrobial, antiparasitic, and anti-inflammatory properties of plant extracts. Most *Satureja* species are characterized by a high essential oil content (>1%) with thymol, carvacrol, γ-terpinene and cymene as main constituents; these compounds show evidence of antibacterial and antifungal activities against plant, food, and human pathogens [7,8]. Based on the presence of tannins, phenolic acids and flavonoids, pharmacological characteristics, such as antioxidant, analgesic, anti-inflammatory, and antihypercholesterolemic activity, have also been reported [9,10]. Furthermore, ongoing studies explore the cytotoxic effects of *Satureja* extracts and compounds on cancer cells, and some compounds have shown promise in cancer treatment [11]. Moreover, some species have shown potential in the management of diabetes, and compounds with antidiabetic activity have been identified [12].

Among *Satureja* species, *S. montana* L. (winter savory) and *S. hortensis* L. (summer savory) have been widely used due to their therapeutic value and distinctive aromatic profiles [13]. The preference for a particular species is closely related to local availability, cultural customs, and special culinary or health goals. Owing to their aromatic taste and simple cultivation, *Satureja* species are widely used in food flavorings, cosmetics, and pharmaceutical products.

**Figure 1:**
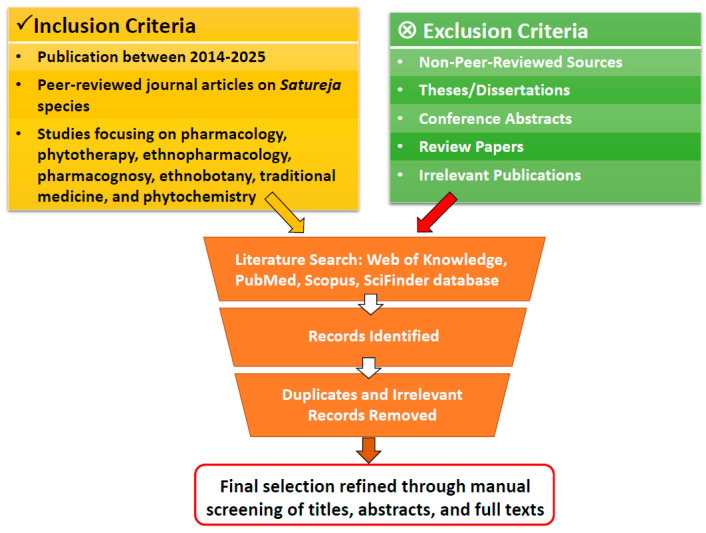
Overview of the article selection process used in this review of the literature.

In this review, comprehensive information on the traditional uses, phytochemistry, and pharmacological activities of *Satureja* species reported from March 2014 to 2025 is summarized (Figure 1). For this purpose, the databases Web of Knowledge, PubMed, Scopus, and SciFinder were searched, with a focus on ethnomedicinal use, chemical constituents, and pharmacological activities of the genus. The search terms were ‘*Satureja*’ or ‘Savory’. As a result, 171 peer-reviewed articles were found, which served as the basis for this review. Previous reviews have summarized only data on summer savory (*S. hortensis*) [14,15] or mainly focused on the constituents of EO [16], or the pharmacological and clinical aspects of *Satureja* genus [13].

## 2. Ethnobotanical Uses

During the study period, four publications addressed ethnobotanical knowledge related to *Satureja* species (Table 1). The ethnobotanical study of Tsioutsiou et al. investigated the use of local medicinal plants in the Macedonia region (North of Greece). Traditional use of the endemic species *S. montana* subsp. *macedonica* (Formánek) Baden was explored through extensive and semi-structured interviews. They found that flower infusion was applied to treat the flu and cough and to alleviate inflammation of the respiratory tract, and a decoction was used to treat hypercholesterolemia.

One particularly interesting finding was that *S. montana* subsp. *macedonica* was used to treat tinnitus and improve hearing, which is particularly unusual and has never been previously reported [17].

The importance of *S. montana* (winter savory) in the ethnobotany of the Trentino–South Tyrol region (Northeastern Italy) was studied by Cavalloro et al. [18]. This species was among the most cited and was primarily used to treat the gastrointestinal system. The local community used a winter savory tea to treat nervous gastric pains, bloating and vomiting [18].

An ethnobotanical study conducted in Sirjan district, Kerman Province, Iran, reported that the *S. bachtiarica* leaves were traditionally applied to treat flatulence in the form of an infusion. The authors called for more stringent government control over the preservation of *S. bachtiarica*, pointing out that the unrestricted harvesting of the species by locals has increased the risk of its extinction [19].

Matejic et al. studied the traditional medicine of the mountainous Svrljig region in eastern and southeast Serbia. They found that *S. montana* is one of the most used plants and the most characteristic species in the Svrljig region [20]. The tea prepared from winter savory is commonly used to treat respiratory diseases (productive cough, bronchitis), general and unspecified chills, and digestive problems and loss of appetite. *S. hortensis* was also mentioned in this study and the use of its aerial parts and leaves against digestive problems, loss of appetite, and stomach pain was described.

The indigenous knowledge mentioned above can contribute to the development of evidence-based complementary and alternative medicine in the future and is culturally significant.

## 3. Chemical Composition of *Satureja* Species

### 3.1. Essential Oil

Between 2014 and 2025, 55 publications investigated the chemical composition of EOs from *Satureja* species. Compared to other groups of compounds in the genus, EOs represent the most studied group. Below, we present the *Satureja* species producing EO in alphabetical order and summarize the reported information (Table 1).

*S. avromanica* Maroofi is a plant native to Iran that is frequently applied as a spice in the Avraman-Kurdistan region. Its EO content and the chemical profile of the EO were first studied by Abdali et al. The main EO constituents were *n*-alkans (34.8%) and sesquiterpene hydrocarbons (29.3%). The compounds present in a high percentage were *n*-pentacosane (*n*-C_25_H_52_, 23.8%), spathulenol (**1**) (11.5%), *β*-bourbonene (**2**) (11.3%), *n*-docosane (*n*-C_22_H_46_, 11.0%), bornyl acetate (**3**) (5.4%) and *β-*caryophyllene (**4**) (4.1%) [21].

*S. bachtiarica* Bunge is a subshrub endemic to Iran that grows primarily in temperate biomes. Alizadeh studied the EO constituents of the dried aerial parts of three wild-life *S. bachtiarica* ecotypes in southwestern Iran. The EO yield varied between 1.85% and 2.34% and carvacrol (**5**) (54.95% to 65.48%), thymol (**7**) (12.0% to 15.70%) and γ-terpinene (**8**) (4.55% to 13.55%) were found to be the main components. Based on these results, the ecotypes of *S. bachtiarica* were classified into four different chemotypes [22]. Jafari et al. identified carvacrol (**5**) (42.51%) and thymol (**7**) (38.75%) as the main compounds in *S. bachtiarica* EO, although the ratio of the compounds varied [23]. Memarzadeh et al. examined how two extraction methods, conventional hydrodistillation (HD) and microwave-assisted steam hydrodiffusion (MSHD), affected the yield and quality of the *S. bachtiarica* EO. They found that the HD approach yielded the highest concentrations of oxygenated monoterpenes and active phenolic compounds after 150 min, while the MSHD technique achieved this after 20 min. Both extraction methods produced similar EO yield and composition, with *γ*-terpinene (**8**), carvacrol (**5**), *p*-cymene (**6**) and thymol (**7**) as the major components. Compared with the standard HD method, the MSHD methodology reduced water usage, energy consumption, and extraction time [24].

*S. calamintha* (L.) Scheele [syn. *S. nepeta* (L.) Scheele] is a highly variable perennial herb with a European and North African distribution. This plant was extensively wild-harvested in Morocco and the size of its wild population has been significantly reduced; therefore, its cultivation is now required. The effect of domestication on the chemical profile and bioactivity of Moroccan *S. calamintha* EO was evaluated by Abbad et al. The results showed that cultivation had no notable effect on the production of the EO between wild and cultivated plants. The EO compositions were highly similar, with only minor differences in the relative abundances of the main EO compounds, including pulegone (**9**) (68.58%/72.93%), menthone (**11**) and menthol (**14**) [25]. In the study by Baghouz et al., the EO of *S. calamintha* from Morocco, extracted from the aerial parts (yield 1.40%), was found to contain pulegone (**9**) (21.48%), piperitenone oxide (**12**) (17.71%) and eucalyptol (**13**) (11.99%) as the main compounds [26]. A similar EO composition was observed when an Algerian sample of *S. calamintha* subsp. *nepeta* was analyzed [27]. In contrast to these findings, the analysis of the EO extracted from wild and domesticated *S. calamintha* of Algerian origin resulted in EO yields of 2.80% and 1.95%, respectively. Furthermore, 1,8-cineol (**13**) (23.10%), pulegone (**9**) (12.44%) and rotundifolone (**15**) (9.68%) were the predominant compounds in the EO of both wild and cultivated plants [28].

*S. candidissima* (Munby) Briq, commonly known as ‘Nabta beda’ or ‘Zaatercheleuh’, grows in rocky grasslands at around 400 m elevation in western Algeria. The hydrodistilled EO from dried aerial parts was dominated by pulegone (**9**) (32.1%), menthone (**11**) (23.8%) and neo-menthol (**16**) (20.2%), with oxygenated monoterpenes comprising 84.9% of the constituents [29]. Saidi et al. reported a different EO composition; their results showed that EO contained pulegone (**9**) (53.26%) as the main component, with (+)-menthone (**11**) and borneol (**17**) as minor components [30].

The blue savory (*S. coerulea* Janca), endemic to several Southeast European countries and also cultivated in Ukraine, had its EO analyzed by GC/MS, revealing that the main components were thymol (**7**) (33.18%), the unusual o-cymene (**18**) (14.42%), terpinen-4-ol (**19**) (8.30%), and γ-terpinene (**8**) (7.25%) [31].

*S. cuneifolia* [syn. *S. montana* subsp. *cuneifolia* (Ten.) O. Bolós & Vigo] is a subshrub that grows in the subtropical areas of Spain, southeast Europe, and Iraq. In the first analysis of its EO by El Beyrothy et al., the seasonal changes in EO composition were investigated. Carvacrol (**5**) (20.4–52.1%), *p*-cymene (**6**) (9.1–30.2%) and *γ*-terpinene (**8**) (5.9–23.9%) were identified as the main compounds and their concentrations showed remarkable variability during the vegetation period [32]. The EOs from the flowering aerial parts of *S. cuneifolia* obtained by HD at four different distillation times (5, 30, 60 and 180 min) in a Clevenger apparatus were analyzed by Yildiz et al. [33]. The total EO yield was 2.7% and its main components were carvacrol (**5**) (48.1%), *p*-cymene (**6**) (11.9%), *γ*-terpinene (**8**) (8.6%) and geraniol (**20**) (8.4%). A 30 min distillation time yielded the highest carvacrol (**5**) content, and a distillation duration of 60 min gave the highest EO yield [33]. Perrino et al. reported a study of *S. cuneifolia* EO originating from two provinces of South Italy. Unlike previous studies, *α*-pinene (**21**) and *α*-terpineol (**22**) were found to be the most abundant constituents. The other minor compounds (each <7%) were present in slightly varying proportions [34].

*S. hortensis* L. (summer savory) is an annual plant ranging from southeastern Europe to western Asia and is widely cultivated as a culinary herb. The EOs of summer savory have been investigated by ten research groups between 2014 and 2025. The EO composition data are summarized in Table 2. The variability in the EO chemical profiles is not as diverse as that of *S. montana*. The constituents of the EOs of *S. hortensis* were thymol (**7**), carvacrol (**5**), *α*- and *γ*-terpinene (**23**, **8**), *p*-cymene (**6**), *β*-caryophyllene (**4**), *β*-bisabolene (**30**), and myrcene (**25**). Carvacrol (**5**), *γ*-terpinene (**8**) and thymol (**7**) were identified as the predominant compounds of the EO. The solid-phase microextraction (SPME) method was applied in addition to HD extraction, with both methods yielding similar composition [35,36]. The data presented in Table 2 indicate that, for *S. hortensis*, there are two chemotypes: the carvacrol/γ-terpinene chemotype and the thymol chemotype. The EO constituent, carvacrol in its glucosylated form (carvacrol-*β-O*-glucoside), was isolated from the ethyl acetate fraction of *S. hortensis* [37].

*S. intermedia* C.A.Mey, native to the Mediterranean region and Iran, was investigated by Sharifi-Rad et al. who found that the main EO constituents were *γ*-terpinene (**8**) (37.1%), thymol (**7**) (30.2%) and *p*-cymene (**6**) (16.2%) [45]. A previous study of *S. intermedia* EO by Shadegi et al. demonstrated the same major constituents, but the relative proportions of other compounds differed (thymol (**7**) 34.5%, *γ*-terpinene (**8**) 18.2% and *p-*cymene (**6**) 10.5%) [46].

*S. isophylla* Rech. f. is a perennial species endemic to Iran. Various parts of the plant, including flowers, leaves, stems, and roots, were collected during the flowering period from the Alborz Mountains (Mazandaran Province, Iran) at an elevation of 2700 m. All plant organs contained compounds unusual in the *Satureja* genus: camphor (**27**), α-eudesmol (**28**), and elemol (**29**) as main components. In flowers and leaves, camphor (**27**) was the predominant compound (18.28% and 19.29%, respectively), while in stems and roots, α-eudesmol (**28**) predominated (28.06% and 34.68%, respectively) [47].

*S. kermanica* Payandeh, Bordbar, & Mirtadz (Kermanica savory) is a newly identified plant species that grows mainly in the Kerman province of Iran [48]. Thirty-five different components were detected by GC-MS analysis in the EO. Thymol (**7**) (46.54%) and carvacrol (**5**) (30.54%) were found to be the main compounds.

**Figure 2:**
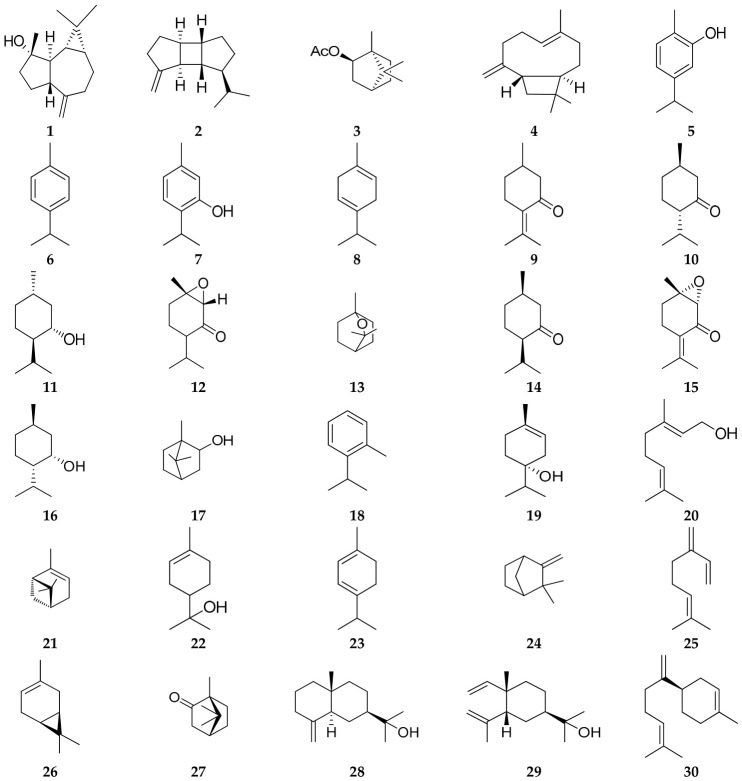

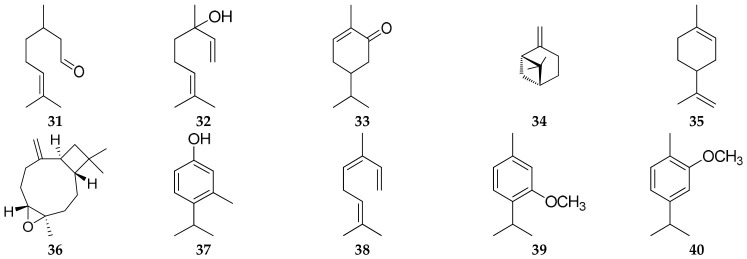
Structure of the constituents of the EO **1**–**40**.

The Iranian endemic plant *S. khuzistanica* Jamzad (Khuzestani savory) was analyzed for its EO profile. EO obtained by HD (with a yield of 1.1%) contained carvacrol (**5**) (80.55%) as the only major compound, with *p*-cymene (**6**), *β*-bisabolene (**30**), citronellal (**31**) and linalool (**32**) as minor constituents [49]. Shahmohammadi et al. investigated the EO of *S. khuzistanica* extracted by headspace-solid phase microextraction (HS–SPME). The EO composition was the same for the main compound [carvacrol (**5**) 71.99%], but differed for the minor components [camphene (**24**), *α*-pinene (**21**), 1,8-cineole (**13**), carvone (**33**) and *β*-pinene (**34**)] [50]. The high abundance of carvacrol (**5**) (94.1%) in *S. khuzistanica* EO was also documented by Mahboubi and Kazempour (Figure 2) [51].

*Satureja kitaibelii* Wierzb. ex Heuff. is a semi-shrubby perennial plant that is endemic to the Balkan Mountains (southwest Romania, eastern Serbia, and northwest Bulgaria). The investigation of the effects of plant organs and geographical origin on EO composition showed that the EO chemical profile varied considerably. Sesquiterpenes dominated EOs from floral parts, whereas monoterpenes dominated EOs from leaves and aerial parts of the plant. Plant populations of different origins showed substantial differences in EO profiles and the main compounds [linalool (**32**), *p*-cymene (**6**), geraniol (**20**)]. Literature reports indicate several potential chemotypes [linalool (**32**), geraniol (**20**), limonene (**35**)]. The presence of chemotypes in a single population (intrapopulation variability) has also been suggested, and high genetic diversity of *S. kitaibelii* has been proposed [52]. The EO composition of aerial parts of *S. kitaibelii* from a Bulgarian Danubian plain locality showed notable differences: the Kartozhabene sample was dominated by limonene (**35**), geraniol (**20**) and carvacrol (**5**), while the Kaylaka sample was mainly composed of *p*-cymene (**6**), carvacrol (**5**) and limonene (**35**). These variations may be due to microclimate, soil pH, and vegetation differences [53]. Dimitrijević et al. reported that, interestingly, minor constituents—borneol (**17**), spathulenol (**1**), caryophyllene oxide (**36**) and limonene (**35**)—and their ratios, rather than dominant compounds, are responsible for the high antibacterial activity of *S. kitaibelii* EO [54].

*S. laxiflora* K Koch (SL) is an annual plant endemic from West Asia to the Caucasus, and its EO yield was 0.6% and its composition was characterised by thymol (**7**) (24.54%), *p*-cymene (**6**) (16.95%) and *γ*-terpinene (**8**) (11.35%) as major ingredients [23].

*S. macrantha* C.A. Mey., native to western and northern Iran, is used in Iran primarily as a sweetener and for the treatment of urinary tract diseases. Hydrodistilled EO from aerial parts collected in Iran was found to be dominated by *p*-cymene (**6**) (45.8%) as the main compound and borneol (**17**), carvacrol (**5**) and α-pinene (**21**) as minor compounds [47].

*S. montana* L. (winter savory) is native to the Mediterranean area, but is cultivated throughout Europe. *S. montana* is a perennial semi-shrub that grows in rocky, sunny, and arid areas; it is one of the most studied and most popular species of the *Satureja* genus. In the years 2014–2023, eight articles on the phytochemistry of *S. montana EO* and its subspecies were published. The main findings of these publications are summarized in Table 3. A literature report has revealed large variations in the abundance of the main components, such as thymol (**7**), *p*-thymol (3**7**), carvacrol (**5**), borneol (**17**) and *α*-pinene (**21**) and enormous variability in the minor components. The EO content varied between 0.9% and 1.5%. Hudz et al. (2020) found for the first time that *p*-thymol (**37**) is the dominant compound of EO in the aerial part of the winter savory [55]. The leaves of *S. montana* L. from Brazil were rich in borneol (**17**) [56]. The comparison of the EO chemical composition between *S. montana* subsp. *variegata* and *S. montana* subsp. *montana* cultivated in Northern Italy revealed the same main compounds, but in substantially different ratios [57]. Zawislak et al. analyzed the relationships between agronomic factors (plant density and number of harvests) and selected parameters of the raw material yield of winter savory. Among the agronomic factors studied, the number of harvests and harvest date had a greater effect on fresh and dry winter savory herb yields than the density of the planting. The EO content (1.44 to 2.04%) of the plant material did not depend on the number of harvests or the harvest date. The composition of EO was not investigated in this study [58].

*S. mutica* Fisch. & C.A.Mey., occurring in the Transcaucasus, northern Iran, and southern Turkmenistan, is a perennial, relatively woody shrub. An investigation by Karimi et al. assessed the levels of inter- and intrapopulation variability of the EO constituents of the aerial parts. A high level of variability was observed among the seven populations studied and their individual plants. The EO yield varied from 0.5% to 4.2% (*w*/*w*). The EOs of the different plants included in the study contained thymol (**7**) (6.5–74.6%), carvacrol (**5**) (0.9–70.4%), borneol (**17**) (0.1–10.9%), *p*-cymene (**6**) (0.30–14.2%) and *γ*-terpinene (**8**) (0.1–9.9%) as main constituents [64]. The EO composition obtained by HD from dried seeds of *S. mutica* contained carvacrol (**5**) (64.0%), *p*-cymene (**6**) (12.1%) and *γ*-terpinene (**8**) (6.2%) as the main compounds [65].

*S. nabateorum* Danin and Hedge is a perennial plant discovered in 1998. The plant has a limited distribution in certain regions of Jordan and Palestine. The first study on the EO composition of *S. nabateorum* was published in 2022 by Al-Maharik and Jaradat. An ultrasonic microwave apparatus was used to isolate volatile compounds from fresh and air-dried aerial parts. Thymol (**7**) (46.07 ± 1.1% and 40.64 ± 1.21%) was the major compound of the EO, followed by *γ-*terpinene (**8**) and *p*-cymene (**6**) with a slight variation in their relative abundances [66].

*S. parnassica* Heldr. Sart. ex Boiss. is a plant endemic to central and southern Greece. It has been well known as a spice and herbal remedy since ancient times. The distilled EO contained carvacrol (**5**) (33.72%), thymol (**7**) (17.82%), *p*-cymene (**6**) (10.32%) and *γ*-terpinene (**8**) (15.47%) are the main compounds, consistent with previously published data [67].

*S. rechingeri* Jamzad (rechingeri savory) is an endemic species of Iran. Air-dried plant material was subjected to HD, producing an EO yield of 1.25% EO, which contained *p*-cymene (**6**) (46.5%) as the main compound and *γ*-terpinene (**8**) (8.1%), limonene (**35**) (6.3%), thymol (**7**) (6.2%) and carvacrol (**5**) (4.2%) as minor constituents [68]. In another study, the EO extracted from leaves by the same method was found to have carvacrol (**5**) (86.91%) as the main constituent [69].

*S. sahendica* Bornm. (locally called Marze Sahandi) is an endemic species of Iran, distributed in the western and northwestern regions. The EO (yield of 3.5%) contained 27 identified compounds, with thymol (**7**) (49.23%), *p*-cymene (**6**) (20.12%), and γ-terpinene (**8**) (15.23%) as dominant constituents [70].

*S. subspicata* Vis. (Dwarf savory) is a rare perennial semi-shrubby plant, an endemic Dinaric species that lives at altitudes between 200 and 1800 m. The EO of *S. subspicata* growing in Bosnia and Herzegovina contained non-oxygenated monoterpenes (46.6%) as primary classes of EO. The major EO constituents were: *β*-caryophyllene (**4**), *cis*-*β*-ocimene (**38**) and *α*-pinene (**21**). When comparing the findings of this investigation with those previously published for *S. subspicata*, a pronounced variation was observed in the composition of the EO was observed. These variations may be due to environmental and geographic variance and different stages of plant development [71].

*S. thymbra* L., known as pink savory, is endemic to the Mediterranean region, including Libya [72]. In a study by Khalil et al. the influence of the altitude of the plant collection site on the EO content and composition was evaluated. The EO was obtained from plants originating from two different altitudes (156 and 661 m above sea level) in two consecutive years. The EO contents showed only slight differences between plants collected at lower and higher altitudes. More substantial differences were observed in EO composition. Thymol (**7**) (26.69%) was the major compound in plants collected at lower altitudes, whereas carvacrol (**5**) (14.30%) predominated at higher altitudes. These results suggest that elevation plays an important role in the plant health and growth of plants and has a significant effect on EO production. The *S. thymbra* EO obtained from cultivation on Icaria Island, Greece, contained 32.8% carvacrol (**5**) as a major compound [73].

*Satureja thymbrifolia* Hedge & Feinbrun, endemic to Pakistan, grows in the Al-Naqab Desert and along the shores of the Dead Sea. Local Bedouins traditionally use it as a natural antibiotic. Its EO [yield 1.57% (*w*/*w*)] was analyzed by GC-MS, revealing *p*-cymene (**6**) (48.53%), thymol (**7**) (23.27%), and α-terpineol (**22**) (7.42%) as the main components [74].

*S. viminea* L. is an aromatic species popularly known as mint or hibiscus mint, native to America. In the EO of *S. viminea*, pulegone (**9**) (37.40%), β-caryophyllene (**4**) (11.33%) and *p*-menth-3-en-8-ol (11.83%) were identified as the main constituents [75].

In summary, during the study period, essential oils (EOs) were the most extensively investigated metabolites of the *Satureja* genus. Current research on *Satureja* EOs is characterized by chemotype identification, population-level comparisons, and investigations of environmental and methodological drivers of chemical diversity. Numerous variability studies demonstrated strong inter- and intrapopulation differences influenced by geography, altitude, plant organs, phenological stage, and genetic factors. Comparative studies further evaluated wild versus cultivated plants, seasonal variation, harvest time, and agronomic factors, generally showing that EO composition is more sensitive to environmental and genetic influences than EO yield. Methodological comparisons in several species demonstrated that hydrodistillation, microwave-assisted techniques, and SPME yield similar qualitative profiles while differing in efficiency and sustainability. Geographical comparisons consistently revealed major compositional differences between populations and subspecies in the studied species.

### 3.2. Flavonoids

**Figure 3:**
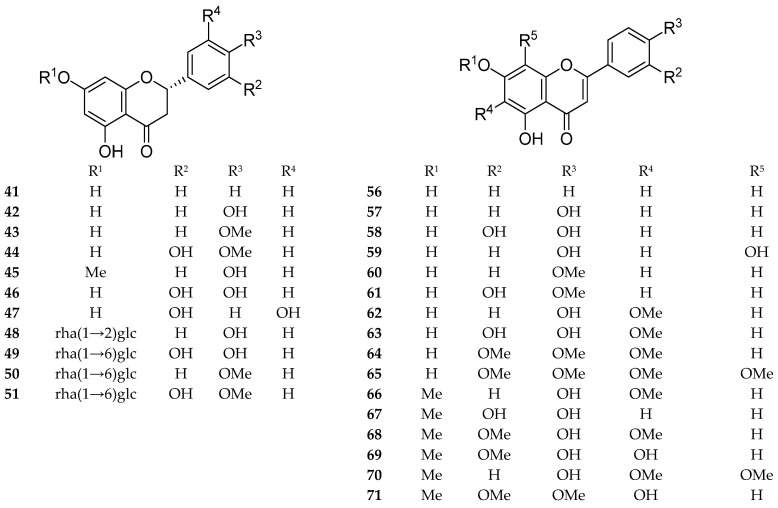

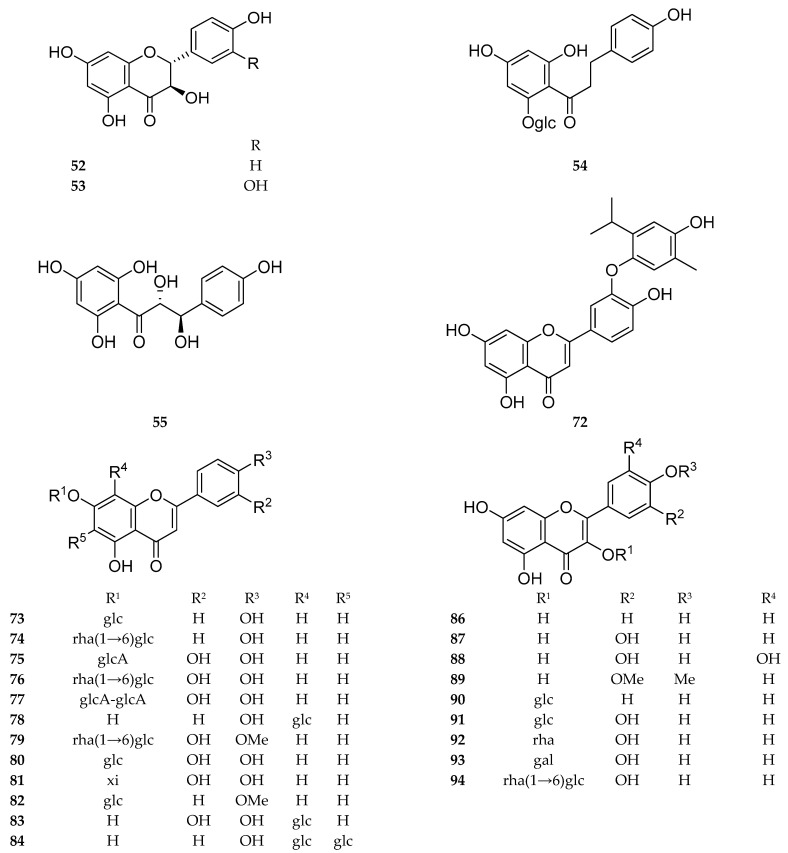

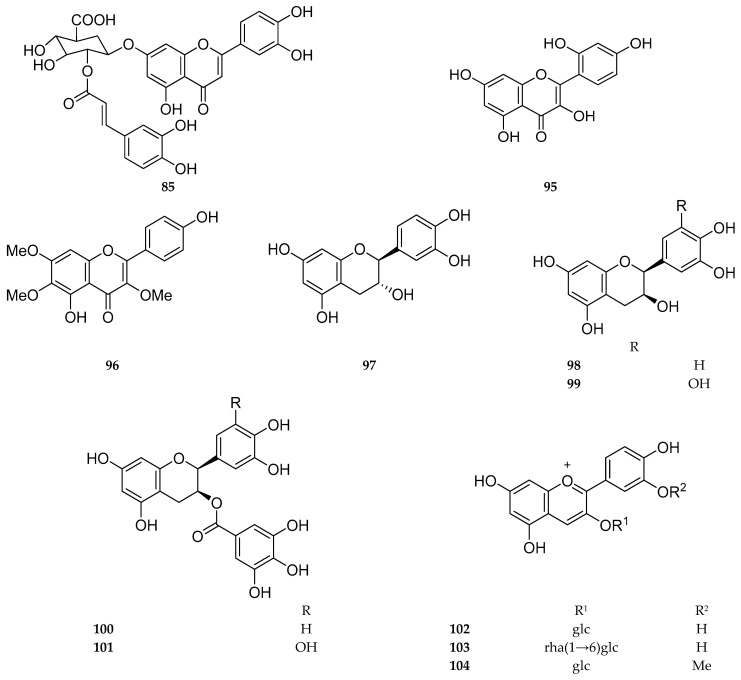
Structure of flavonoids identified in *Satureja* species.

During the period covered by this review, 64 flavonoids were described from various species of the *Satureja* genus (Figure 3). A list of the compounds identified is shown in Table 4. The reported flavonoids belong to the flavanone, flavanonol, dihydrochalcone, flavone, flavonol, catechin, and anthocyanin types; glycosides and aglycones occurred equally. The flavonoids that occurred most frequently were apigenin (**57**), luteolin (**58**), quercetin (**87**), and naringenin (**42**); these compounds were described in 12 to 13 *Satureja* species. *S. kitaibelii* was shown to be the species richest in flavonoids because 19 compounds belonging to the flavone, flavonol, and flavanonol groups were reported from this species, most of which were identified by liquid chromatography photodiode array/mass spectrometry (LC-PDA/MS method) [76,77]. LC-MS/MS analysis of *S. cuneifolia* extract allowed the detection of apigenin (**57**), luteolin (**58**), quercetin (**87**), naringenin (**42**), hesperetin (**44**), eriodictyol (**46**) and/or their glycosides in the aerial parts [33]. A series of flavonoids were identified in the methanolic extract *of S. hortensis* by the ultrahigh performance liquid chromatography (UHPLC) coupled with diode-array detection (DAD) and high-resolution electrospray ionization tandem mass spectrometry (HRESI-MS/MS) methods, including naringenin (**42**) in relatively high levels, and other flavonoids as minor compounds [78].

LC–MS/MS analysis of *S. macrantha* revealed the presence of different types of flavonoids [hesperidin (**51**), quercetin (**87**), and keracyanin chloride (**103**)] as key constituents [44]. In *S. pilosa*, LC-HRMS analysis revealed that flavanones and flavones—namely hesperidin (**51**), luteolin 7-*O-*rutinoside (**76**), naringenin (**42**), naringin (**48**), hispidulin (**62**), and penduletin (**96**)—were the predominant flavonoids [79].

Saturejin (**72**) is a conjugate of luteolin and 2,5-dihydroxy-*p*-cymene. It was discovered in *S. khuzistanica* along with 12 other flavonoids, including flavonols [aromadendrin (**52**) and taxifolin (**53**)], flavanones [naringenin (**42**) and 5,7,3′,5′-tetrahydroxyflavanone (**47**)] and flavones [xanthomicrol (**70**), acacetin (**60**), cirsimaritin (**66**), 7-methylluteolin (**67**), apigenin (**46**), cirsilineol (**68**), diosmetin (**61**) and 6-hydroxyluteolin 7,3′-dimethyl ether (**69**)] [80].

Esterified flavonoids were found in the extracts of *S. biflora* and *S. kitaibelii*. 2″-caffeoyl-luteolin 7-*O*-*β*-d-glucuronide (**85**) was isolated from the aerial parts of the African plant *S. biflora* [81]. *p*-Coumaroyl-, caffeoyl- and sinapoyl-substituted luteolin-dihexuronide could be identified in *S. kitaibelii*. The positions of the caffeoyl and sinapoyl groups could not be determined in tandem ultra-high performance liquid chromatography/detection of diodes array/high resolution electrospray ionisation mass spectrometry [UHPLC/DAD/(+/−)HRESI-MS/MS] identification [77].

**Table 4:** Flavonoids identified in *Satureja* species (* compound identified by LC-MS without determining the quality and position of the sugar part).

**Compound Name**	**Plant Species**	**Reference**
**Flavanones**		
Pinocembrin (**41**)	*S. horvatii*, *S. subspicata*	[82]
Naringenin (**42**)	*S. hortensis*, *S. khuzistanica*, *S. cuneifolia*, *S. kitaibelii*, *S. boissieri*, *S. horvatii*, *S. subspicata*, *S. macrantha*, *S. aintabensis*, *S. spicigera*, *S. pilosa*, *S. montana*, *S. bachtiarica*	[33,44,71,77,78,79,80,82,83,84,85,86,87]
Isosakuranetin (**43**)	*S. aintabensis*, *S. spicigera*, *S. pilosa*	[79,85]
Hesperetin (**44**)	*S. boissieri*	[84]
Genkwanin (**45**)	*S. kitaibelii*, *S. bachtiarica*	[77,87]
Eriodictyol (**46**)	*S. subspicata*, *S. cuneifolia. S. kitaibelii*, *S. bachtiarica*	[33,71,77,87]
5,7,3′,5′-Tetrahydroxyflavanone (**47**)	*S. khuzistanica*	[80]
Naringin (**48**)	*S. cuneifolia*, *S. hortensis*, *S. macrantha; S. aintabensis*, *S. spicigera*, *S. pilosa*	[33,44,78,79,85,88]
Eriocitrin (**49**)	*S. cuneifolia*	[33]
Isosakuranetin 7-*O*-rutinoside (**50**)	*S. bachtiarica*	[87]
Hesperidin (**51**)	*S. cuneifolia*, *S. boissieri*, *S. macrantha*, *S. aintabensis*, *S. spicigera*, *S. pilosa*, *S. bachtiarica*	[33,44,79,84,85,87]
**Flavanonols**		
Aromadendrin (**52**)	*S. khuzistanica*, *S. aintabensis*, *S. spicigera*, *S. pilosa*, *S. bachtiarica*	[79,80,85,87]
Taxifolin (**53**)	*S. khuzistanica*, *S. aintabensis*, *S. spicigera*, *S. pilosa*	[79,80,85]
**Dihydrochalcon**		
Phlorizin (**54**)	*S. hortensis*	[78]
Cilicione-a (**55**)	*S. hortensis*	[41]
**Flavones**		
Chrysin (**56**)	*S. horvatii*, *S. subspicata*, *S. pilosa*	[79,82]
Apigenin (**57**)	*S. subspicata*, *S. kitaibelii*, *S. boissieri*, *S. hortensis*, *S. bachtiarica*, *S. sahendica*, *S. horvatii*, *S. subspicata*, *S. khuzistanica*, *S. aintabensis*, *S. spicigera*, *S. hasturkii*, *S. pilosa*	[36,71,77,78,79,80,82,84,85,87,88,89,90,91]
Luteolin (**58**)	*S. subspicata*, *S. kitaibelii*, *S. cuneifolia*, *S. hortensis*, *S. boissieri*, *S. bachtiarica*, *S. sahendica*, *S. macrantha*, *S. aintabensis*, *S. spicigera*, *S. hasturkii*, *S. pilosa*	[33,44,71,77,78,79,84,85,87,88,89,90,91]
Isoscutellarein (**59**)	*S. montana*	[92]
Acacetin (**60**)	*S. khuzistanica*, *S. aintabensis*, *S. spicigera*	[80,85]
Diosmetin (**61**)	*S. khuzistanica*, *S. subspicata*	[71,80]
Hispidulin (**62**)	*S. aintabensis*, *S. spicigera*, *S. pilosa*	[79,85]
Nepetin (**63**)	*S. aintabensis*	[85]
Eupatilin (**64**)	*S. bachtiarica*	[87]
Hymenoxin (**65**)	*S. bachtiarica*	[87]
Cirsimaritin (**66**)	*S. khuzistanica*	[80]
7-Methylluteolin (**67**)	*S. khuzistanica*	[80]
Cirsilineol (**68**)	*S. khuzistanica*	[80]
6-Hydroxyluteolin 7,3′-dimethyl ether (**69**)	*S. khuzistanica*, *S. hortensis*	[36,80]
Xanthomicrol (**70**)	*S. khuzistanica*	[80]
5,6-Dihydroxy-7,3′,4′-trimethoxyflavone (**71**)	*S. hortensis*	[41]
Saturejin (**72**)	*S. khuzistanica*	[80]
Apigenin 7-*O*-glucoside (**73**)	*S. hortensis*, *S. pilosa*, *S. coerulea*, *S. aintabensis*, *S. spicigera*, *S. bachtiarica*	[31,78,79,85,87]
Apigenin 7-*O*-rutinoside (**74**)	*S. kitaibelii*, *S. cuneifolia*, *S. pilosa*, *S. bachtiarica*	[33,76,79,87]
Apigenin glycoside *	*S. hortensis*	[88]
Apigenin glucuronide *	*S. cuneifolia*	[33]
Apigenin deoxyhexosylhexoside *	*S. kitaibelii*	[77]
Apigenin dihexuronide *	*S. kitaibelii*	[77]
Apigenin C-dihexoside *	*S. montana*	[93]
Luteolin 7-*O*-glucuronide (**75**)	*S. kitaibelii*, *S. biflora*, *S. bachtiarica*	[76,81,87]
Luteolin 7-*O*-rutinoside (**76**)	*S. kitaibelii*, *S. pilosa*, *S. bachtiarica*, *S. aintabensis*, *S. spicigera*	[76,77,79,85,87]
Luteolin 7-O-diglucuronide (**77**)	*S. kitaibelii*	[77]
Vitexin (**78**)	*S. hortensis*	[78]
Diosmin (**79**)	*S. kitaibelii*	[76,77]
Luteolin 7-*O*-glucoside (**80**)	*S. aintabensis*, *S. spicigera*, *S. hasturkii*, *S. pilosa*, *S. coerulea*	[31,79,85,91]
Luteolin 7-*O*-xyloside (**81**)	*S. bachtiarica*	[87]
Luteolin C-dihexoside *	*S. montana*	[93]
Luteolin caffeoyl-dihexuronide *	*S. kitaibelii*	[77]
Luteolin p-coumaroyl-dihexuronid *	*S. kitaibelii*	[77]
Luteolin sinapoyl-dihexuronide *	*S. kitaibelii*	[77]
Luteolin glucoside *	*S. cuneifolia*	[33]
Luteolin glucuronide *	*S. cuneifolia*, *S. montana*	[33,93]
Luteolin *O*-diglucuronide *	*S. montana*	[93]
Luteolin rutinoside *	*S. cuneifolia*	[33]
Luteolin-glycoside *	*S. hortensis*	[88]
Apigenin 7-*O*-β-d-glucoside 4′-*O*-methyl ether (**82**)	*S. bachtiarica*	[87]
Methylapigenin deoxyhexosyl-hexoside *	*S. kitaibelii*	[77]
Methylapigenin hexoside *	*S. kitaibelii*	[77]
Methylapigenin rutinoside *	*S. kitaibelii*	[76]
Orientin (**83**)	*S. aintabensis*, *S. spicigera*, *S. pilosa*	[79,85]
Vicenin 2 (**84**)	*S. bachtiarica*	[87]
2′’-Caffeoylluteolin 7-*O*-β-d-glucuronide (**85**)	*S. biflora*	[81]
**Flavonols**		
Kaempferol (**86**)	*S. hortensis*, *S. subspicata*, *S. boissieri*	[71,83,84,88]
Quercetin (**87**)	*S. subspicata*, *S. montana*, *S. hortensis*, *S. cuneifolia*, *S. bachtiarica*, *S. sahendica*, *S. horvatii*, *S. macrantha*, *S. aintabensis*, *S. spicigera*, *S. pilosa*, *S. coerulea*	[8,31,33,44,78,79,82,83,85,86,88,89,94]
Myricetin (**88)**	*S. subspicata*	[71]
Quercetin 3′,4′-dimethyl ether (**89**)	*S. bachtiarica*	[87]
Astragalin (**90**)	*S. hortensis*, *S. coerulea*, *S. bachtiarica*	[31,78,87]
Isoquercitrin (**91**)	*S. kitaibelii*, *S. hortensis*, *S. pilosa*, *S. coerulea*, *S. bachtiarica*	[31,76,77,78,79,87]
Quercitrin (**92**)	*S. hortensis*	[78]
Hyperoside (**93**)	*S. boissieri*, *S. pilosa*	[79,84]
Rutin (**94**)	*S. subspicata*, *S. montana*, *S. boissieri*, *S. hortensis*, *S. bachtiarica*, *S. sahendica*, *S. coerulea*	[8,31,55,84,88,89,94]
Morin (**95**)	*S. hortensis*	[83]
Penduletin (**96**)	*S. aintabensis*, *S. spicigera*, *S. pilosa*	[79,85]
Quercetin *O*-glucuronide *	*S. montana*	[93]
Eriodictyol rutinoside *	*S. kitaibelii*	[76]
Hesperidin rutinoside *	*S. cuneifolia*	[33]
Quercetin rutinoside *	*S. cuneifolia*	[33]
**Catechins**		
(–)-Catechin (**97**)	*S. montana*	[55]
Epicatechin (**98**)	*S. montana*	[86]
Epigallocatechin (**99**)	*S. barceloi*, *S. pilosa*	[79,95]
Epicatechin 3-*O*-gallate (**100**)	*S. barceloi*	[95]
Epigallocatechin 3-*O*-gallate (**101**)	*S. barceloi*, *S. pilosa*	[79,95]
**Anthocyanins**		
Cyanidin-3-*O*-glucoside (**102**)	*S. macrantha*	[44]
Keracyanin chloride (**103**)	*S. macrantha*	[44]
Peonidin-3-*O*-glucoside (**104**)	*S. macrantha*	[44]

* Structures were not fully determined.

### 3.3. Phenolic Acids

**Figure 4:**
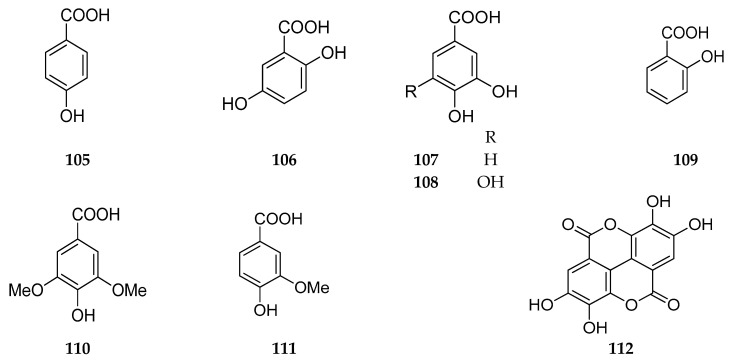

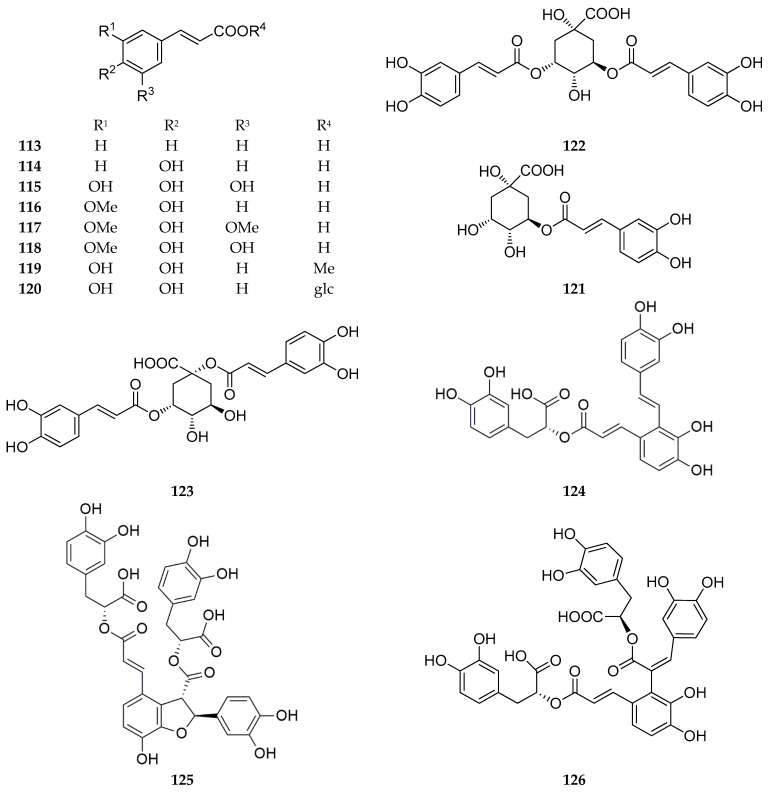

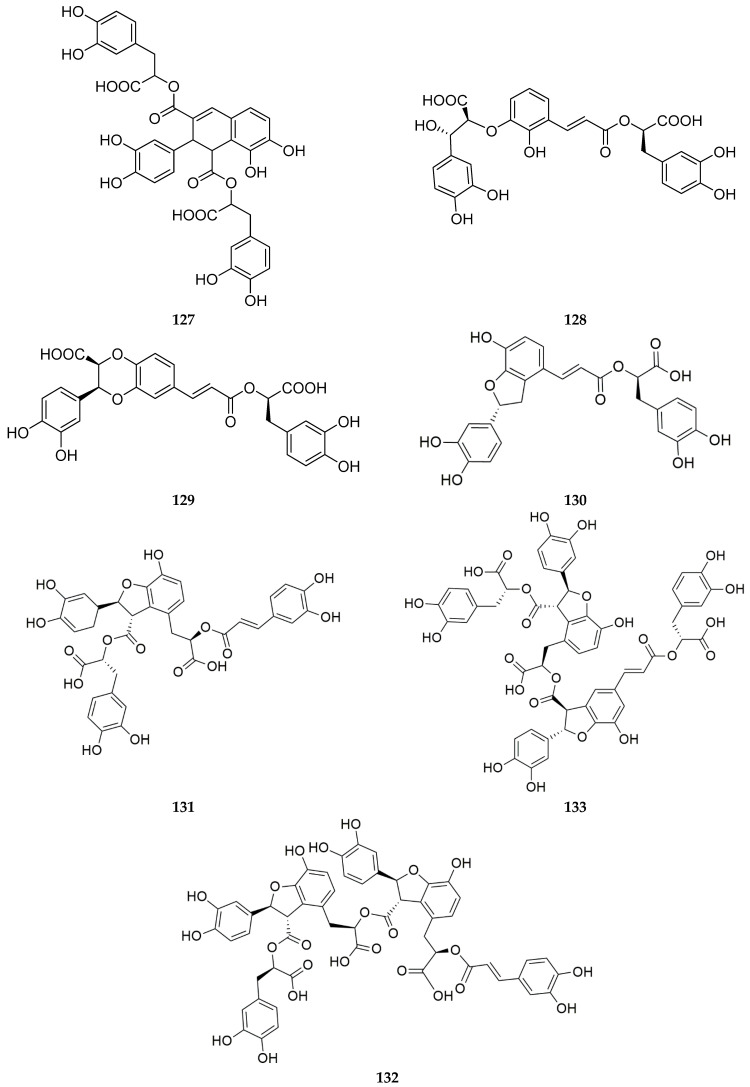

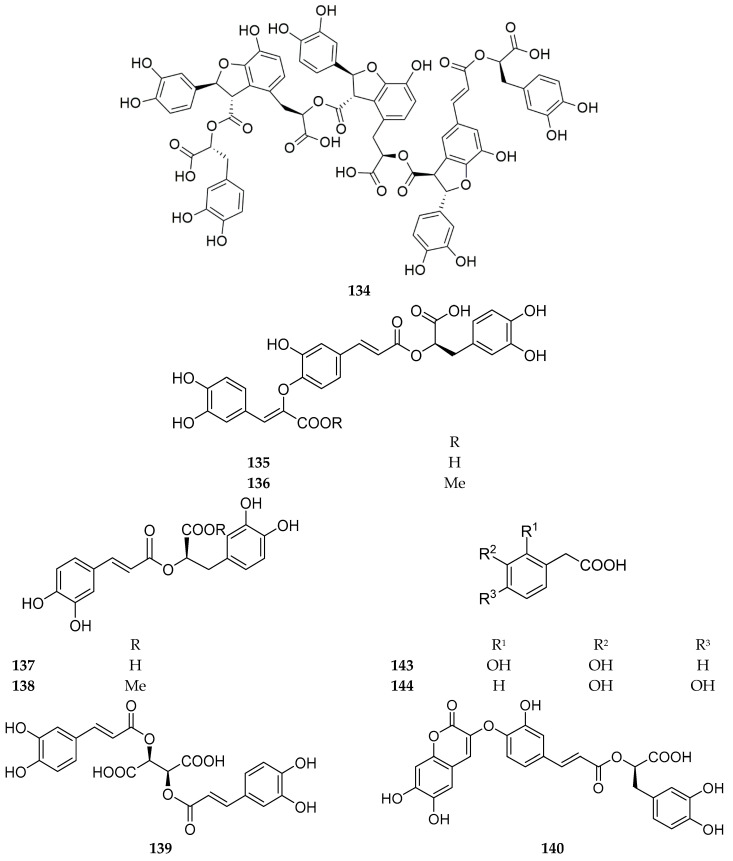

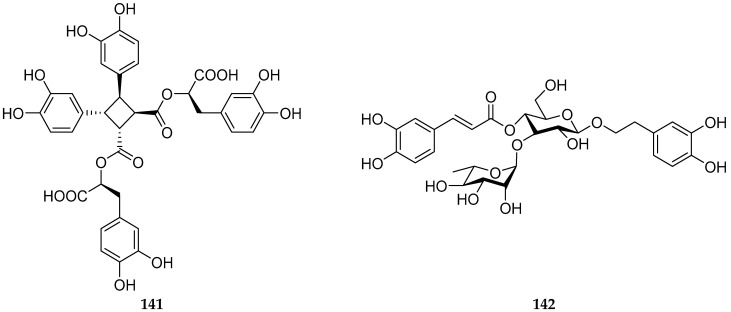
Structures of the phenolic acids and their derivatives of *Satureja* species.

From *Satureja* species, 40 phenolic acid compounds were identified (Table 5). The identified compounds were benzoic acid derivatives (**105**–**111**), cinnamic acid derivatives (**113**–**142**) and hydroxylated phenylacetic acids (**143**, **144**) (Figure 4). Simple aromatic acids, such as 4-hydroxybenzoic acid (**105**), gentisic acid (**106**), protocatechuic acid (**107**), gallic acid (**108**), salicylic acid (**109**), syringic acid (**110**), and ellagic acid (**112**), were identified in a total of eleven species of *Satureja* genus by photo-diode-array (PDA), MS-coupled HPLC or GC-MS. Syringic acid (**110**) and vanillic acid (**111**) were found in ten and eight species, respectively.

Cinnamic acid (**113**) and its hydroxylated and methoxylated derivatives (**114**–**118**) were detected in *S. aintabensis*, *S. bachtiarica*, *S. boissieri*, *S. hasturkii*, *S. hortensis*, *S. horvatii*, *S. kitaibelii*, *S. macrantha*, *S. montana*, *S. sahendica*, *S. spicigera*, *S. subspicata*, and *S. pilosa*. Similarly abundant were the quinic acid derivatives coupled with caffeic acid—chlorogenic acid (**121**), 3,5-caffeoylquinic acid (**122**), and cynarin (**123**)—identified in ten *Satureja* species. The most frequently occurring cinnamic acid derivatives were caffeic acid (**115**), chlorogenic acid (**121**), and rosmarinic acid (**137**), which were detected in thirteen, ten, and sixteen *Satureja* species, respectively.

Characteristic compounds of the genus are hydroxycinnamic acid oligomers (**124**–**141**) that rarely occur in nature. These compounds comprise a series caffeic acid derivatives of increasing complexity, where the caffeoyl residues form dimeric [rosmarinic acid (**137**)], trimeric [salvianolic acid A (**124**), K (**128**), isosalvianolic acid A (**130**), melitric acid A (**135**), methyl melitric acid A (**136**), sagecoumarin (**141**)], tetrameric [salvianolic acid B (**125**), E (**126**), L (**127**), clinopodic acid I (**131**)], hexameric [clinopodic acid K (**132**) and O (**133**), and sagerinic acid (**140**)] and octameric [clinopodic acid P (**134**)] structures through esterification and Diels–Alder reactions. An advanced HPLC-MS^n^ method was used to identify these compounds. Sagecoumarin (**141**) is a caffeic acid tetramer containing a coumarin moiety. Among the caffeic acid derivatives, rosmarinic acid (**137**) was present in the highest amounts in the extracts of *S. subspicata*, *S. kitaibelii*, *S. hortensis*, *S. biflora*, *S. pilosa*, *S. macrantha*, and *S. avromanica* [21,40,44,71,76,79,81,82]. In *S. bachtiarica*, *S. cuneifolia*, *S. kitaibelii*, and *S. spicigera*, compounds in which caffeic acid is coupled with a sugar unit (**120**, **142**, rosmarinic acid glucuronide, and rosmarinic acid hexoside) were identified [33,76,85,87]. GC-MS analysis of a polar fraction of *S. montana* detected 2,3-dihydroxyphenylacetic acid (**104**) and 3,4-dihydroxyphenylacetic acid (**105**) [96].

### 3.4. Jasmonates

Jasmonates are phospholipid-derived hormones that regulate plant development and responses to environmental stress. *Z*-jasmone was identified as an EO constituent of *S. calamintha* ssp. *nepeta* present at 0.3963% in the extracts [27]. LC-PDA/MS analysis of the *S. kitaibelii* extracts revealed the presence of jasmonic acid derivatives: 12-*O*-hexosyljasmonate, 12-hydroxyjasmonic acid 12-*O*-hexoside, 12-*O*-(caffeoylhexosyl)-jasmonate and 12-*O*-(methylcaffeoyl)hexosyl-jasmonate (Table 6). 12-Hydroxyjasmonic acid 12-*O*-hexoside was common in all plant parts (stems, leaves, and flowers) [76,77]. *Z*-jasmone is a volatile compound with insect-attractant or insect-repellent activity, whereas the primary role of jasmonic acid and its derivatives is to control how plants respond to biotic and abiotic stressors, as well as how they grow and develop. The presence of 12-hydroxyjasmonic acid (**146**), tuberonic acid-12-*O*-[6’-O-(*E*)-feruloyl]-*β*-d-glucopyranoside (**147**) and 12-hydroxyjasmonic acid-(6’-*O*-caffeoyl)-glucoside (**148**) was detected by LC-MS in the *n*-BuOH extract of *S. bachtiarica* [87].

### 3.5. Diterpenes, Triterpenes, and Steroids

**Figure 5:**
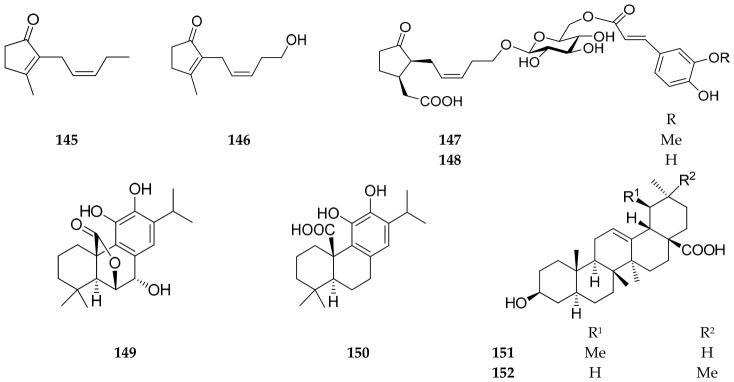

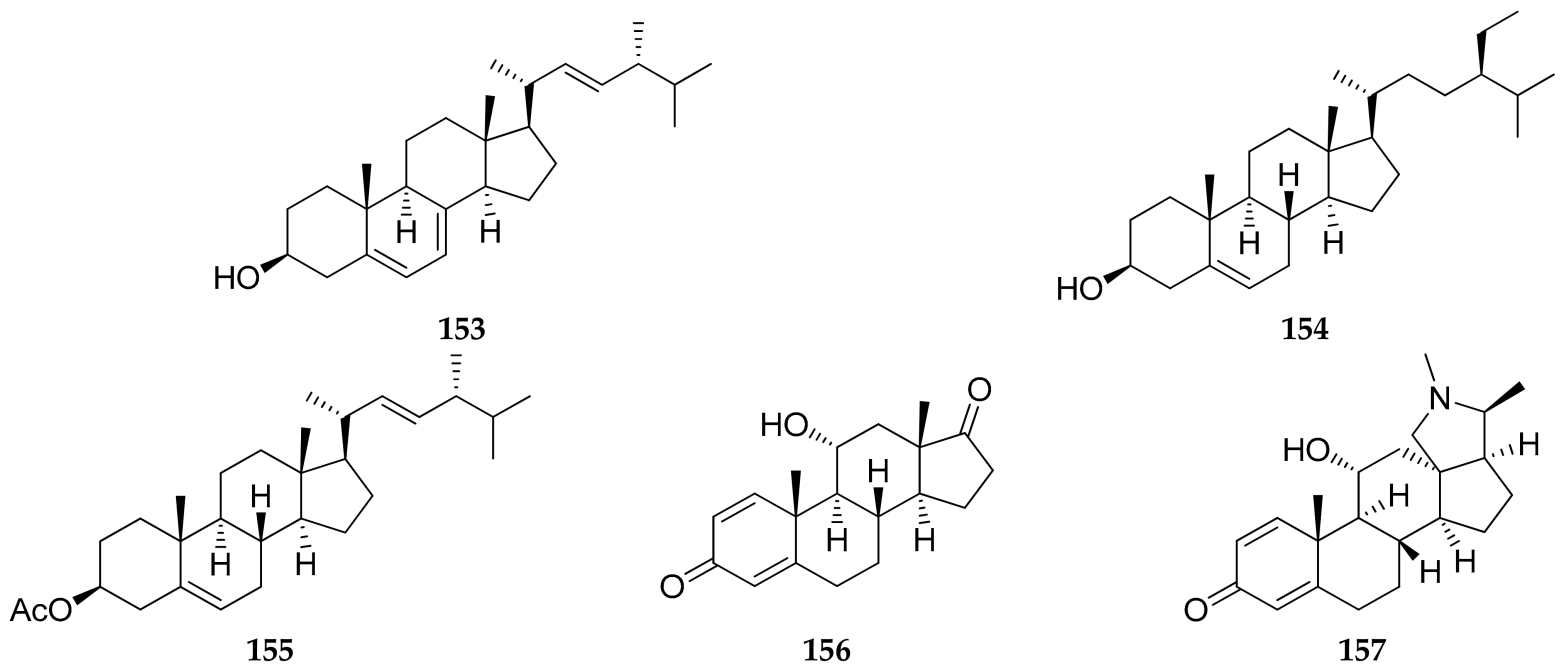
Structure of jasmones, diterpenes, triterpenes, and steroids of *Satureja* species.

Although diterpenes occur in the *Satureja* genus, only one diterpene has been described in the years 2014–2025. Rosmanol (**149**) was detected in *S. kitaibelii* extracts by LC-PDA-MS [76,77]. The concentrations of ergosterol (**153**) (69.41 ± 1.75 µg/g) and *β*-sitosterol (**154**) (19.81 ± 1.14 µg/g) in an 80% methanolic extract of *S. hortensis* seeds were determined [83] and ursolic acid (**151**) and oleanolic acid (**152**) were isolated from the aerial parts of *S. montana* [92]. GC-MS analysis of the aerial part of an *S. hortensis* ethanolic extract identified (3*β*,22*E*)-ergosta-5,22-dien-3-acetate (**155**) (2.96%), 11*α*-hydroxyandrosta-1,4-diene-3,17-dione (**156**) (1.39%) and 3-oxo-20-methyl-11*α*-hydroxyconanine-1,4-diene (**157**) (1.05%) [11] (Figure 5 and Table 6).

### 3.6. Other Compounds

Amino acids, simple organic acids and alcohols, monoacylglycerols, and carbohydrates were identified from *S. montana* by GC-MS [96]. Aliphatic alcohols, acids, esters, fatty acids and vitamin-related compounds (menadione, tocopherols, ergocalciferol, cholecalciferol, retinol) were reported from aerial parts and seeds of *S. hortensis* [11,83]. This information can be useful to assess the nutritional value of these plants. Furthermore, Alburqan et al. isolated a rarely occurring unsaturated hydroxyl fatty acid, fulgidic acids, and 3-hydroxytyrosol from *S. hortensis* [41]. Thymoquinone has been reported from *S. montana*, while two coumarins (aesculetin and aesculin) have been reported *from S. hortensis* [78,98]. Thymoquinol 5-*O*-β-d-glucopyranoside, the lignan globoidnan A and four biphenyl-type compounds were detected by LC-MS in the *n*-BuOH extract of *S. bachtiarica* [87].

A low amount of resveratrol was detected by LC–MS/MS analysis in *S. macrantha* water and methanol extracts [44]. The antifungal peptide Skh-AMP1 with a molecular weight of 2778.10 Da was purified from leaves of *S. khuzistanica* by reverse phase HPLC and sequenced by de novo sequencing and Edman degradation [99].

## 4. Pharmacology

### 4.1. Antibacterial Activity

Table S1 presents the antimicrobial activity of members of the *Satureja* genus. The table shows 64 investigations of the antibacterial activity of 22 different plant species. Most of the studies used in vitro methods (disc diffusion, broth microdilution, checkerboard microtiter assay) for evaluation of the antibacterial activity; therefore, these data provide low-to-moderate level of evidence and should be interpreted as preliminary findings. According to the literature search results, the species most frequently assessed for their antibacterial activity were *S. hortensis*, *S. khuzistanica*, and *S. montana*. Gram-negative and Gram-positive bacteria were examined simultaneously in most investigations.

Most reports have stated that the primary sign of antibacterial action is the presence and concentration of carvacrol (**5**) and strong antibacterial activity was observed in plant species with significant contents of thymol (**7**) and carvacrol (**5**). In most previous studies, conventional qualitative techniques, such as the agar-well and/or agar-disc diffusion method, in addition to the minimum inhibitory concentration (MIC) and minimum bactericidal concentration tests, were used to examine antibacterial activities. The use of novel approaches, including coating target material onto nanoparticles, has become popular in recent years. Silver nanoparticles loaded with *S. rechingeri* extract have been described by Narchin et al. [100] in terms of their antimicrobial efficacy against *Staphylococcus aureus* and *Escherichia coli* as well as their physical, optical, mechanical, barrier, and cytotoxic properties. Microbial growth was successfully inhibited by EO-containing nanoparticles. In another study, kefiran-carboxymethyl cellulose biocomposite films incorporated with EO of *S. khuzistanica* exhibited inhibitory effects against *S. aureus* and *E. coli* bacteria, indicating their potential as antimicrobial materials [49]. In another study, *S. hortensis* EO encapsulated in nanoliposomes showed strong antibacterial activity against *E. coli* (MIC = 5.187 µg/µL), *P. aeruginosa* and *S. aureus* (MIC = 2.59 µg/µL), comparable to free oil, and reduced DNA damage in human lymphocytes, indicating genoprotective properties and antibacterial safety [101].

Some EOs have been found to possess inhibitory effects on bacterial biofilm formation. *S. montana* EO inhibited the biofilm formation by >85% at the MIC (*p* < 0.05), in addition to its strong antibacterial activity [102], while *S. hortensis* EO showed significant inhibitory effects on biofilm formation and disrupted preformed *S. aureus* biofilms at sub-MICs [103]. These effects are largely attributed to phenolic monoterpenes such as carvacrol (**5**) and thymol (**7**), together with their precursors (*p*-cymene and γ-terpinene), which can disrupt bacterial membranes, alter permeability, and act synergistically to improve antimicrobial efficacy, as well as interfere with bacterial adhesion and biofilm development [102,103]. *S. montana* EO with a high thymol (**7**) content demonstrated concentration-dependent modulation of *Pseudomonas aeruginosa* growth, biofilm formation, and virulence, suggesting its potential as an antivirulence agent. A biphasic effect was observed: higher concentrations significantly inhibited planktonic growth (36–58% reduction; *p* < 0.05), while lower concentrations promoted it; therefore, careful dosing is required [104].

The synergistic effect of the EOs of some species of *Satureja* with other agents was investigated in several studies. As investigated by Mandalakis et al., EO mixtures containing *S. thymbra* and other native Mediterranean aromatic plants from Greece were among the most effective inhibitors of bacterial growth against the fish bacterial pathogen *Aeromonas veronii* bv.* sobria*, resulting in complete eradication [105]. This observation highlights the potential of these blends as alternative antibacterial agents in aquaculture. Furthermore, the anti-*Helicobacter pylori* activity of a mixture composed of *S. hortensis* and *Origanum vulgare* subsp.* hirtum* EO was evaluated in a mouse model and successfully eradicated the pathogen in 70% of mice [106]. In a recent study, the combination of *S. montana* and *Cinnamomum zeylanicum* EO exhibited a strong synergistic effect against *Salmonella enterica* serovar Typhimurium [107]. Furthermore, combining carvacrol (**5**) and *S. khuzestanica* EO and gentamicin decreased the MIC value of gentamicin from 2.3 to 0.0625 µg/mL when tested against clinical isolates of *E. coli*, *suggesting* its potential for the treatment of urinary tract infections [51]. In the study of Alburqan et al., compounds isolated from the MeOH extract of *S. hortensis* showed additive effects with antibiotics against pathogenic bacteria. For example, naringenin (**42**) and rosmarinic acid methyl ester improved ciprofloxacin activity against *Klebsiella pneumoniae* (eightfold), while combinations with ampicillin and gentamicin produced fourfold MIC reductions against *Staphylococcus aureus* and *Bacillus subtilis*, respectively [41]. Recent studies on nanoliposomal or nanocomposite formulations of EO of *S. hortensis* and *S. khuzestanica* report enhanced antibacterial activity and genoprotective effects against DNA damage [101,108,109].

### 4.2. Antifungal Activity

The antifungal properties of *S. kermanica*, *S. thymbra*, *S. hortensis*, *S. montana*, *S. khuzistanica*, *and S. cilicica* were investigated in vitro. The results show that both EOs and extracts are effective against plant and human pathogenic fungal species, making them promising agents against fungal infections after successful in vivo, clinical, or field studies.

EOs of *S. montana* showed strong activity against 30 strains of *Candida albicans* [7], with MIC values ranging from 0.0019% to 1% (*v*/*v*), and were more effective than clotrimazole. Similarly, the antifungal efficacy of *S. khuzistanica* EO nanoemulsions against cucumber powdery mildew was demonstrated, with carvacrol (88.6%) identified as the main constituent [110], indicating a likely role of phenolic monoterpenes in the observed activity. These antifungal effects are primarily associated with bioactive compounds, such as carvacrol (**5**) and thymol (**7**), which disrupt membrane integrity, increase permeability, and affect ergosterol-related functions, while synergistic constituents including *p*-cymene (**6**) and *γ-*terpinene (**8**) can enhance membrane penetration and overall antifungal efficacy.

Two studies reported notable antifungal activity of *Satureja* species EOs, suggesting their potential use as natural preservatives in the food industry. According to Sasanian et al., notable antifungal activity was observed when the antifungal properties of EO derived from *S. hortensis* were evaluated against *Aspergillus fumigatus* both in vitro and in a food model system [38]. In another study, pure *S. thymbra* EO showed high antifungal activity against different foodborne pathogens; the MIC ranged from 0.0002 to 0.0080 mg of EO/mL and the MFC ranged from 0.0003 to 0.0080 mg/mL against strains of *A. fumigatus*, *A. niger*, *T. viride*, *P. verrucosum*, *C. albicans*, and *C. krusei*, which were lower than those of the antifungal drug ketoconazole [111]. Furthermore, *S. cilicica* EO showed strong antifungal activity against *Sclerotinia sclerotiorum* [112]. In another study, *S. montana* EO showed strong antifungal activity against both growing and stationary-phase cells of *C. albicans* and effectively inhibited hyphae formation. In combination with amphotericin B, it demonstrated improved efficacy against stationary phase cells compared to single treatments [102].

The recently identified Skh-AMP1 peptide exhibited strong antifungal activity against pathogenic *Aspergillus fumigatus*, *Candida glabrata* and *Candida krusei*, with MIC values of 19.8–23.4 μM and MFC values of 39.6–58.5 μM. The peptide also showed favorable stability under physiological conditions together with negligible cytotoxic and hemolytic activity, highlighting its potential as a promising antifungal therapeutic candidate, although more in vivo studies are still needed [99].

### 4.3. Antiparasitic, Anthelmintic, and Antiprotozoal Activities

Four species of *the Satureja* genus have been studied for their antiparasitic, anthelmintic, and antiprotozoal activities, including *S. hortensis*, *S. khuzistanica*, *S. montana*, and *S. thymbra*, demonstrating broad-spectrum efficacy against protozoa and helminths (Table S1).

In the study by Băies et al., the EtOH extract of *S. hortensis* both destroyed and inhibited the development of oocysts of *Eimeria* spp. isolated from infected piglets at all concentrations tested, which supports their use as anticoccidial disinfectants in livestock facilities [113].

Similarly, Jahanshahi et al. demonstrated that *S. khuzistanica* EO modulated MDR1 gene expression in *Leishmania* promastigotes, with optimal activity observed at 20 µg/mL, suggesting possible interference with parasite drug resistance mechanisms [114]. The authors have associated the antiparasitic and anthelmintic activities of *Satureja* species with bioactive terpenoids capable of interfering with parasite enzymatic systems, membrane stability, and neuromuscular functions.

Anisakiasis is a zoonotic infection caused by ingesting live *Anisakis simplex* L3 larvae through raw or undercooked fish. EOs from two varieties of *S. montana* (subsp. *montana* and subsp. *variegata*) completely inactivated *A. simplex* larvae within 24 h in vitro, reduced their penetration ability, and inhibited acetylcholinesterase (AChE). Their nematicidal effect is likely related to inhibition of AChE, which disrupts acetylcholine breakdown and leads to paralysis and death of the parasite [115].

Winter savory EO was found to be effective against gastrointestinal nematodes in sheep. Anthelmintic activity was determined using in vitro egg hatch tests and in vivo fecal egg count reduction tests, along with toxicity and coproculture analyzes. Its anthelmintic potential, together with the absence of adverse effects in sheep, suggests that *S. montana* EO is suitable for controlling sheep nematodes as part of an integrated parasite management strategy. This activity was associated with an EO rich in *p*-cymene (42.8%), highlighting the role of synergistic terpene interactions in anthelmintic efficacy [116]. Furthermore, *S. hortensis* EO was evaluated by Štrbac et al. in vitro and in vivo for its anthelmintic potential using the same nematode models [43]. Khalil et al. found that Libyan *S. thymbra* EO exhibited high anthelmintic activity, exceeding that of standard piperazine citrate against earthworms and reported that the altitude of the plant collection significantly influenced the yield, composition and biological activities of plant extracts [72]. Moreover, Băies et al. investigated the anthelmintic activity of *S. hortensis* and reported that the alcoholic extract of the plant had strong anthelmintic activity against *Ascaris suum*, a common parasitic nematode affecting pigs [113]. Their results indicated that *S. hortensis* can potentially be a prospective source for developing new anthelmintic herbal remedies. Furthermore, Mahmoudvand et al. highlighted the in vitro antitrichomonas activity of the MeOH extract of *S. khuzistanica* against clinical isolates of *Trichomonas vaginalis* [117].

### 4.4. Antioxidant Activity

The antioxidant activity of *Satureja* species has been widely studied. Table S1 shows that between 2014 and 2025, 28 species of *Satureja* were investigated, and data were published in 54 articles. Most investigations used a combination of two or more methods to assess antioxidant potency. Among the different assays, 2,2-diphenyl-1-picryl-hydrazyl-hydrate (DPPH) and 2,2′-azinobis(3-ethylbenzothiazoline-6-sulfonic acid) (ABTS) were used mainly to determine the free radical scavenging potential. EO and extracts of different polarities were found to have radical scavenging, metal chelating, and power-reducing activities, and total antioxidant capacity. High antioxidant activity was demonstrated for the EOs of *S. hortensis*, *S. barceloi*, *S. thymbrifolia*, *S. bachtiarica* and *S. montana* [22,29,63,73,83] because of the presence of oxygenated monoterpenes, particularly thymol (**7**) and carvacrol (**5**). However, alcoholic and aqueous extracts containing polar compounds were also found to have radical scavenging activity, as shown in Table S1. In these extracts, flavonoids, phenolic acids, and their derivatives were responsible for the high antioxidant potency. However, all reported antioxidant activity is currently supported only by in vitro evidence; therefore, more in vivo and clinical studies are required in the future to confirm its efficacy in humans.

### 4.5. Protection Against Heavy Metal Damage

Aboubaker et al. reported the protective effects of *S. hortensis* EO against lead acetate-induced toxicity in rats. Exposure to lead causes marked oxidative stress, inflammation, and functional impairment in vital organs, including the brain, liver, kidneys, and heart. Treatment with EO of *S. hortensis* significantly improved biochemical, behavioral, and electrophysiological parameters, while reducing oxidative damage and inflammatory markers such as MDA and TNF-α. Histopathological analyses confirmed a reduced in tissue injury, particularly at higher doses of EO. These effects are likely attributable to phenolic compounds in EO, such as carvacrol (**5**). The present findings highlight the antioxidant and anti-inflammatory potential of *S. hortensis* EO as a natural protective agent against heavy metal toxicity [118].

### 4.6. Antiglycation Effect

Advanced glycation end products (AGEs) are compounds formed during the Maillard reaction and are involved in the development of various diseases related to oxidative stress, including diabetic complications, cardiovascular disorders, and neurodegenerative conditions. The study by Rahimmalek et al. compared the antioxidant and antiglycation properties of the 80% MeOH extracts of seven Iranian endemic species of the Lamiaceae family, including *S. hortensis*, *S. bachtiarica* and *S. sahendica*, using the Congo red binding assay. Among the tested species, *S. hortensis* showed the most pronounced antiglycation effect, which was mainly associated with its rich content of rosmarinic acid and other phenolic compounds [89].

### 4.7. Antitumor Activity

According to our literature survey (Table S1), several researchers have evaluated the antitumor activities of 16 *Satureja species*, including *S. hortensis*, *S. intermedia*, *S. khuzestanica*, *S. montana*, *S. parnassica*, *S. cuneifolia*, *S. horvatii*, *S. subspicata*, *S. rechingeri*, *S. bachtiarica*, *S. thymbra*, *S. isophylla, S. thymbrifolia* and *S. kermanica*. Several cell lines, including MCF7, A549, HepG2, Hep3B, HCT-116, HaCaT, Panc-1, K562, HT-29, CaCo-2 and HEK293, were used in the in vitro investigations to assess the cytotoxic, antiproliferative, apoptosis-inducing or genotoxic activities of EO and/or extracts of these species.

The hexane and dichloromethane fractions obtained from *S. hortensis* demonstrated potent antileukemic effects against K562 and Jurkat leukaemia cells by inducing apoptosis, altering the cell cycle, and increasing caspase-3 activity, with half maximum inhibitory concentration (IC_50_) values of 32.1–47.8 μg/mL (K562) and 44.3–45.7 μg/mL (Jurkat) [119]. These findings suggest the potential therapeutic utility of these fractions in leukemia treatment, which warrants further research.

The antiproliferative activity of *S. montana* extracts obtained by supercritical carbon dioxide (SC extracts, containing nonpolar compounds) and solid–liquid extraction followed by spray drying (SD, containing polar compounds) was investigated by Vladic et al. using the in vivo model of Ehrlich ascites carcinoma (EAC) in mice. Extracts applied as pretreatment and treatment induced oxidative stress in malignant cells, as evidenced by increased xanthine oxidase activity, decreased catalase activity, and enhanced lipid peroxidation, indicating increased reactive oxygen species production in EAC cells. The SC extracts showed a stronger effect than SD, likely due to their higher carvacrol (**5**) content (SC: 60.82 g/100 g; SD: 902.52 mg/100 g). When applied as treatment, SC extracts reduced the volume of ascites (not significantly) and decreased the number of malignant cells, while pretreatment did not have such an effect, indicating that the extracts were not cytotoxic or cytostatic under the tested conditions. This finding indicated that the extracts may have potential antiproliferative effects on Ehrlich ascites carcinoma and could be explored for potential therapeutic applications [120].

Akara et al. evaluated the apoptotic and genotoxic properties of the *S. subspicata* and *S. horvatii* extracts in mouse and human lymphocyte cultures. The extracts showed a significant reduction in the frequency of reticulocyte micronuclei in mice treated at dosages of 200 mg/kg of the extracts. *S. horvatii* showed antiapoptotic action at doses of 0.2 mg/mL by upregulating anti-apoptotic genes and downregulating pro-apoptotic genes. The authors stated that the high concentrations of phenolic acids in the extracts, especially caffeic and rosmarinic acids (**115**, **137**), were probably responsible for the observed antigenotoxic and antiapoptotic effects [82].

The reports in this section showed that most investigations used crude extracts or EOs. However, the study by Fitsiou et al. was the only study that studied the cytotoxic activity of the main components of the EO of *S. thymbra* and *S. parnassica:* carvacrol (**5**), thymol (**7**), *γ*-terpinene (**8**) and *p*-cymene (**6**). In this investigation, carvacrol (**5**) was the most potent antiproliferative agent against A549 cells (EC_50_ 0.118 ± 0.0012 mΜ), whereas Hep3B cells were more sensitive to thymol (**7**) (EC_50_: 0.181 ± 0.016 mΜ) [67]. Further, *γ*-terpinene (**8**) and *p*-cymene (**6**) had limited bioactivity. Furthermore, fatty acids from *S. hortensis*, *S. rechingeri*, *S. sahendica*, *S. bachtiarica*, *S. khuzestanica* and *S. mutica* reduced the viability of macrophages in a concentration-dependent manner, with cytotoxicity increasing sharply above 0.12 mg/mL in a hematopoietic mouse macrophage cell line, indicating potential antitumor activity [121].

Pavlovic et al. investigated the genoprotective potential of *S. montana* using the pUC19/*Escherichia coli* XL1-Blue SOS assay and the antigenotoxicity assay on *Salmonella* Typhimurium. The methanolic extract exhibited SOS-inducing activity, whereas the ethanolic extract showed pronounced antigenotoxic activity. In contrast, the *S. montana* extracts were inactive against the human colon cancer cell line HCT-116. However, the extracts provided antioxidant protection to acellular, prokaryotic, and normal human DNA, while modulating ROS and NO production in tumor cells, while inducing genotoxic effects in tumor cells. However, the bioactive compounds responsible for these effects were not identified [94].

In the study by Kheiri et al., the extract of *S. khuzistanica* reduced the viability of HT-29 colorectal cancer cells by 50% at 20 μg/mL. Treatment upregulated the expression of pro-apoptotic genes (*BAX*, *SMAC*, *P53*, and *CASP9*) and decreased *BCL2*, *MMP2*, *SUR*, and *MMP9*. Combined treatment with nisin or doxorubicin further enhanced these gene expression changes. In this study only the extract was analyzed; the activity of individual compounds was not reported [122].

### 4.8. Anti-Inflammatory Activity

*S. hortensis*, *S. khuzestanica*, *and S. montana* were investigated for their anti-inflammatory properties during the review period. Dichloromethane, butanol, and hexane extracts of *S. hortensis* seeds were investigated in lipopolysaccharide-activated J774.1 macrophage. The results showed that the dichloromethane and hexane extracts efficiently reduced nitric oxide (NO) production. Both extracts decreased the gene expression of inducible NO synthase reduced to <0.44-fold of the control levels, and inhibited COX-2 <0.29-fold compared to control levels, interleukin (IL)-1β (<0.41 fold), IL-6 (<0.25 fold) and tumor necrosis factor-α (<0.2 fold). The extracts also reduced the production of IL-6 and IL-1β protein in macrophages. These findings suggest that *S. hortensis* exerts anti-inflammatory effects [123].

The activity of EO from *S. khuzistanica* was evaluated against traumatic brain injury (TBI) by Abbasloo et al. [124]. The study explored the neurotherapeutic effects of *S. khuzistanica* EO on diffuse experimental TBI in male Wistar rats. Treatment with EO significantly reduced brain oedema, blood–brain barrier damage, and intracranial pressure increase. It also reduced inflammatory markers and affected astrocytic activation, suggesting its potential clinical neurological applications. The authors suggested that the observed anti-inflammatory effects may be associated with phenolic constituents, particularly thymol (**7**) and carvacrol (**5**), which are known to modulate pro-inflammatory cytokines and inflammatory mediators [121,122].

The anti-inflammatory properties of *S. montana* were documented by Miguel et al. [10]. The EO was toxic to chemokine (C-C motif) ligand 2 in human acute monocytic leukemia cells (THP-1 cells) triggered by lipopolysaccharide at the lowest concentration tested (3 µg/mL). In another study, the dried extract of *S. montana* modulated cytokine levels in male Wistar rats. A 250 mg/kg dose significantly decreased IL-6 in the acute stress model compared to carvacrol and reduced TNF-α and IL-6 compared to rosmarinic acid (**137**). Furthermore, the authors suggested that synergistic interactions between rosmarinic acid (**137**) and carvacrol (**5**) within the extract may contribute to the observed activity [125].

### 4.9. Protective Effects on Side Effects of Chemotherapy

Since 2014, four studies have been conducted to evaluate the protective effects of *S. montana*, *S. hortensis*, and *S. khuzistanica* against chemotherapy-mediated side effects. Nasimi et al. and Abd El Tawab et al. investigated the protective effects of *S. khuzistanica* EO and *S. montana* extracts against busulfan and cyclophosphamide-induced testicular injury in rats [126,127]. In a previous study, preadministration of *S. khuzistanica* EO significantly improved epididymal sperm parameters, decreased germinal epithelium destruction and reduced MTT assay-derived cytotoxicity and TUNEL-positive cells in testicular tissue after busulfan treatment in rats.

In the latter study, daily administration of *S. montana* extract effectively protected the testes from cyclophosphamide-induced damage through antioxidant and anti-apoptotic mechanisms, mediated by up-regulation of peroxisome proliferator activated receptor (PPAR)-*γ* and Akt1 protein levels [127]. These protective effects have been attributed to the rich phytochemical profile of *Satureja* species, particularly flavonoids and phenolic compounds, such as carvacrol (**5**), rutin (**94**), rosmarinic acid and caffeic acid (**115**), which contribute to antioxidant defense, modulation of apoptosis-related pathways, and attenuation of oxidative stress-induced cell damage [78,126,127].

A randomized clinical trial found that a mucoadhesive gel containing 1% *S. hortensis* extract significantly reduced mucositis-induced pain in 60 children undergoing chemotherapy. The gel reduced the severity of the pain from 3.5 ± 2.1 to zero on day 5. This finding indicated that the extract was a promising option for treating mucositis [128]. Another study investigated in vitro the potential of an *S. hortensis* MeOH extract, rich in rosmarinic acid (**137**), against cisplatin-induced oxidative damage in kidney, liver, and testes tissues. The results showed that the extract restored tissue morphology, improved liver, kidney, and testes function, and increased the Bcl-2/Bax ratio [78].

### 4.10. Anti-Diabetic Activity

Anti-diabetic activity within the *Satureja* genus has been mainly associated with enzyme inhibition. An in vitro study showed that *S. macrantha* extract exhibited stronger α-glucosidase inhibition than acarbose, while *S. hortensis* demonstrated moderate α-amylase inhibition mainly attributed to its EO [44]. Similarly, *S. hortensis* and *S. montana* showed notable α-glucosidase and cholinesterase inhibitory activities [129]. Clinical evidence further supports these findings, as *S. khuzestanica* significantly improved glycemic and lipid parameters in patients with type 2 diabetes (T2D) [130]. Furthermore, fatty acids and extracts of various *Satureja* species exhibited anti-lipase and α-amylase inhibitory activities, reinforcing their antidiabetic potential [95,121].

### 4.11. Effects on Fatty Liver Syndrome

Mirderikvandi’s group investigated the effects of *S. khuzistanica* EO and dietary acetic acid on fatty liver syndrome in broiler chickens. Fatty liver syndrome is a metabolic disorder associated with high-energy diets and limited physical activity, conditions common in modern intensive broiler production systems. A total of 252 male Ross 308 broilers were administered different doses of *S. khuzistanica* EO, while some groups were also fed an acidified diet containing acetic acid. The results showed that *S. khuzistanica* EO reduced liver fat accumulation and improved several indicators of liver health, particularly at higher doses (500–600 mg/bird/day), where histological liver damage was also reduced. Acetic acid alone had little effect, although some beneficial interactions between EO and acetic acid were observed. In general, the study suggests that *S. khuzistanica* EO may have hepatoprotective and anti-steatotic effects in broiler chickens, but further studies are warranted to determine the most effective dose [131].

### 4.12. Improve Memory Impairment

Neuroprotective effects within the *Satureja* genus appear to converge on cholinesterase inhibition and protection against oxidative neuronal damage. For example, *S. bachtiarica* demonstrated pronounced in vivo activity by attenuating Aβ-induced memory deficits and reducing lipid peroxidation, indicating direct neuroprotective potential [132]. The authors suggested that these neuroprotective effects may be related to phenolic constituents, particularly rosmarinic acid (**137**), carvacrol (**5**), thymol (7), and other catechol-type compounds, which may contribute to the inhibition of cholinesterase and the attenuation of oxidative neuronal damage [79,85].

In contrast, most other species have been mainly evaluated through enzyme-based assays, where moderate to strong inhibition of AChE and BChE has been recorded. In another study, *S. isophylla* showed strong butyrylcholinesterase inhibition despite weak AChE activity [47]. In particular, *S. hortensis* exhibited stronger cholinesterase inhibition than *S. macrantha*, particularly its EO, while *S. pilosa* showed a relatively potent inhibitory effect against both AChE and BChE [44,79]. Other species, such as *S. aintabensis*, *S. spicigera*, and *S. barceloi*, showed more variable and generally moderate effects, highlighting differences in bioactivity [85,95].

### 4.13. Immunostimulatory Activity

*Aeromonas hydrophila* is one of the most important bacterial pathogens in carp aquaculture, causing significant economic losses, while effective and widely applicable vaccines are still limited due to the high antigenic variability of the species. In this context, natural immunostimulants are increasingly being studied as complementary approaches to improve fish innate immunity and disease resistance. Dietary supplementation with *the* 96% ethanol (EtOH) extract of *S. khuzestanica* was shown to enhance non-specific immune parameters, particularly lysozyme and bactericidal activity in non-vaccinated carp, suggesting a moderate but selective immunostimulatory effect, while no significant improvement was observed in vaccinated fish [133].

### 4.14. Prebiotic Activity

*S. hortensis* EO demonstrated significant prebiotic effects in a humanized mouse model colonized with the gut microbiota derived from patients with ischemic heart disease (IHD) and type 2 diabetes mellitus (T2DM). Supplementation with the EO emulsion promoted the growth of beneficial commensal bacteria, particularly *Lactobacillus* spp. in mice, indicating a favorable modulation of the intestinal microbial composition. Treatment with savory essential oil was also associated with increased levels of thrombomodulin and elevated concentrations of chemokines such as CXCL1, CCL2, and CCL11, suggesting immunomodulatory activity. Furthermore, EO exhibited antioxidant effects, reflected in reduced levels of oxidative stress biomarkers including protein carbonyls and pentosidine. Collectively, these findings suggest that *S. hortensis* EO may contribute to modulation of the gut microbiota and cardiometabolic pathways, highlighting its potential as a nutraceutical in IHD and T2DM [134].

### 4.15. Antispasmodic and Antidiarrheal Activities

According to studies by Kulić et al., *S. montana* EO exhibits significant antispasmodic and antidiarrheal activities, supporting its traditional application in gastrointestinal disorders associated with excessive intestinal motility and secretion. Ex vivo experiments in isolated rat ileum demonstrated that EO induces concentration-dependent smooth muscle relaxation through multiple mechanisms, primarily involving the activation of voltage-gated potassium channels and the voltage-gated calcium channels. These mechanisms decrease smooth muscle excitability and inhibit intestinal contractions, thereby reducing spasm and hypermotility. In vivo studies revealed that *S. montana* EO alleviates castor oil-induced diarrhea and decreases fecal water content, suggesting antisecretory effects mediated by reduced intestinal fluid secretion and/or improved water reabsorption. The observed pharmacological effects are mainly attributed to the high carvacrol (**5**) content of EO, although *β*-caryophyllene (**4**) may also contribute through synergistic interactions. In general, the findings indicate that the complex phytochemical composition of *S. montana* EO enables multimodal modulation of intestinal motility and secretion, highlighting its therapeutic potential in gastrointestinal disorders [4].

### 4.16. Wound Healing Effect

Hydroxypropyl-β-cyclodextrin–glycerol-based extracts of *S. montana* demonstrated remarkable wound healing potential in an *in vitro* scratch assay using HaCaT keratinocyte cells, significantly accelerating wound closure compared to untreated controls. Extracts rich in phenolic acids and flavonoids promoted approximately 48.6% wound closure after 48 h, whereas the control group achieved 34.8%, indicating enhanced keratinocyte migration and tissue regeneration. The formulations also preserved more than 80% cell viability at concentrations of up to 62.5 µL/mL, confirming their high biocompatibility and suitability for direct dermatological application. Their strong antioxidant and anti-inflammatory activities, reflected by potent antioxidant and lipoxygenase inhibitory effects, are likely involved in the acceleration of tissue repair through the reduction in oxidative stress and inflammation. Furthermore, the extracts exhibited UV-A and UV-B absorption properties, highlighting their potential for the development of multifunctional dermatological and cosmeceutical formulations with protective and regenerative effects on the skin [93]. In another study, *S. montana* hydrolate (water-based extracts obtained by steam distillation) significantly reduced oxidative stress and inflammatory markers in burn wounds while restoring antioxidant balance. It also promoted wound healing by decreasing pro-inflammatory cytokines and enhancing anti-inflammatory responses [135].

### 4.17. Other Activities

Ilhan et al. investigated the potential of *S. cuneifolia* extract blended with sodium alginate/polyethylene glycol 3D scaffolds for the treatment of diabetic ulcers [136]. Furthermore, the potential of an *S. khuzistanica* EtOH extract to prevent tolerance to opioid analgesics was investigated in adult male Wistar rats.

The extract (25–50 mg/kg) prevented the development of morphine tolerance in a dose-dependent manner. The authors also found that the extract reversed elevated levels of glial fibrillary acidic protein and tumor necrosis factor (TNF)-α levels in the spinal cord of animals that developed tolerance. This study suggests that the *S. khuzistanica* extract may attenuate morphine-induced analgesic tolerance through its ability to reduce spinal cord glial activation. The authors state that the antioxidant properties of *S. khuzistanica* may, at least in part, be responsible for the antitolerance effect. However, this proposed mechanism requires further investigation and confirmation through additional studies. [137].

Rabiei et al. investigated the antiepileptic effects of *S. bachtiarica* EO in vivo, showing that it caused a significant increase in latency to the first seizure and survival duration, as well as a significant decrease in the frequency of head and forelimb seizures, tonic seizures, and spinning and jumping behaviors in pentylenetetrazole treated mice. Furthermore, flumazenil significantly inhibited these effects, suggesting that anticonvulsant activity is mediated, at least in part, via the GABAergic system. In addition, other mechanisms, such as reduction in oxidative stress, decreased brain acetylcholine levels, and modulation of inflammatory mediators, can also contribute to its anticonvulsant activity; however, these mechanisms were not investigated in this study [138].

The cardioprotective and tissue protective effects of *Satureja* species have been increasingly supported in in vivo studies. For example, *S. hortensis* showed marked cardioprotection in an isoproterenol-induced myocardial infarction model, where a dose of 400 mg/kg significantly (*p* < 0.001) reduced cardiac biomarkers (CK-MB, cTnI, LDH, AST, ALT), improved ECG patterns, and preserved myocardial structure [139].

However, it should be noted that most of the available studies were conducted in vitro and therefore their findings may not fully reflect *the* efficacy, bioavailability, metabolism, safety or clinical relevance in vivo. More well-designed in vivo and clinical studies are required to validate the therapeutic potential of *Satureja* species.

## 5. Innovative Applications of *Satureja* Species

*Satureja* species have been the subject of various innovative developments aimed at the preservation of food quality, improvements in coating materials in the food industry, and the development of new scaffolds for wound dressing and healing. Nasiri et al. investigated the effect of a tragacanth gum coating incorporating different concentrations of *S. khuzistanica* EO on post-harvest quality and shelf life of button mushroom (*Agaricus bisporus*) stored at 4 ± 1 °C for 16 days [140]. The use of a combination of tragacanth gum and EO was found to preserve tissue firmness and sensory quality, as well as decrease microbial counts and ascorbic acid and phenolic component decomposition rates. In addition, compared to the controls, coating with tragacanth gum + EO might preserve the color of the samples. These findings imply that the coating with this combination can function as an edible coating to extend the shelf life of postharvest button mushrooms and is a safe and suitable food additive [140].

Kefiran and carboxymethylcellulose (CMC) are water-soluble biopolymers widely used in food packaging; in addition, kefiran–CMC compositions have improved mechanical and inhibitory properties. Hasheminya et al. developed and characterized biocomposite films incorporating *S. khuzistanica* EO. The results showed that the newly created biocomposite films had increased opacity (to delay lipid oxidation, avoid nutrient degradation, discoloration, and fouling) and antibacterial qualities; these properties make the films suitable for use in food packaging [49].

Azimi et al. examined how the EO of *S. hortensis* affected the development of *Salmonella* Typhimurium in chicken meat [141]. Compared to the control group, the summer savory EO dramatically reduced the *Salmonella* count (*p* < 0.05). The number of *Salmonella* cases was significantly decreased by *S. hortensis* EO as the amount of time that minced beef was stored decreased. However, there was no noticeable increase in *Salmonella* count in the presence of 1% sodium chloride. The findings demonstrated the antibacterial effectiveness of savory EO in summer, making it a useful natural preservative to extend the shelf life of meat and meat products [141].

EOs of *S. rechingeri* and *S. hortensis* inhibited the growth and biofilms of the foodborne pathogen *Listeria monocytogenes*, disrupting preformed biofilms and altering bacterial membranes to increase permeability and induce cell death [69,142]. These results suggest that *Satureja* EO may provide an effective approach to controlling cold-tolerant pathogens such as *L. monocytogenes*, thus improving the shelf life and safety of ready-to-eat meat products.

Nanobiocomposite films incorporating *S. sahendica* EO and zinc oxide nanoparticles were developed and characterized. Films showed strong antibacterial activity against *S. aureus* and *E. coli* and enhanced antifungal performance, supporting their potential use in food preservation applications [70].

Barzegar et al. developed new nanofibrous scaffolds for wound dressings [65]. Core–shell nanofibrous scaffolds composed of chitosan/polyvinyl alcohol as the core and polyvinylpyrrolidone/maltodextrin as the shell were developed in which *S. mutica* or *Oliveria decumbens* EO was encapsulated. The incorporated EOs enhanced the antioxidant activity of the scaffolds and, according to antimicrobial tests, expanded the microbicidal activity of the scaffolds. Because the core–shell nanofibrous scaffolds loaded with EOs have appropriate mechanical characteristics, antioxidant effect, and antibacterial activity, they may be used as dressings for dry-wounds [65].

Another study developed sodium alginate/polyethylene glycol scaffolds incorporating an *S. cuneifolia* extract for the potential treatment of diabetic ulcers [136]. Researchers used new composite scaffolds that were successfully produced using 3D printing technology. Their results showed excellent antibacterial effects, especially on Gram-positive bacteria, and the biocompatibility rates of the scaffolds were at the desired values. The authors propose a promising approach for the treatment of bacterial infections and the healing of diabetic wounds. Evidence shows that 3D printed composite scaffolds with antimicrobial *S. cuneifolia* extract could be a novel and promising approach to tissue engineering and wound dressing applications.

Landfill leachate poses a serious environmental threat, highlighting the need for advanced waste management solutions. In the study of Rezaei, superabsorbent nanocomposites (SANs) incorporating EO of *S. khuzestanica* were developed, providing not only exceptional liquid absorption and mechanical strength but also strong antibacterial activity. The inclusion of *S. khuzestanica* EO was particularly effective against *Escherichia coli* and *Staphylococcus aureus*, demonstrating the potential of these SANs for safer and more efficient leachate treatment [109].

The study of Oliveira-Pinto reported that *S. montana* EO, recognized as GRAS (Generally Regarded As Safe) for use as a biopesticide, has dual functions: foliar applications and nanoformulations exhibit antibacterial activity and can modulate plant hormone responses in tomato plants infected with *Xanthomonas*. Additionally, it acts as a host defense elicitor by altering caffeic acid levels, which may be associated with plant defense responses [143].

The study by Baghouz et al. aimed to identify environmentally friendly alternatives to synthetic pesticides for stored seed pests by evaluating the insecticidal activity of *S. calamintha* EO against *Callosobruchus maculatus* (cowpea seed beetle). The EO showed 100% male mortality, 86.66  ±  23.09% female mortality (LC_50_ 2.17 μL L^−1^), and strong repellency (91.67%), highlighting its potential as a fumigant and repellent agent [26]. Similarly, insecticidal activity of *S. viminea* EO was reported against *Tribolium castaneum* (red flour beetle) [75]. The insecticidal effect (contact and fumigant toxicity) of the EO is related to the high content of pulegone (**9**).

## 6. Discussion

**Figure 6:**
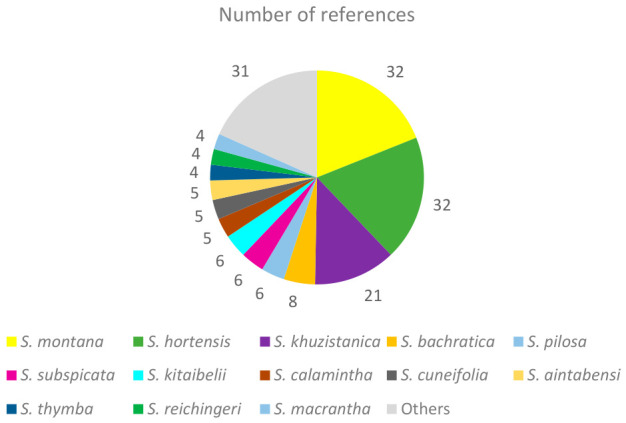
Number of references for each *Satureja* species published between 2014 and 2025. (Other species include: *S. avromanica*, *S. barceloi*, *S. biflora*, *S. boissieri*, *S. candidissima*, *S. cilicica*, *S. coerulea*, *S. hasturkii*, *S. horvatii*, *S. intermedia*, *S. isophylla*, *S. kermanica*, *S. laxiflora*, *S. mutica*, *S. nabateorum*, *S. parnassica*, *S. spicigera*, *S. sahendica*, *S. thymbifolia*, and *S. viminea* with <4 references).

This review summarizes the current knowledge of traditional uses, secondary metabolites, pharmacology, and non-medicinal applications of the genus *Satureja*. During the studied period, *S. hortensis*, *S. montana*, and *S. khuzistanica* were the most extensively studied species, with 32, 32, and 21 publications, respectively (Figure 6).

Ethnobotanical studies published between 2014 and 2025 revealed the use of several *Satureja* species among local communities in Europe and Iran. Most traditional applications are related to the respiratory tract (cough, bronchitis, flu) and the gastrointestinal system (gastric pain, flatulence, bloating, vomiting). One report described a specific usage of *S. montana* subsp. *macedonia*, which involved the treatment of tinnitus and improving hearing ability. This application is unusual and has not been previously reported [17].

The phytochemistry of *Satureja* species has been intensively studied, and EOs, flavonoids, phenolic acids, jasmonates, di- and triterpenes, and steroids have been identified in various studies as the main groups of compounds in the genus. The most studied group is EOs, which are highly variable and constitute substantial components of all *Satureja* species. Extreme chemical diversity was observed in *S. montana* EO. Thymol (**7**), *p*-thymol (**37**), carvacrol (**5**), borneol (**17**), *p*-cymene (**6**) and *α*-pinene (**21**) were found to be the main components of winter savory EO, and enormous variability was also observed in the side components (Table 1). In contrast, the EO profiles of *S. hortensis* were more uniform. The main constituents of EO were carvacrol (**5**) and *γ*-terpinene (**8**) at similar concentrations or thymol (**7**) (Table 2). The variation in the predominant constituents indicates the existence of distinct chemotypes. The variability in the EO profiles of *Satureja* species, including inter- and intra-population variabilities, has been widely studied and shown to be related to the effects of plant organs, geographic origin, domestication, extraction methodology, and vegetation period.

To identify non-volatile compounds (Table 3, Table 4 and Table 5), UHPLC–DAD–(+/−)HRESI-MS/MS or HPLC-MS methods without prior isolation of the compounds have mainly been used. Therefore, in some cases these nonvolatile compounds were only partially characterized, and the quality and linkage of the sugars and ester moieties were not determined. In the last decade, only one new compound, saturejin (**72**), was isolated from the *Satureja* genus, which is an adduct of luteolin and 2,5-dihydroxy-*p*-cymene [80]. Furthermore, the antifungal peptide Skh-AMP1 was isolated for the first time from *S. khuzistanica* leaves [99]. Hydroxylated, methoxylated and glycosylated flavanones, flavanonols, flavones, flavonols and catechins were the main classes of flavonoids identified (Table 3). *Satureja* species accumulate rarely occurring hydroxycinnamic acid oligomers (**105**–**144**) in addition to common phenolic acid. These compounds have complex structures of di-, tri-, tetra-, hexa- and octameric caffeic acid derivatives. A few jasmonates, triterpenes, and sterols were identified from the *Satureja* genus, with rosmanol (**149**) and carnosic acid (**150**) being the only diterpenes identified during the study period.

According to our findings, most of the data in the literature focused on the bioactivities of *Satureja* species and some new bioactivities and applications were discovered (Table S1). Antimicrobial effects have been researched for a long time, and many results on the antibacterial, antifungal, antiparasitic, and antiprotozoal activities of EOs and extracts have been published in the past decade. The investigations were conducted against human pathogenic and foodborne bacteria, as well as human and veterinary parasites. The enhanced potency of combinations of EOs and isolated compounds with conventional antibiotics has been demonstrated in several studies [36,107]. New formulations, such as nanoparticles, nanofibers, and biocomposite films, have been tested for their ability to improve antimicrobial potency [101,108,109]. The antioxidant activity of *Saturea* species has also been well studied. EOs and extracts of different polarities had radical scavenging, metal chelating, and reducing power activity. Studies on the anticancer potential of the *Satureja* genus include measurements of cytotoxic, antiproliferative, antiapoptosis, and genoprotective effects and cell cycle analysis. In addition, the extract of *S. montana* was evaluated in an in vivo model of Ehrlich ascites carcinoma in mice, which demonstrated induced oxidative stress in malignant cells [122]. Several authors suggested that the observed cytotoxic and antiproliferative activities may be associated with the phenolic monoterpenes and phenolic acids present in *Satureja* species, particularly carvacrol (**5**), thymol (**7**), rosmarinic acid (**137**) and caffeic acid (**115**), which are known to modulate oxidative stress, apoptosis and cell cycle regulation [67,82]. Studies conducted to evaluate the protective effects of *Satureja* species against chemotherapy-mediated side effects are very promising. Such protective effects have been confirmed for *S. montana*, *S. hortensis*, and *S. khuzistanica* in four studies [78,126,127,128]. The effects of *Satureja* species on the nervous system are also of great interest. These activities, including antiepileptic and analgesic effects, improvement of memory impairment, and prevention of opioid analgesic tolerance, suggest new directions for *Satureja* research.

Clinical studies on *Satureja* species provide promising evidence for their therapeutic potential. In patients with type 2 diabetes, *S. khuzestanica* significantly improved glycemic control and lipid profile [130], while a randomized clinical trial demonstrated that a mucoadhesive gel containing 1% *S. hortensis* extract markedly reduced chemotherapy-induced mucositis pain in children [128]. Additional clinical evidence indicates the protective effects of *S. hortensis* extracts against cisplatin-induced oxidative damage, supporting the potential clinical relevance of these species.

## 7. Conclusions

Overall, this review provides an overview of the traditional uses and studies on secondary metabolites, pharmacology, and clinical trials of the *Satureja* genus over the last decade. Although the therapeutic potential of *Satureja* species is well documented and supported by in vitro and in vivo studies, the pharmacological activities of individual pure compounds remain largely unexplored. Future research should focus on evaluating individual compounds and their molecular mechanisms and structure–activity relationships. New formulations and modern extraction technologies represent important research directions and support the future use of savory products in the pharmaceutical and food industries.

## Figures and Tables

**Table 1:** Number of publications related to different topics.

Subject	Sub-Group	No. of Publications *
**Ethnobotany**		4
**Phytochemistry**	Essential oils	55
	Flavonoids	26
	Phenolic acids	28
	Jasmonates	4
	Di-, triterpenoids, steroids	6
	Other compounds	9
**Pharmacology**	Antibacterial activity	64
	Antifungal activity	14
	Antiparasitic, anthelmintic and antiprotozoal activities	11
	Antioxidant activity	54
	Anti-tumor activity	25
	Anti-inflammatory activity	5
	Protective effects on chemotherapy side effects	4
	Anti-diabetic activity	6
	Improve memory impairment	5
	Other activities	14
**Innovative applications**		12

* Publications listed in Table S1 were also included.

**Table 2:** Composition of EO of *S. hortensis*.

Plant Material	Main Compounds of EO	Comment	Ref.
Dried aerial parts from cultivation in Serbia	carvacrol (**5**) (46.7%), *γ*-terpinene (**8**) (32.5%), *α*-terpinene (**23**) (4.22%), *p*-cymene (**6**) (3.63%)		[35]
Air-dried aerial parts collected in Iran in early spring	thymol (**7**) (41.28%), *γ*-terpinene (**8**) (37.3%), *p*-cymene (**6**) (12.2%), and *α*-terpinene (**23**) (3.59%)		[38]
Dried aerial parts at full flowering stage collected in Ardebil province, Iran	*γ*-terpinene (**8**) (37.60%), carvacrol (**5**) (32.07%), *p*-cymene (**6**) (13.07%), *α*-terpinene (**23**) (4.63%), *β*-bisabolene (**30**) (3.09%)	EO yield 1.8%	[23]
Dried aerial parts collected at the beginning of the flowering stage in Fars province, Iran	thymol (**7**) (28.5%), *p*-cymene (**6**) (18.9%), *γ*-terpinene (**8**) (16.2%), carvacrol (**5**) (11.0%)		[39]
EO purchased from Oshadhi Ltd. (Cambrigre, UK)	carvacrol (**5**) (39.84%), γ-terpinene (**8**) (34.63%), *p*-cymene (**6**) (10.72%), *α*-terpinene (**23**) (3.51%), and *β*-caryophyllene (**4**) (2.40%)		[40]
20 different accessions originating from Iran, Bulgaria, Germany, Czech, Georgia, Syria, Hungary, Poland, Italy, Greece, and Uzbekistan	γ-terpinene (**8**) (max. 84.03%), carvacrol (**5**) (max. 28.07%), α-terpinene (**23**) (max. 21.4%) and thymol (**7**) (max. 41.13%)	The EO content ranged between 0.1 and 0.75%. The presence of different chemotypes can be proposed.	[41]
EO extracted by SPME, vegetation phases were compared	carvacrol (**5**) predominated and further main compounds were myrcene (**25**), p-cymene (**6**), γ-terpinene (**8**), and *β*-caryophyllene (**4**) in all vegetation phases	The amount of carvacrol (**5**) ranged from 47% in intensive growth to 87% at the end of flowering. The maximum concentration of γ-terpinene (**8**) was reached during intensive growth but decreased to 6% at massive flowering and at the end of the flowering stage.	[36]
Air-dried above ground parts collected from Kerman province, Iran	carvacrol (**5**) (50.68%), *γ*-terpinene (**8**) (34.44%), *α*-terpinene (**23**) (3.72%)	EO yield in the stage of mass flowering was 2.05%.	[42]
EO purchased from Bio Salas Farago, Serbia	carvacrol (**5**) (49.5%), *γ*-terpinene (**8**) (29.7%), *p*-cymene (**6**) (12.9%), *α*-terpinene (**23**) (2.00%)		[43]
Air-dried flowering aerial parts collected in Sülünkaya village, Erzurum Province, Türkiye	thymol (**7**) (45.1%), γ-terpinene (**8**) (29.9%), *p*-cymene (**6**) (8.2%), α-terpinene (**23**) (3.3%), and carvacrol (**5**) (3.2%)	EO yield by hydrodistillation was 0.1%	[44]

**Table 3:** Composition of EO of *S. montana* and its varieties.

**Plant Material**	**EO Yield**	**Main Compounds of EO**	**Conclusion**	**Ref.**
Aerial part of *S. montana* in the flowering stage of the Kherson region (Ukraine)	n.d.	*p*-thymol (**37**) (81.79%), linalool (**32**) (2.09%)	*p*-Thymol (**37**), the isomer of thymol (**7**) and carvacrol (**5**) are the dominant compounds	[55]
*S. montana* EO from a commercial source	-	carvacrol (**5**) (43.9%), thymol (**7**) (7.6%), thymol methyl ether (**39**) (4.3%), borneol (**17**) (3.1%), caryophyllene (**4**) (3.4%)	-	[59]
Leaves of *S. montana*, Belo Horizonte, Brazil	n.d.	borneol (**17**) (36.18%), *γ*-terpinene (**8**) (12.66%), carvacrol (**5**) (11.07%) and *p-*cymene (**6**) (9.57%)	Borneol (**17**) as a major compound of *S. montata* EO is unprecedented.	[56]
Fresh flowering aerial parts of *S. montana* subsp. *variegata* from cultivation in Northern Italy	1.1%	carvacrol (**5**) (22.5%), *p*-cymene (**6**) (17.6%), thymol (**7**) (17.4%), *γ*-terpinene (**8**) (9.1%) and carvacrol methyl ether (**40**) (7.1%)	The main difference between the two subspecies was the carvacrol (**5**)–thymol (**7**) ratio, above 300 (subsp. *montana*) and about 1 (subsp. *variegata*).	[57]
Fresh flowering aerial parts of *S. montana* subsp. *montana* from cultivation in Northern Italy	1.5%	carvacrol (**5**) (61.9%), *p*-cymene (**6**) (9.9%) and *γ*-terpinene (**8**) (8.2%)		
Aerial parts of *S. montana* subsp. *montana* collected in two population in Italy	0.9% both population	coast population: *α*-pinene (**21**) (26.96%), *α*-terpineol (**22**) (15.70%), *trans*-*β*-ocimene (**38**) (11.45%), linalool (**32**) (7.37%), D-limonene (**35**) (7.05%); Interland population: thymol (**7**) (46.10%), *γ*-terpinene (**8**) (14.57%), *p*-cymene (**6**) (10.43%)	The EO profile of the coast and inland populations is very different.	[34]
Commercial sample of *S. montana* EO from Bosnia and Herzegovina	-	thymol (**7**) (44.6%), *p*-cymene (**6**) (13.4%), carvacrol (**5**) (6.2%), *γ*-terpinene (**8**) (4.7%)	-	[60]
Herbs of *S. montana* in the initial flowering phase of cultivation in Ljubinje Bosnia and Hercegovina	1.45%	carvacrol (**5**) (54.9%), *γ*-terpinene (**8**) (14.5%), *p*-cymene (**6**) (8.8%), *β*-caryophyllene (**4**) (3.2%)		[4]
Commercial sample of *S. montana* EO from Niš, Serbia	n.d.	predominant compounds were carvacrol (**5**) (24.3%), thymol (**7**) (15.5%) and *p*-cymene (**6**) (12.2%)		[61]
*S. montana* EO obtained from dried cultivated plant (Ni, Serbia) by steam distillation	n.d.	*p*-cymene (**6**) (42.8%), carvacrol (**5**) (28.11%), and *γ*-terpinene (**8**) (14.59%)		[62]
EO from *S. montana* dried herbs from Bego (B) and Dajti (D) Mountain, Albania	0.39% and 0.73%	B: thymol (**7**) (52.8%), and *p*-cymene (**6**) (8.9%) D: thymol (**7**) (28.5%), and *p*-cymene (**6**) (11.8%)	Both samples belong to the thymol chemotype.	[63]

n.d. with no data.

**Table 5:** Phenolic acids in the genus *Satureja*.

**Compound Name**	**Plant Species**	**Reference**
**Benzoic acid derivatives**		
4-Hydroxybenzoic acid (**105**)	*S. horvatii*, *S. subspicata*, *S. hortensis*, *S. montana*, *S. barceloi*	[82,86,88,90,95]
Gentisic acid (**106**)	*S. montana*	[96]
Protocatechuic acid (**107**)	*S. montana*	[96]
Gallic acid (**108**)	*S. montana*	[86]
Salicylic acid (**109**)	*S. kitaibelii*, *S. horvatii*, *S. subspicata*, *S. aintabensis*, *S. spicigera*	[76,82,85]
Syringic acid (**110**)	*S. montana*, *S. hortensis*, *S. bachtiarica*, *S. sahendica*, *S. horvatii*, *S. subspicata*, *S. macrantha; S. aintabensis*, *S. spicigera*, *S. barceloi*	[8,44,71,78,82,85,88,89,95,96]
Vanillic acid (**111**)	*S. montana*, *S. hortensis*, *S. horvatii*, *S. subspicata*, *S. macrantha*, *S. aintabensis*, *S. spicigera*, *S. pilosa*	[44,79,82,85,86,88,96]
Ellagic acid (**112**)	*S. subspicata*, *S. montana*, *S. pilosa*	[8,71,79]
**Cinnamic acid derivatives**		
Cinnamic acid (**113**)	*S. horvatii*, *S. subspicata*	[82]
p-Coumaric acid (**114**)	*S. subspicata*, *S. montana*, *S. hortensis*, *S. kitaibelii*, *S. boissieri*, *S. bachtiarica*, *S. sahendica*, *S. horvatii*, *S. hasturkii*	[8,71,77,78,82,84,89,91,96]
Caffeic acid (**115**)	*S. subspicata*, *S. montana*, *S. hortensis*, *S. kitaibelii*, *S. bachtiarica*, *S. sahendica*, *S. horvatii*, *S. boissieri*, *S. macrantha; S. aintabensis*, *S. spicigera*, *S. hasturkii. S. pilosa*	[8,44,55,71,77,78,79,82,84,85,86,88,89,91,94,96]
Ferulic acid (**116**)	*S. subspicata*, *S. montana*, *S. hortensis*, *S. bachtiarica*, *S. sahendica*, *S. horvatii*, *S. macrantha*	[44,71,82,88,89,96]
Sinapic acid (**117**)	*S. hortensis*	[78,88]
5-Hydroxyferulic acid (**118**)	*S. horvatii*, *S. subspicata*	[82]
Caffeic acid ethyl ester (**119**)	*S. bachtiarica*	[87]
(6-*O*-Caffeoyl)-β-d-glucoside (**120**)	*S. bachtiarica*	[87]
Chlorogenic acid (**121**)	*S. montana*, *S. kitaibelii*, *S. montana*, *S. hortensis*, *S. horvatii*, *S. subspicata*, *S. macrantha; S. aintabensis*, *S. spicigera*, *S. pilosa*	[44,55,71,77,78,79,82,85,86,88,90,94]
3,5-Dicaffeoylquinic acid (**122**)	*S. kitaibelii*	[77]
Cynarin (**123**)	*S. kitaibelii*	[77],
Salvianolic acid A (**124**)	*S. kitaibelii*	[76]
Salvianolic acid B (**125**)	*S. cuneifolia*, *S. montana*	[33,93]
Salvianolic acid E (**126**)	*S. kitaibelii*	[77]
Salvianolic acid L (**127**)	*S. kitaibelii*	[77]
Salvianolic acid K (**128**)	*S. kitaibelii*	[77]
Salvianolic acid P (**129**)	*S. pilosa*	[97]
Isosalvianolic acid A (**130**)	*S. cuneifolia*	[33]
Clinopodic acid I (**131**)	*S. kitaibelii*, *S. biflora*, *S. pilosa*	[76,77,81,97]
Clinopodic acid K (**132**)	*S. kitaibelii*, *S. biflora*	[76,77]
Clinopodic acid O (**133**)	*S. kitaibelii*, *S. biflora*, *S. pilosa*	[76,77,81,97]
Clinopodic acid P (**134**)	*S. biflora*	[77]
Melitric acid A (**135**)	*S. biflora*	[77]
Methyl melitric acid A (**136**)	*S. biflora*	[77]
Rosmarinic acid (**137**)	*S. subspicata*, *S. montana*, *S. avromanica*, *S. kitaibelii*, *S. hortensis*, *S. cuneifolia*, *S. boissieri*, *S. biflora*, *S. bachtiarica*, *S. sahendica*, *S. horvatii*, *S. macrantha; S. aintabensis*, *S. spicigera*, *S. hasturkii. S. pilosa*	[8,21,31,33,36,44,55,71,76,77,78,79,82,84,85,86,87,88,89,90,91,93,94,98],
Rosmarinic acid methyl ester (**138**)	*S. hortensis*	[41]
Rosmarinic acid glucuronide *	*S. cuneifolia*	[33]
Rosmarinic acid hexoside *	*S. kitaibelii*	[76]
Chicoric acid (**139**)	*S. pilosa*	[79]
Sagerinic acid (**140**)	*S. barceloi*	[95]
Sagecoumarin (**141**)	*S. montana*, *S. bachtiarica*	[87,93]
Verbascoside (**142**)	*S. spicigera*	[85]
**Other acids**		
2,3-dihydroxyphenylacetic acid (**143**)	*S. montana*	[96]
3,4-dihydroxyphenylacetic acid (**144**)	*S. montana*	[96]

* Structures were not fully determined.

**Table 6:** Jasmonates, di-, triterpenes, and steroids of *Satureja* species.

**Jasmonates**	**Plant**	**Reference**
(Z)-Jasmone (**145**)	*S. calamintha* spp. *nepeta*	[27]
12-Hydroxyjasmonic acid (**146**)	*S. bachtiarica*	[87]
12-*O*-Hexosyljasmonate *	*S. kitaibelii*	[76]
12-Hydroxyjasmonic acid 12-*O*-hexoside *	*S. kitaibelii*	[77]
12-*O*-(Caffeoylhexosyl)-jasmonate	*S. kitaibelii*	[76,77]
12-*O*-(Methylcaffeoyl)hexosyl-jasmonate *	*S. kitaibelii*	[76]
Tuberonic acid 12-*O*-[6’-*O*-(*E*)-feruloyl]-*β*-d-glucopyranoside (**147**)	*S. bachtiarica*	[87]
12-Hydroxyjasmonic acid-(6’-*O*-caffeoyl)-glucoside (**148**)	*S. bachtiarica*	[87]
**Diterpenes, triterpenes, steroids**		
Rosmanol (**149**)	*S. kitaibelii*	[76,77]
Carnosic acid (**150**)	*S. barceloi*	[95]
Ursolic acid (**151**)	*S. montana*	[92]
Oleanolic acid (**152**)	*S. bachtiarica*	[87,92]
Ergosterol (**153**)	*S. hortensis*	[92]
*β*-Sitosterol (**154**)	*S. hortensis*	[92]
(3*β*,22*E*)-Ergosta-5,22-dien 3-acetate (**155**)	*S. hortensis*	[11]
11*α*-Hydroxyandrosta-1,4-diene-3,17-dione (**156**)	*S. hortensis*	[11]
3-Oxo-20-methyl-11α-hydroxyconanine-1,4-diene (**157**)	*S. hortensis*	[11]

* The structure was not fully determined.

## Data Availability

No new data was created or analyzed in this study. Data sharing is not applicable to this article.

## Supplementary Materials

The following supporting information can be downloaded at: https://www.mdpi.com/article/10.3390/ph19060875/s1. Table S1. Biological activities of *Satureja* species as reported in the original research articles reviewed. References [144,145,146,147,148,149,150,151,152,153,154,155,156,157,158,159,160,161,162,163,164,165,166,167,168,169,170,171] are cited in the Supplementary Materials.

## Abbreviations

Akt1	RAC (Rho family)-alpha serine/threonine protein kinase
Aβ	Amyloid β
CMC	carboxymethylcellulose
COX-2	Cyclooxygenase-2
DPPH	(2,2-Diphenyl-1-picrylhydrazyl)
EO	essential oil
EtOH	ethanol
GFAP	Glial fibrillary acidic protein
HD	hydrodistillation
IL-1β	Interleukin 1 beta
IL-6	Interleukin 6
LPS	lysosomal polysaccharide
MBC	minimum bactericidal concentration
MeOH	Methanol
MIC	minimum inhibitory concentration
MSHD	microwave-assisted steam hydrodiffusion
MTT	3-[4,5-Dimethylthiazol-2-yl]-2,5 diphenyl tetrazolium bromide
PPAR-γ	Peroxisome proliferator-activated receptor gamma
SPME	solid-phase microextraction
TNF-α	Tumor necrosis factor-alpha
TUNEL	terminal deoxynucleotidyl transferase dUTP nick end labeling

## References

[1] Senatore F., Urrunaga Soria E., Urrunaga Soria R., Della Porta G., De Feo V. (1998). Essential oils from two Peruvian *Satureja* species. Flav. Frag. J..

[2] Tepe B., Cilkiz M. (2016). A pharmacological and phytochemical overview on *Satureja*. Pharm. Biol..

[3] Zavatti M., Zanoli P., Benelli A., Rivasi M., Baraldi C., Baraldi M. (2011). Experimental study on *Satureja montana* as a treatment for premature ejaculation. J. Ethnopharmacol..

[4] Kulić M., Drakul D., Sokolović D., Kordić-Bojinović J., Milovanović S., Blagojević D. (2023). Essential oil of *Satureja montana* L. from Herzegovina: Assessment of composition, antispasmodic, and antidiarrheal effects. Rec. Nat. Prod..

[5] Momtaz S., Abdollahi M. (2010). An update on pharmacology of *Satureja* species; from antioxidant, antimicrobial, antidiabetes and anti-hyperlipidemic to reproductive stimulation. Int. J. Pharmacol..

[6] Zargari A. (1990). Medicinal Plants.

[7] Bona E., Cantamessa S., Pavan M., Novello G., Massa N., Rocchetti A., Berta G., Gamalero E. (2016). Sensitivity of *Candida albicans* to essential oils: Are they an alternative to antifungal agents?. J. Appl. Microbiol..

[8] Kremer D., Kosir I.J., Koncic M.Z., Cerenak A., Potocnik T., Srecec S., Kosalec I. (2015). Antimicrobial and antioxidant properties of *Satureja montana* L. and *S. subspicata* Vis. (Lamiaceae). Curr. Drug. Targets.

[9] Jafri S.A.A., Khalid Z.M., Khan M.R., Ashraf S., Ahmad N., Karami A.M., Rafique E., Ouladsmane M., Al Suliman N.M., Aslam S. (2023). Evaluation of some essential traditional medicinal plants for their potential free scavenging and antioxidant properties. J. King Saud. Univ. Sci..

[10] Miguel M.G., da Silva C.I., Farah L., Castro Braga F., Figueiredo A.C. (2020). Effect of essential oils on the release of TNF-α and CCL2 by LPS-stimulated THP-1 Cells. Plants.

[11] Huwaimel B., Abouzied A.S., Anwar S., Elaasser M.M., Almahmoud S.A., Alshammari B., Alrdaian D., Alshammari R.Q. (2023). Novel landmarks on the journey from natural products to pharmaceutical formulations: Phytochemical, biological, toxicological and computational activities of *Satureja hortensis* L.. Food Chem. Toxicol..

[12] Vosough-Ghanbari S., Rahimi R., Kharabaf S., Zeinali S., Mohammadirad A., Amini S., Yasa N., Salehnia A., Toliat T., Nikfar S. (2010). Effects of *Satureja khuzestanica* on serum glucose, lipids and markers of oxidative stress in patients with type 2 diabetes mellitus: A double-blind randomized controlled trial. Evid. Based Complement. Altern. Med..

[13] Jafari F., Ghavidel F., Zarshenas M.M. (2016). A Critical Overview on the Pharmacological and Clinical Aspects of Popular *Satureja* Species. J. Acupunct. Meridian Stud..

[14] Ejaz A., Waliat S., Arshad M.S., Khalid W., Khalid M.Z., Rasul Suleria H.A., Luca M.I., Mironeasa C., Batariuc A., Ungureanu-Iuga M. (2023). A comprehensive review of summer savory (*Satureja hortensis* L.): Promising ingredient for production of functional foods. Front. Pharmacol..

[15] Fierascu I., Dinu-Pirvu C.E., Fierascu R.C., Velescu B.S., Anuta V., Ortan A., Jinga V. (2018). Phytochemical Profile and Biological Activities of *Satureja hortensis* L.: A Review of the Last Decade. Molecules.

[16] Sefidkon F., Emami Bistgani Z. (2021). Integrative review on ethnobotany, essential oil, phytochemical, agronomy, molecular and pharmacological properties of *Satureja* species. J. Essent. Oil Res..

[17] Tsioutsiou E.E., Giordani P., Hanlidou E., Biagi M., De Feo V., Cornara L. (2019). Ethnobotanical Study of Medicinal Plants Used in Central Macedonia, Greece. Evid. Based Complement. Altern. Med..

[18] Cavalloro V., Robustelli della Cuna F.S., Quai E., Preda S., Bracco F., Martino E., Collina S. (2022). Walking around the Autonomous Province of Trento (Italy): An Ethnobotanical Investigation. Plants.

[19] Nasab F.K., Khosravi A.R. (2014). Ethnobotanical study of medicinal plants of Sirjan in Kerman Province, Iran. J. Ethnopharmacol..

[20] Matejić J.S., Stefanović M., Ivković M., Živanović N., Marin P.D., Džamić A.M. (2020). Traditional uses of autochthonous medicinal and ritual plants and other remedies for health in Eastern and South-Eastern Serbia. J. Ethnopharmacol..

[21] Abdali E., Javadi S., Akhgari M., Hosseini S., Dastan D. (2017). Chemical composition and biological properties of *Satureja avromanica* Maroofi. J. Food. Sci. Technol..

[22] Alizadeh A. (2016). Essential oil constituents and biological activities of different ecotypes of *Satureja bachtiarica* Bunge. as a traditional herbal drug in southwestern, Iran. J. Essent. Oil-Bear. Plants.

[23] Jafari F., Farmani F., Zomorodian K., Moein M., Faridi P., Zarshenas M.M. (2018). A Study on Essential Oil Chemical Compositions, Antioxidant, and Antimicrobial Activities of Native and Endemic *Satureja* Species Growing in Iran. Pharm. Chem. J..

[24] Memarzadeh S.M., Gholami A., Pirbalouti A.G., Masoum S. (2020). Bakhtiari savory (*Satureja bachtiarica* Bunge.) essential oil and its chemical profile, antioxidant activities, and leaf micromorphology under green and conventional extraction techniques. Ind. Crop Prod..

[25] Abbad I., Soulaimani B., Abbad A. (2023). Chemical composition, insecticidal and allelopathic properties of EOs obtained from wild and cultivated Moroccan *Satureja calamintha* (L.). J. Nat. Pestic. Res..

[26] Baghouz A., Bouchelta Y., Es-safi I., El Brahimi R., Imtara H., AlZain M.N., Noman O.M., Shahat A.A., Guemmouh R. (2024). Biocidal activity of *Ziziphora hispanica* L. and *Satureja calamintha Scheele* L. essential oils against the *Callosobruchus maculatus* (Fabricius) pest on cowpea seeds during storage. Front. Sustain. Food Syst..

[27] Bouzidi N., Mederbal K., Bouhadi D. (2018). Chemical composition of the essential oil of *Satureja calamintha* subsp. Nepeta of west Algerian. Mor. J. Chem..

[28] El Brahimi R., El Barnossi A., El Moussaoui A., Chebaibi M., Kachkoul R., Baghouz A., Nafidi H.A., Salamatullah A.M., Bourhia M., Bari A. (2023). Phytochemistry and biological activities of essential oils from *Satureja calamintha* Nepeta. Separations.

[29] Krimat S., Dahmane D., Senani R., Merah S., Ksouri A., Tigrine C., Benyammi R., Alili M., Metidji H., Nouasri A. (2025). Essential oil of Algerian endemic *Satureja candidissima* (Munby): A study of its biological activities. J. Essent. Oil-Bear. Plants.

[30] Saidi A.E., Bouzidi N., Ziane M., Gherib M., Rahila C., Mioc M. (2025). In silico and in vitro studies: Investigating the chemical composition, DFT, molecular docking, and dynamic simulation of *Satureja candidissima* (Munby) Briq essential oil as a potential antibacterial agent. J. Biomol. Struct. Dyn..

[31] Shanaida M., Korablova O., Bakalets D., Potikha N., Rakhmetov D. (2025). Chemotaxonomic Characteristics of *Satureja coerulea* (Lamiaceae family) based on Analysis of its Bioactive Compounds. Biomed. Pharmacol. J..

[32] El Beyrouthy M., Cazier F., Arnold N.A., Aboukais A. (2015). Seasonal variation in yield and composition of essential oil from *Satureja cuneifolia* Ten. growing wild in Lebanon. J. Essent. Oil-Bear. Plants.

[33] Yıldız G., İlgün S., Şeker Karatoprak G., Köse Y.B., Göger F., Temel H.E., Demirci B. (2023). Chemical profile, in vitro pharmacological activity and *Satureja cuneifolia* Ten. evaluation of essential oil based on distillation time. Int. J. Environ. Health Res..

[34] Perrino E.V., Valerio F., Jallali S., Trani A., Mezzapesa G.N. (2021). Ecological and biological properties of *Satureja cuneifolia* Ten. and *Thymus spinulosus* Ten.: Two wild officinal species of conservation concern in Apulia (Italy). A Preliminary survey. Plants.

[35] Lesjak M., Simin N., Orcic D., Franciskovic M., Knezevic P., Beara I., Aleksic V., Svircev E., Buzas K., Mimica-Dukic N. (2016). Binary and tertiary mixtures of *Satureja hortensis* and *Origanum vulgare* essential oils as potent antimicrobial agents against *Helicobacter pylori*. Phytother. Res..

[36] Bimbiraitė-Survilienė K., Stankevičius M., Šuštauskaitė S., Gęgotek A., Maruška A., Skrzydlewska E., Barsteigienė Z., Akuņeca I., Ragažinskienė O., Lukošius A. (2021). Evaluation of chemical composition, radical scavenging and antitumor activities of *Satureja hortensis* L. herb extracts. Antioxididants.

[37] Alburqan M., Kincses A., Paizs M., Barta A., Veres K., Csámpai A., Yazdani M., Hohmann J. (2025). Adjuvant potential of *Satureja hortensis* metabolites with antibiotics against Gram-positive and Gram-negative bacterial strains. Pharm. Biol..

[38] Sasanian N., Sari A.A., Mortazavian A.M. (2018). Effects of *Thymus daenensis* and *Satureja hortensis* L. essential oils on quality properties of Iranian Doogh. J. Food. Saf..

[39] Mafakheri H., Mirghazanfari S.M. (2018). Antifungal activity of the essential oils of some medicinal plants against human and plant fungal pathogens. Cell. Mol. Biol..

[40] Kim J.E., Lee J.E., Huh M.J., Lee S.C., Seo S.M., Kwon J.H., Park I.K. (2019). Fumigant antifungal activity via reactive oxygen species of *Thymus vulgaris* and *Satureja hortensis* essential oils and constituents against *Raffaelea quercus-mongolicae* and *Rhizoctonia solani*. Biomolecules.

[41] Fathi R., Mohebodini M., Chamani E. (2021). Evaluation of genetic diversity of summer savory (*Satureja hortensis* L.) accessions based on morphological and phytochemical characteristics. Int. J. Med. Aromat. Plants Res..

[42] Yasuj S.F.M., Najafian S., Hosseinifarahi M. (2023). Comparison monoterpene and sesquiterpene, herbage yield of *Satureja hortensis*, *Thymus vulgaris*, and *Salvia officinalis* from the Fars province of Iran. Nat. Prod. Res..

[43] Štrbac F., Bosco A., Maurelli M.P., Ratajac R., Stojanović D., Simin N., Orčić D., Pušić I., Krnjajić S., Sotiraki S. (2022). Anthelmintic properties of essential oils to control gastrointestinal nematodes in sheep—In vitro and in vivo studies. Vet. Sci..

[44] Elmdoustazar P., Aydın B., Önal M., Yuca H., Karadayı M., Gülşahin Y., Demirci B., Karakaya S., Güvenalp Z. (2025). Phytochemical Traits and Biological Activity of *Satureja hortensis* and *Satureja macrantha* as Culinary Spices Using GC–MS/MS and LC–MS/MS Techniques. Food Sci. Nutr..

[45] Sharifi-Rad J., Sharifi-Rad M., Hoseini-Alfatemi S.M., Iriti M., Sharifi-Rad M., Sharifi-Rad M. (2015). Composition, cytotoxic and antimicrobial activities of *Satureja intermedia* C.A. mey essential oil. Int. J. Mol. Sci..

[46] Sadeghi I., Yousefzadi M., Behmanesh M., Sharifi M., Moradi A. (2013). In vitro cytotoxic and antimicrobial activity of essential oil from *Satureja intermedia*. Iran. Red. Crescent. Med. J..

[47] Aghaaliakbari B., Mojtahedi M.M., Hajiaghaee R., Abaee M.S., Besati M., Ghafarzadegan R., Tavakoli S. (2024). Chemical composition, Cholinesterase inhibitory effect and Cytotoxic activity study of essential oils extracted from different parts of *Satureja isophylla* Rech. f. Jundishapur J. Nat. Pharm. Prod..

[48] Bordbar F., Payandeh M., Mirtadzadini M. (2020). *Satureja kermanica* (Lamiaceae) a new species from south-east of Iran, inferred from molecular and morphological evidence. Phytotaxa.

[49] Hasheminya S.M., Mokarram R.R., Ghanbarzadeh B., Hamishekar H., Kafil H.S., Dehghannya J. (2019). Development and characterization of biocomposite films made from kefiran, carboxymethyl cellulose and *Satureja khuzestanica* essential oil. Food. Chem..

[50] Shahmohammadi M., Bahmani M., Ghaneialvar H., Abbasi N. (2021). Extraction and identification of the components of *Thymbra spicata* L. and *Satureja khuzestanica* Jamzad Oils native to Ilam province by headspace-solid phase microextraction (HS-SPME) and gas chromatography-mass spectrometry (GC-MS). J. Med. Pharm. Chem. Res..

[51] Mahboubi M., Kazempour N. (2016). The antibacterial activity of *Satureja khuzestanica* essential oil against clinical isolates of *E. coli*. J. Nat. Pharm. Prod..

[52] Dodoš T., Rajčević N., Janaćković P., Vujisić L., Marin P.D. (2019). Essential oil profile in relation to geographic origin and plant organ of *Satureja kitaibelii* Wierzb. ex Heuff. Ind. Crop. Prod..

[53] Gavrilov G.V., Nikolova M.T., Gavrilova A.B., Pashev A.S. (2025). Essential oil composition of *Satureja kitaibelii* Wierzb. ex Heuff. from the central Danubian plain, Bulgaria. Pharmacia.

[54] Dimitrijević M.V., Miladinović L.C., Marković M.S., Arsić B., Mihajilov-Krstev T.M., Miladinović D.L. (2024). New facts on the antimicrobial Essential oil of *Satureja kitaibelii*. Chem. Biodivers..

[55] Hudz N., Makowicz E., Shanaida M., Białoń M., Jasicka-Misiak I., Yezerska O., Svydenko L., Wieczorek P.P. (2020). Phytochemical evaluation of tinctures and essential oil obtained from *Satureja montana* herb. Molecules.

[56] Rezende D., Oliveira C.D., Batista L.R., Ferreira V.R., Brandão R.M., Caetano A.R., Alves M.V., Cardoso M.G. (2022). Bactericidal and antioxidant effects of essential oils from *Satureja montana* L., *Myristica fragrans* H. and *Cymbopogon flexuosus*. Lett. Appl. Microbiol..

[57] Caprioli G., Lupidi G., Maggi F. (2019). Comparison of chemical composition and antioxidant activities of two winter savory subspecies (*Satureja montana* subsp. *variegata* and *Satureja montana* subsp. *montana*) cultivated in Northern Italy. Nat. Prod. Res..

[58] Zawiślak G., Nurzyńska-Wierdak R. (2017). Plant morphological parameters and yield of winter savory depending on the method of plantation establishment. Acta. Sci. Pol. Hortorum Cultus.

[59] Vitanza L., Maccelli A., Marazzato M., Scazzocchio F., Comanducci A., Fornarini S., Crestoni M.E., Filippi A., Fraschetti C., Rinaldi F. (2019). *Satureja montana* L. essential oil and its antimicrobial activity alone or in combination with gentamicin. Microb. Pathog..

[60] Nikolić M., Jovanović K.K., Marković T., Marković D., Gligorijević N., Radulović S., Soković M. (2014). Chemical composition, antimicrobial, and cytotoxic properties of five Lamiaceae essential oils. Ind. Crops Prod..

[61] Dimitrijević M., Stojanović-Radić Z., Radulović N., Nešić M. (2025). Chemical Composition and Antifungal Effect of the Essential Oils of *Thymus vulgaris* L., *Origanum vulgare* L., and *Satureja montana* L. Against Clinical Isolates of *Candida* spp.. Chem. Biodivers..

[62] Ratajac R., Štrbac F., Petrović J., Stojanov I., Pušić I., Kačarević T., Simin N., Orčić D., Stojanović D., Hailan W.A. (2025). Evaluation of antibacterial potential of *Satureja montana* L., *Ocimum basilicum* L. and *Salvia officinalis* L. essential oils against reproductive tract pathogens in cattle and their toxicity impact on endometrial and kidney cells. Pak. Vet. J..

[63] Basha E., Mamoçi E., Sharma A., Hodaj-Çeliku E., Zejnelhoxha S., Medeleanu M.L., Socaci S.A., Bisha B. (2025). Essential Oils from Wild Albanian Lamiaceae: GC-MS Profiling, Biological Activity, and Enhanced Delivery via Nanoencapsulation. Molecules.

[64] Karimi E., Ghasemnezhad A., Hadian J., Ghorbanpour M. (2016). Assessment of EO constituents and main agro-morphological variability in *Satureja mutica* populations. Braz. J. Bot..

[65] Barzegar S., Zare M.R., Shojaei F., Zareshahrabadi Z., Koohi-Hosseinabadi O., Saharkhiz M.J., Iraji A., Zomorodian K., Khorram M. (2021). Core-shell chitosan/PVA-based nanofibrous scaffolds loaded with *Satureja mutica* or *Oliveria decumbens* essential oils as enhanced antimicrobial wound dressing. Int. J. Pharm..

[66] Al-Maharik N., Jaradat N. (2021). Phytochemical Profile, Antimicrobial, Cytotoxic, and Antioxidant Activities of Fresh and Air-Dried *Satureja nabateorum* Essential Oils. Molecules.

[67] Fitsiou E., Anestopoulos I., Chlichlia K., Galanis A., Kourkoutas I., Panayiotidis M.I., Pappa A. (2016). Antioxidant and antiproliferative properties of the essential oils of *Satureja thymbra* and *Satureja parnassica* and their major constituents. Anticancer Res..

[68] Arman M., Pirian K., Alinaghizadeh M., Khosheghbal F., Nahavandi R., Jahromi S.T. (2022). Study of compounds, cytotoxicity and biological activities of EO of *Satureja rechingeri* Jamzad. Adv. Tradit. Med..

[69] Maktabi S., Rashnavadi R., Tabandeh M.R., Sourestani M.M. (2024). Effective Inhibition of *Listeria monocytogenes* Biofilm Formation by *Satureja rechingeri* Essential Oil: Mechanisms and Implications. Curr. Microbiol..

[70] Hasheminya S.M., Dehghannya J. (2025). Development and characterization of *Plantago major* L. seeds mucilage-polyvinyl alcohol nano-biocomposite films incorporating *Satureja sahendica* Bornm. Essential oil nanoemulsion and zinc oxide nanoparticles. Int. J. Biol. Macromol..

[71] Bektašević M., Carev I., Roje M., Jurin M., Politeo O. (2017). Phytochemical composition and antioxidant activities of the essential oil and extracts of *Satureja subspicata* Vis. growing in Bosnia and Herzegovina. Chem. Biodivers..

[72] Khalil N., El-Jalel L., Yousif M., Gonaid M. (2020). Altitude impact on the chemical profile and biological activities of *Satureja thymbra* L. essential oil. BMC Complement. Med. Ther..

[73] Anastasiou T.I., Mandalakis M., Krigas N., Vézignol T., Lazari D., Katharios P., Dailianis T., Antonopoulou E. (2020). Comparative Evaluation of essential oils from Medicinal-Aromatic Plants of Greece: Chemical Composition, Antioxidant Capacity and Antimicrobial Activity against Bacterial Fish Pathogens. Molecules.

[74] Jaradat N., Hawash M., Al-Maharik N., Qadi M. (2025). Phytochemical Profiling and Bioactive Properties of Essential Oils from Endemic Palestinian *Satureja thymbrifolia*. Chem. Biodivers..

[75] Sierra-Quitian A.G., Prieto-Rodríguez J.A., Patiño-Ladino O.J. (2025). Insecticidal Activity of Monoterpenoids Against *Sitophilus zeamais* Motschulsky and *Tribolium castaneum* Herbst: Preliminary Structure–Activity Relationship Study. Int. J. Mol. Sci..

[76] Gopčević K., Grujić S., Arsenijević J., Karadžić I., Izrael-Živković L., Maksimović Z. (2019). Phytochemical properties of *Satureja kitaibelii*, potential natural antioxidants: A new insight. Plant. Food Hum. Nutr..

[77] Gopčević K., Grujić S., Arsenijević J., Džamić A., Veličković I., Izrael-Živković L., Medić A., Mudrić J., Soković M., Đurić A. (2022). Bioactivity and phenolics profile of aqueous and ethyl acetate extracts of *Satureja kitaibelii* Wierzb. ex Heuff. obtained by ultrasound-assisted extraction. Sci. Rep..

[78] Boroja T., Katanić J., Rosić G., Selaković D., Joksimović J., Mišić D., Stanković V., Jovičić N., Mihailović V. (2018). Summer savory (*Satureja hortensis* L.) extract: Phytochemical profile and modulation of cisplatin-induced liver, renal and testicular toxicity. Food Chem. Toxicol..

[79] Kınoğlu B.K., Gülçin İ., Gören A.C. (2024). Quantification of secondary metabolites of *Satureja pilosa* (Lamiaceae) by LC-HRMS and evaluation of antioxidant and cholinergic activities. Rec. Nat. Prod..

[80] Malmir M., Gohari A.R., Saeidnia S., Silva O. (2015). A new bioactive monoterpene–flavonoid from *Satureja khuzistanica*. Fitoterapia.

[81] Moghadam S.E., Ebrahimi S.N., Gafner F., Ochola J.B., Marubu R.M., Lwande W., Haller B.F., Salehi P., Hamburger M. (2015). Metabolite profiling for caffeic acid oligomers in *Satureja biflora*. Ind. Crop. Prod..

[82] Čakar J., Lojo N.K., Haverić A., Hadžić M., Lasić L., Zeljković S.Ć., Haverić S., Bajrović K. (2018). *Satureja subspicata* and *S. horvatii* Extracts induce overexpression of the BCl-2 family of anti-apoptotic genes and reduce micronuclei frequency in mice. Nat. Prod. Commun..

[83] Emre İ., Kurşat M., Yilmaz Ö., Erecevit P. (2021). Chemical compositions, radical scavenging capacities and antimicrobial activities in seeds of *Satureja hortensis* L. and *Mentha spicata* L. subsp. *spicata* from Turkey. Braz. J. Biol..

[84] Aras A., Bursal E., Alan Y., Turkan F., Alkan H., Kılıç Ö. (2018). Polyphenolic Content, Antioxidant Potential and Antimicrobial Activity of *Satureja boissieri*. Iran. J. Chem. Chem. Eng..

[85] Yıldız A.N., Çarıkçı S., Dirmenci T., Kartal M., Gülcin I., Gören A.C. (2025). Secondary Metabolite Profiling of *Satureja aintabensis* P.H. Davis and *Satureja spicigera* (K. Koch) Boiss. by LC-HRMS and Evaluation of Antioxidant and Anticholinergic Activities. Life.

[86] Teofilović B., Gligorić E., Ninić M., Vukmirović S., Gagić Ž., Mandić-Kovačević N., Tubić B., Đukanović Đ., Grujić-Letić N. (2025). Green Extraction Combined with Chemometric Approach: Profiling Phytochemicals and Antioxidant Properties of Ten Species of the Lamiaceae Family. Separations.

[87] Samani R.M., D’Urso G., Nazzaro F., Fratianni F., Masullo M., Piacente S. (2024). Phytochemical Investigation and Biofilm-Inhibitory Activity of Bachtiari Savory (*Satureja bachtiarica* Bunge) Aerial Parts. Plants.

[88] Mašković P., Veličković V., Mitić M., Đurović S., Zeković Z., Radojković M., Cvetanović A., Švarc-Gajić J., Vujić J. (2017). Summer savory extracts prepared by novel extraction methods resulted in enhanced biological activity. Ind. Crop Prod..

[89] Rahimmalek M., Afshari M., Sarfaraz D., Miroliaei M. (2020). Using HPLC and multivariate analyses to investigate variations in the polyphenolic compounds as well as antioxidant and antiglycative activities of some Lamiaceae species native to Iran. Ind. Crops Prod..

[90] Mašković J.M., Jakovljević V., Živković V., Mitić M., Kurćubić L.V., Mitić J., Mašković P.Z. (2024). Optimization of Ultrasound-Assisted Extraction of Phenolics from *Satureja hortensis* L. and Antioxidant Activity: Response Surface Methodology Approach. Processes.

[91] Buyukyildirim T., Ocal Ozdamar F., Baysal Furtana G., Gok H.N., Ozek T., Orhan I.E., Senol Deniz F.S., Duman H. (2025). Enzyme inhibitory and antioxidant potential with phytochemical analysis of *Satureja hasturkii* H. Duman & Dirmenci: A new record from Türkiye. Chem. Biodivers..

[92] Abdelshafeek K.A., Osman A.F., Mouneir S.M., Elhenawy A.A., Abdallah W.E. (2023). Phytochemical profile, comparative evaluation of *Satureja montana* alcoholic extract for antioxidants, anti-inflammatory and molecular docking studies. BMC Complement. Med. Ther..

[93] Jakupović L., Strawa J.W., Nižić Nodilo L., Marijan M., Hafner A., Jakimiuk K., Tomczykowa M., Tomczyk M., Končić M.Z. (2025). Cosmeceutical and Wound-Healing Activities of Green Hydroxypropyl-β-Cyclodextrin-Glycerol-Based *Satureja montana* Extracts. Molecules.

[94] Pavlović O.M., Kolarević S., Đorđević J., Jovanović Marić J., Lunić T., Mandić M., Kračun Kolarević M., Živković J., Alimpić Aradski A., Marin P.D. (2021). A study of phytochemistry, genoprotective activity, and antitumor effects of extracts of the selected Lamiaceae species. Plants.

[95] Raadani A., Boulila A., Yangui I., Boussaid M., Messaoud C., Ben Elhadj Ali I. (2024). Variation in Phenolic Content, Antioxidant Activity and Alpha-amylase and Acetylcholinesterase Inhibitory Capacities of Different Extracts from Tunisian *Satureja barceloi* (Willk) L.. Chem. Biodivers..

[96] Vrancheva R., Dincheva I., Aneva I., Georgiev V., Pavlov A. (2022). GC-MS-based metabolite profiling of wild and in vitro growing plants of *Satureja montana* L.. C. R. Acad. Bulg. Sci..

[97] Paloukopoulou C., Ntagli O.S., Gherardi L., Dourdouni V., Filippou G., Alterio V., Giovannuzzi S., Massardi M.L., De Simone G., Ronca R. (2025). Depsides from *Origanum dictamnus* and *Satureja pilosa* as selective inhibitors of carbonic anhydrases: Isolation, structure elucidation, X-ray crystallography. Arch. Pharm..

[98] Šimunović K., Bucar F., Klančnik A., Pompei F., Paparella A., Smole Možina S. (2020). In vitro effect of the common culinary herb winter savory (*Satureja montana*) against the infamous food pathogen *Campylobacter jejuni*. Foods.

[99] Khani S., Seyedjavadi S.S., Zare-Zardini H., Hosseini H.M., Goudarzi M., Khatami S., Amani J., Imani Fooladi A.A., Razzaghi-Abyaneh M. (2019). Isolation and functional characterization of an antifungal hydrophilic peptide, Skh-AMP1, derived from *Satureja khuzistanica* leaves. Phytochemistry.

[100] Narchin F., Larijani K., Rustaiyan A., Ebrahimi S.N., Tafvizi F. (2018). Phytochemical synthesis of silver nanoparticles by two techniques using *Saturaja rechengri* Jamzad extract: Identifying and comparing in vitro anti-proliferative activities. Adv. Pharm. Bull..

[101] Cagal M.M., Taner G., Kalaycı S., Duman G. (2025). Enhanced antibacterial and genoprotective properties of nanoliposomal *Satureja hortensis* L. essential oil. Drug. Chem. Toxicol..

[102] Yuan Y., Hui X., Liu Z., Sun J., Raka R.N., Xiao J., Zhang Z., Wu H. (2025). Investigation of differential multi-mode antibacterial mechanisms of essential oils of *Satureja montana* L. and *Leptospermum scoparium* JR Forst. & G. Forst. against *Porphyromonas gingivalis*. BMC Complement. Med. Ther..

[103] Sharifi A., Mohammadzadeh A., Zahraei Salehi T., Mahmoodi P. (2018). Antibacterial, antibiofilm and antiquorum sensing effects of *Thymus daenensis* and *Satureja hortensis* essential oils against *Staphylococcus aureus* isolates. J. Appl. Microbiol..

[104] Maravić-Vlahoviček G., Kindl M., Andričević K., Obranić S., Vladimir-Knežević S. (2025). Modulatory Effects of *Satureja montana* L. Essential Oil on Biofilm Formation and Virulence Factors of *Pseudomonas aeruginosa*. Pharmaceuticals.

[105] Mandalakis M., Anastasiou T.I., Martou N., Keisaris S., Greveniotis V., Katharios P., Lazari D., Krigas N., Antonopoulou E. (2021). Antibacterial effects of essential oils of seven medicinal-aromatic plants against the fish pathogen *Aeromonas veronii* bv. sobria: To blend or not to blend?. Molecules.

[106] Harmati M., Gyukity-Sebestyen E., Dobra G., Terhes G., Urban E., Decsi G., Mimica-Dukić N., Lesjak M., Simin N., Pap B. (2017). Binary mixture of *Satureja hortensis* and *Origanum vulgare* subsp. *hirtum* essential oils: In vivo therapeutic efficiency against *Helicobacter pylori* infection. Helicobacter.

[107] Fratini F., Pecorini C., Resci I., Copelotti E., Nocera F.P., Najar B., Mancini S. (2025). Evaluation of the Synergistic Antimicrobial Activity of Essential Oils and Cecropin A Natural Peptide on Gram-Negative Bacteria. Animals.

[108] Alvand Z.M., Rahimi M., Rafati H. (2022). Interaction of a natural compound nanoemulsion with Gram negative and Gram positive bacterial membrane; a mechanism based study using a microfluidic chip and DESI technique. Int. J. Pharm..

[109] Rezaei A., Monfared-Hajishirkiaee R., Hosseinzadeh-Moghaddam S., Behzadi M., Shahangian S.S. (2025). Enhancing leachate management with antibacterial nanocomposites incorporating plant-based carbon dots and *Satureja khuzestanica* essential oils. Colloids. Surf. B.

[110] Abbasi A.T., Ebrahimi L., Farzaneh M. (2025). Antifungal efficacy of plant essential oil nanoemulsions against cucumber powdery mildew. Sci. Rep..

[111] Vanti G., Tomou E.M., Stojković D., Ćirić A., Bilia A.R., Skaltsa H. (2021). Nanovesicles loaded with *Origanum onites* and *Satureja thymbra* essential oils and their activity against food-borne pathogens and spoilage microorganisms. Molecules.

[112] Maral H., Oğuz M., Türkmen M., Soylu S. (2025). Chemical profile and bioactivity of essential oils from five Turkish thyme species against white mold fungal disease agent *Sclerotinia sclerotiorum*. Sci. Rep..

[113] Bǎieş M.H., Györke A., Cotuţiu V.D., Boros Z., Cozma-Petruț A., Filip L., Vlase L., Vlase A.M., Crişan G., Spînu M. (2023). The in vitro anticoccidial activity of some herbal extracts against *Eimeria* spp. oocysts isolated from piglets. Pathogens.

[114] Jahanshahi S., Kheirandish F., Kazemi B., Montazeri M., Fallahi S., Rouzbahani A.K., Mamaghani A.J. (2024). Investigating the Effect of *Satureja khuzestanica* Essential oil on MDR1 Gene Expression in *Leishmania major*. Acta. Parasitol..

[115] Les F., Galiffa V., Cásedas G., Moliner C., Maggi F., López V., Gómez-Rincón C. (2024). Essential Oils of Two Subspecies of *Satureja montana* L. against Gastrointestinal Parasite *Anisakis simplex* and Acetylcholinesterase Inhibition. Molecules.

[116] Štrbac F., Krnjajić S., Ratajac R., Rinaldi L., Musella V., Castagna F., Stojanović D., Simin N., Orčić D., Bosco A. (2025). Anthelmintic activity of winter savory (*Satureja montana* L.) essential oil against gastrointestinal nematodes of sheep. BMC Vet. Res..

[117] Mahmoudvand H., Badparva E., Baharvand Z., Lalehmarzi H.S. (2018). Anti-*Trichomonas vaginalis* activities and apoptotic effects of some Iranian medicinal plants. Trop. Biomed..

[118] Aboubaker D.H., Shaffie N.A., Shabana M.F., Abd Elghafour A., Ibrahim B.M. (2025). Protective role of savory essential oil on vital organs in rats against deleterious effects induced by lead acetate. Biotechnol. Rep..

[119] Asadipour M., Amirghofran Z. (2019). *Satureja hortensis* induces cell death and inhibited cell cycle progression in K562 myelogenous and Jurkat T cell leukemia cell lines. J. Immunoass. Immunochem..

[120] Vladić J., Ćebović T., Vidović S., Jokić S. (2020). Evaluation of anticancer activity of *Satureja montana* supercritical and spray-dried extracts on Ehrlich’s Ascites carcinoma bearing mice. Plants.

[121] Obeidnejad E., Kavoosi G., Saharkhiz M.J. (2024). Antioxidant, anti-amylase, anti-lipase, and efficiency of *Satureja* fatty acid on the anti-inflammatory parameters in lipopolysaccharide-stimulated macrophage through Nrf2/NF-kB/NADH oxidase pathway. Sci. Rep..

[122] Kheiri E., Bonab Z.H., Soltanzadeh H. (2025). Investigating the Expression of Apoptosis and Metastasis Genes in *Satureja khuzistanica* along with Nisin and Doxorubicin in Colorectal Cancer. Pharmacog. Res..

[123] Farzaneh Z., Kalantar K., Iraji A., Amirghofran Z. (2018). Inhibition of LPS-induced inflammatory responses by *Satureja hortensis* extracts in J774.1 macrophages. J. Immunoass. Immunochem..

[124] Abbasloo E., Dehghan F., Khaksari M., Najafipour H., Vahidi R., Dabiri S., Sepehri G., Asadikaram G. (2016). The anti-inflammatory properties of *Satureja khuzistanica* Jamzad EO attenuate the effects of traumatic brain injuries in rats. Sci. Rep..

[125] Vilmosh N., Georgieva-Kotetarova M., Kandilarov I., Zlatanova-Tenisheva H., Murdjeva M., Kirina V., Dimitrova S., Katsarova M., Denev P., Kostadinova I. (2024). Anti-inflammatory and in vitro antioxidant activities of *Satureja montana* dry extract. Fol. Med..

[126] Nasimi P., Vahdati A., Tabandeh M.R., Khatamsaz S. (2016). Cytoprotective and anti-apoptotic effects of *Satureja khuzestanica* essential oil against busulfan-mediated sperm damage and seminiferous tubules destruction in adult male mice. Andrologia.

[127] Abd El Tawab A.M., Shahin N.N., AbdelMohsen M.M. (2014). Protective effect of *Satureja montana* extract on cyclophosphamide-induced testicular injury in rats. Chem. Biol. Interact..

[128] Bostanabad M.A., Hiradfar A., Mohammadpoorasl A., Javadzadeh Y., Khalvati B., Alvandnezhad T. (2018). The effect of mucoadhesive gel containing *Satureja hortensis* extract 1% on severity of chemotherapy-induced mucositis pain in children: A randomized clinical trial. Int. J. Pediatr..

[129] Coban F., Lan Y., Yetisgin G., Yuca H., Aydın B., Angın H., Demirci B., Karakaya S. (2025). Phytochemical composition and bioactivities of *Satureja montana* L. and *Satureja hortensis* L.: Culinary herbs with antidiabetic, anticholinesterase, and antioxidant potential. PLoS ONE.

[130] Roosta S., Ghasemi F., Mokhayeri Y., Choobkar S., Nikbakht M.R., Falahi E. (2024). Effects of *Satureja Khuzestanica* supplementation on glycemic indices and lipid profile in type-2 diabetes patients: A randomized controlled clinical-trial. BMC Complement. Med. Ther..

[131] Mirderikvandi M., Khosravinia H., Parizadian Kavan B. (2020). Independent and combined effects of *Satureja khuzistanica* essential oils and acetic acid on prevalence and intensity of fatty liver syndrome in broiler chickens. J. Anim. Physiol. Anim. Nutr..

[132] Soodi M., Saeidnia S., Sharifzadeh M., Hajimehdipoor H., Dashti A., Sepand M.R., Moradi S. (2016). *Satureja bachtiarica* ameliorate beta-amyloid induced memory impairment, oxidative stress and cholinergic deficit in animal model of Alzheimer’s disease. Metab. Brain. Dis..

[133] Alishahi M., Halimi M., Khansari A., Yavari V. (2015). Extracts of *Oliviera decumbens* and *Satureja khuzestanica* as immunostimulants affect some innate immunity indices of *Cyprinus carpio* against *Aeromonas hydrophila* infection. Aquac. Res..

[134] Sánchez-Quintero M.J., Delgado J., Medina-Vera D., Becerra-Muñoz V.M., Queipo-Ortuño M.I., Estévez M., Plaza-Andrades I., Rodríguez-Capitán J., Sánchez P.L., Crespo-Leiro M.G. (2022). Beneficial effects of essential oils from the Mediterranean diet on gut microbiota and their metabolites in ischemic heart disease and type-2 diabetes mellitus. Nutrients.

[135] Demyashkin G., Tokov A., Belokopytov D., Shchekin V., Borovaya T., Lukash D., Yuferov D., Kulchenko N., Tarasov V., Blinova E. (2025). Effects of *Satureja montana* L. and *Origanum vulgare* L. Hydrolates in Rabbit Burn Wound Model: Evaluation of Inflammatory, Antioxidant Activity, and Pro-Regenerative Properties in the Skin. Int. J. Mol. Sci..

[136] Ilhan E., Cesur S., Guler E., Topal F., Albayrak D., Guncu M.M., Cam M.E., Taskin T., Sasmazel H.T., Aksu B. (2020). Development of *Satureja cuneifolia*-loaded sodium alginate/polyethylene glycol scaffolds produced by 3D-printing technology as a diabetic wound dressing material. Int. J. Biol. Macromol..

[137] Esmaeili-Mahani S., Ebrahimi B., Abbasnejad M., Rasoulian B., Sheibani V. (2015). *Satureja khuzestanica* prevents the development of morphine analgesic tolerance through suppression of spinal glial cell activation in rats. J. Nat. Med..

[138] Rabiei Z., Shirchi M., Rafieian-Kopaei M., Asgharzade S. (2022). Effects of *Satureja bachtiarica* essential oil in preventing seizure in pentylenetetrazol-kindled mice. Basic Clin. Neurosci..

[139] Muthukumar A., Mittal S., Choezom T., Bhavani K., Das K., Joyce N., Almuqbil M., Almadani M.E., Ahmad F., Yasmin F. (2024). Evaluation of the cardioprotective activity of summer savory (*Satureja hortensis* L.) extract in experimental rats with Isoproterenol-induced myocardial infarction. J. King Saud. Univ. Sci..

[140] Nasiri M., Barzegar M., Sahari M.A., Niakousari M. (2018). Application of tragacanth gum impregnated with *Satureja khuzistanica* essential oil as a natural coating for enhancement of postharvest quality and shelf life of button mushroom (*Agaricus bisporus*). Int. J. Biol. Macromol..

[141] Azimi M., Neyriz Naghadehi M., Moulodi F., Razavi Rohani S.M., Alizade Khaledabad M. (2018). The Effects of *Satureja hortensis* L. essential oil on the Growth and Survival of *Salmonella typhimorium* in Minced Poultry Meat During Refrigerated Storage. J. Kermanshah. Univ. Med. Sci..

[142] Toplu Y., Önlü H. (2025). Anti-Listerial Effects of *Satureja hortensis* Essential Oils in Ready-to-Eat Poultry Meat Stored at Different Temperatures. Microbiol. Res..

[143] Oliveira-Pinto P.R., Oliveira-Fernandes J., Mariz-Ponte N., Monge-Mora P., Guido L.F., Fernandes-Ferreira M., Sousa R.M.O.F., Santos C. (2025). *Satureja montana* L. essential oil and montmorillonite nanoclay modulate the phenylpropanoid pathway and polyphenols biosynthesis of tomato plants suffering from bacterial spot disease. Planta.

[144] Abbad I., Soulaimani B., Iriti M., Barakate M. (2025). Chemical Composition and Synergistic Antimicrobial Effects of Essential Oils From Four Commonly Used *Satureja* Species in Combination With Two Conventional Antibiotics. Chem. Biodivers..

[145] Ahmadvand H. (2014). Amelioration of altered antioxidant enzyme activity by *Satureja khuzistanica* essential oil in alloxan-induced diabetic rats. Chin. J. Nat. Med..

[146] Alvand Z.M., Rahimi M., Rafati H. (2021). A microfluidic chip for visual investigation of the interaction of nanoemulsion of *Satureja khuzistanica* essential oil and a model gram-negative bacteria. Int. J. Pharm..

[147] Asadi-Samani M., Rafieian-Kopaei M., Lorigooini Z., Shirzad H. (2019). A screening of anti breast cancer effects and antioxidant activity of twenty medicinal plants gathered from Chaharmahal va Bakhtyari province. Iran. J. Pharm. Pharmacogn. Res..

[148] Asghari M.H., Babaei E., Fallah M., Mahmoodifar F. (2018). A comparative study on the analgesic properties of five members of Lamiaceae family using two pain models. Res. J. Pharm..

[149] Băieş M.-H., Gherman C., Boros Z., Olah D., Vlase A.-M., Cozma-Petruț A., Györke A., Miere D., Vlase L., Crișan G. (2022). The Effects of *Allium sativum* L., *Artemisia absinthium* L., *Cucurbita pepo* L., *Coriandrum sativum* L., *Satureja hortensis* L. and *Calendula officinalis* L. on the Embryogenesis of *Ascaris suum* Eggs during an In Vitro Experimental Study. Pathogens.

[150] Bartels N., Argyropoulou A., Al-Ahmad A., Hellwig E., Skaltsounis A.L., Wittmer A., Vach K., Karygianni L. (2025). Antibiofilm potential of plant extracts: Inhibiting oral microorganisms and *Streptococcus mutans*. Front. Dent. Med..

[151] Baseri M., Naseri A., Radmand F., Hamishehkar H., Memar M.Y., Ebrahimi A., Asnaashari S., Kouhsoltani M. (2025). Effect of nano liposomal herbal extracts against biofilm formation and adherence of *Streptococcus mutans*. Sci. Rep..

[152] Cagnoli G., Bertelloni F., Ebani V.V. (2024). In Vitro Antibacterial Activity of Essential Oils from *Origanum vulgare*, *Satureja montana*, *Thymus vulgaris*, and Their Blend Against Necrotoxigenic (NTEC), Enteropathogenic (EPEC), and Shiga-Toxin Producing *Escherichia coli* (STEC) Isolates. Pathogens.

[153] Esmaeilbeig M., Kouhpayeh S.A., Amirghofran Z. (2015). An Investigation of the growth inhibitory capacity of several medicinal plants from Iran on tumor cell lines. Iran. J. Cancer Prev..

[154] Hassanabadi N., Meymand Z.M., Ashrafzadeh A., Sharififar F. (2024). Antioxidant and cytotoxicity activity of a nanoemulsion from *Satureja kermanica* (Lamiaceae). Ann. Pharm. Fr..

[155] Hickl J., Argyropoulou A., Al-Ahmad A., Hellwig E., Skaltsounis A.L., Wittmer A., Vach K., Karygianni L. (2024). Unleashing nature’s defense: Potent antimicrobial power of plant extracts against oral pathogens and *Streptococcus mutans* biofilms. Front. Oral. Health.

[156] Jovanova B., Panovska T.K. (2019). Evaluation of the antioxidant effects and cytotoxic potential of selected herbs used in traditional medicine. J. Anim. Plant Sci..

[157] Masoum S., Samadi N., Mehrara B., Mahboubi M. (2018). Otentiality of independent component regression in assessment of the peaks responsible for antimicrobial activity of *Satureja hortensis* L. and *Oliveria decumbens* Vent. using GC–MS. J. Iran. Chem. Soc..

[158] Mohammadi-Ziveh Z., Mirhosseini S.A., Hosseini H.M. (2020). *Satureja khuzestanica* mediated synthesis of silver nanoparticles and its evaluation of antineoplastic activity to combat colorectal cancer cell line. Iran. J. Pharm. Res..

[159] Moreira S.A., Pintado M.E., Saraiva J.A. (2020). Optimization of antioxidant activity and bioactive compounds extraction of winter savory leaves by high hydrostatic pressure. High Press. Res..

[160] Mosaddegh M., Irani M. (2018). Inhibition test of heme detoxification (ITHD) as an approach for detecting antimalarial agents in medicinal plants. Res. J. Pharmacogn..

[161] Payandeh M., Ahmadyousefi M., Alizadeh H., Zahedifar M. (2022). Chitosan nanocomposite incorporated *Satureja kermanica* essential oil and extract: Synthesis, characterization and antifungal assay. Int. J. Biol. Macromol..

[162] Pereira G., Faria J.M.S. (2024). Activity of *Satureja montana* Allelochemical Volatiles Against the Pinewood Nematode. Chem. Proc..

[163] Raikwar G., Mohan S., Dahiya P. (2025). Combined antibacterial effect of essential oils from three Indian medicinal plants and antibiotic tetracycline on MRSA using simplex centroid mixture design. Sci. Rep..

[164] Ramezani M., Ehtesham-Gharaee M., Khazaie M., Behravan J. (2016). *Satureja hortensis* L. methanolic extract and essential oil exhibit antitumor activity. J. Essent. Oil Bear. Plants.

[165] Ratajac R., Pavlićević A., Petrović J., Stojanov I., Orčić D., Štrbac F., Simin N. (2024). In vitro evaluation of acaricidal efficacy of selected essential oils against *Dermanyssus gallinae*. Pak. Vet. J..

[166] Saboori K., Nassira M., Safari M., Namdar N., Montaseri Z., Osanloo M. (2025). Antibacterial activity of a conventional hydrogel and a nanoparticle based hydrogel containing *Satureja khuzestanica* essential oil. Sci. Rep..

[167] Shanaida M. (2018). Antioxidant activity of essential oils obtained from aerial part of some Lamiaceae species. Int. J. Green Pharm..

[168] Valentini F., Colasanti I.A., Zaratti C., Filimon D., Macchia A., Neri A., Relucenti M., Reverberi M., Allegrini I., Guerriero E. (2025). TiO_2_ and CaCO_3_ Microparticles Produced in Aqueous Extracts from *Satureja montana:* Synthesis, Characterization, and Preliminary Antimicrobial Test. Molecules.

[169] Yuan X., Cao D., Xiang Y., Jiang X., Liu J., Bi K., Dong X., Wu T., Zhang Y. (2024). Antifungal activity of essential oils and their potential synergistic effect with amphotericin B. Sci. Rep..

[170] Zeidán-Chuliá F., Keskin M., Könönen E., Uitto V.J., Söderling E., Moreira J.C., Gürsoy U.K. (2015). Antibacterial and antigelatinolytic effects of *Satureja hortensis* L. essential oil on epithelial cells exposed to *Fusobacterium nucleatum*. J. Med. Food..

[171] Zomorodian K., Ghadiri P., Saharkhiz M.J., Moein M.R., Mehriar P., Bahrani F., Golzar T., Pakshir K., Fani M.M. (2015). Antimicrobial activity of seven essential oils from Iranian aromatic plants against common causes of oral infections. Jundishapur J. Microbiol..

