# Supplementary material for: Current Knowledge of the Genus Satureja: A Comprehensive Review of Its Traditional Use, Phytochemistry, Pharmacological Activity and Non-Medical Applications
Source: Pharmaceuticals (Basel). 2026 May 31;19(6):875. doi: 10.3390/ph19060875 (PMC13304844; doi:10.3390/ph19060875)
Supplement: Supplementary file 1 [file pharmaceuticals-19-00875-s001.zip › pharmaceuticals-4305861-supplementary.pdf]

SUPPORTING INFORMATION

Current knowledge of the genus *Satureja*: a comprehensive review of its traditional use, phytochemistry, pharmacological activity and non-medical applications

Marah Alburquan,[1] Katalin Veres,[1] Judit Hohmann[1,2]*

[1]Institute of Pharmacognosy, University of Szeged, Eötvös Str. 6, 6720 Szeged, Hungary

[2]HUN-REN–USZ Biologically Active Natural Products Research Group, University of Szeged, Eötvös u. 6, H-6720 Szeged, Hungary

Table S1. Biological activities of *Satureja* species as reported in the original research articles reviewed.

Activity	Species	Tested component	Test performed	Effects	Reference
Antibacterial	*S. montana*	MeOH and EtOH extracts	Broth microdilution assay against *S. aureus* and *E. coli*	MIC values ranged from 22.10 ± 16.97 to 324.10 ± 208.54 (µg/mL).	Kremer et al., 2015
		EO	Broth microdilution assay against eight bacteria	The EO showed the strongest antibacterial activity (MIC 30.0–630.0 µg/mL, MBC 60.0–250.0 µg/mL)	Nikolic et al., 2014
		EO	Broth microdilution assay and biofilm inhibition assay against both clinical and reference strains of *S. aureus, L. monocytogenes* and *E. coli*	Against *S. aureus*, MIC values was in the range of 0.39–0.78 mg/mL and MBC of 0.78 mg/mL. Against *L. monocytogenes,* MIC was in the range 0.78–1.56 mg/mL and MBC in the range 1.56-3.12 mg/mL. Against *E. coli*, MIC and MBC values for EO coincided in the range 1.56–3.12 mg/mL.	Vitanza et al., 2019
		EO	Broth microdilution assay against pathogenic *E.coli* and *S. aureus*	MIC and MBC was 6.25 µl/mL against *E. coli*, and 50 µl/mL against *S. aureus.*	Rezende et al., 2022
		EO, EtOH extract, and compounds	Broth microdilution method against *Campylobacter jejuni;* checkerboard assay was used to determine the potential synergistic activity; ethidium bromide accumulation assay was applied to investigate the effux pump inhibitory effect against C. jejuni; membrane disruptive effects were studied by membrane integrity assay	The ethanolic extract (MIC 250 mg/L) was 4-fold effective than the EO against *C. jejuni*. Carvacrol (**5**), thymol (**7**), and thymoquinone showed MIC value 31.25 mg/L. The herb extract disrupted the membrane integrity (>80%) and inhibited efflux pumps by 2-folds. A strong synergistic activity between carvacrol (**5**) and thymol (**7**) was observed (FICi 0.2) was proved.	Šimunovic´ et al., 2020
		EO	Disc diffussion, agar dilution and microdilution tests against field isolates and reference bacterial strains of the most important pathogens of the cow: endometrium *Truperella pyogenes, Escherichia coli, Pasteurella spp., Staphylococcus aureus* and Streptococcus spp.	In disk diffusion assays, no bacterial isolates showed resistance to *S. montana* EO, and large zones of inhibition were observed.<br>MIC values were consistently <1 mg/mL using both agar dilution and microdilution methods, indicating high potency.	Ratajac et al., 2025
		EO	The MIC values were determined by the broth microdilution method, and sub-inhibitory concentrations were further evaluated for effects on planktonic growth and biofilm formation.	The EO showed a minimum inhibitory concentration of 4 mg/mL and a biphasic effect, inhibiting planktonic growth at higher concentrations (36–58% reduction; $p < 0.05$). It promoted biofilm formation at 0.125–0.5 mg/mL ($p < 0.05$), inhibited swarming at 0.5 mg/mL, and increased pyocyanin production.	Maravic-Vlahovicek et al., 2025

		EO	MIC, MBC and FIC was determined for the essensial oil alone and when combined with *Cinnamomum zeylanicum, Citrus bergamia* and cecropin A against *Escherichia coli* and *Salmonella enterica* serovar Typhimurium.	The EO showed good antibacterial activity against both *E. coli* (MIC=1:2048) and *S.* Typhimurium (MIC=1:2048). Combinations with *Cinnamomum zeylanicum*, *Citrus bergamia*, or cecropin A enhanced antibacterial effects.	Fratini et al., 2025
		EO	EOs from *Origanum vulgare, Satureja montana, Thymus vulgaris,* and their blend were tested for antibacterial activity against necrotoxigenic (NTEC), enteropathogenic (EPEC), and shiga-toxin producing *Escherichia coli* (STEC) isolates by broth microdilution method.	MIC values between 0.039% and 0.156% were found for O. vulgare EO, between 0.0195% and 0.156% for *S. montana*, and between ≤0.0195% and 0.039% for the blend.	Cagnoli et al., 2024
		TiO_2 and $CaCO_3$ Microparticles of *S. montana* extract	Aqueous extracts of *S. montana* were used for the green synthesis of TiO_2 and $CaCO_3$ particles, which were characterized by XRD, Raman, IR spectroscopy, and SEM, and evaluated for antimicrobial activity under indoor passive sedimentation conditions.	While TiO_2 anatase particles (400–600 nm) promoted microbial growth as their concentration increased, *Satureja montana*-functionalized $CaCO_3$ microparticles (~1 µm) exhibited strong, dose-dependent antimicrobial activity, achieving near-complete inhibition at 50 mg/mL.	Valentini et al., 2025
		EO, either alone or formulated with montmorillonite nanoclay	The objective of this study was to evaluate the effect on the phenylpropanoid defense pathway in tomato plants infected with *Xanthomonas euvesicatoria.*	Foliar application of the EO altered phenolic production and phenylpropanoid gene expression, indicating antimicrobial and plant defense–inducing activity.	Oliveira-Pinto et al., 2025
		EO	Micro broth dilution assay against six common foodborne pathogens and one fungi.	MIC values were in the range of 0.312–2.5 mg/mL	Basha et al., 2025
		EO	Antibacterial activity was assessed against *Porphyromonas gingivalis* using the disk diffusion method. MIC, MBC, and bacterial growth curves were determined and the effects on hemagglutination, hemolytic, black pigmentation formation, autoaggregation, hydrophobicity, biofilm formation and virulence gene expression were evaluated.	The EO showed strong antibacterial activity with an inhibition zone of 42.06 ± 1.62 mm, MIC 71.33 µg/mL, and MBC 142.66 µg/mL, and sustained inhibition in growth curves. It inhibited *P. gingivalis* hemagglutination and hemolysis ($p < 0.05$), reduced heme accumulation at 1/8–1/2 MIC, decreased hydrophobicity to <50% at 1/4–1/2 MIC, and inhibited >85% of biofilm formation at MIC ($p < 0.05$).	Yuan et al., 2025

		EO	The antibacterial evaluation was performed using disc diffusion and micro well dillution assays against MRSA and *Staphylococcus aureus* clinical isolates (SACI 1-4), Determentation of the antibiofilm effect was performed using crystal violet assay against MRSA.	Compared to other tested medicinal plants, *S. montana* EO exhibited the most potent activity, with the largest zones of inhibition observed against SACI-2 (12.0 ± 1.0 mm), SACI-4 (12.66 ± 1.15 mm), and MRSA (ATCC 43300) (12.0 ± 2.6 mm) and exhibited MIC values ranging from 3.125 to 12.5 µL/mL and MBC values ranging from 6.25 to 12.5 µL /mL.	Raikwar et al., 2025
	S. subspicata	MeOH and EtOH extracts	Broth microdilution assay against *S. aureus* and *E. coli*	MIC values ranged from 77.16 ± 5.31 to 518.57 ± 128.29 µg/mL	Kremer et al., 2015
	S. calamintha spp. *nepeta*	EO	Disk diffusion method and microdilution assay against *S. aureus, B. subtilis* and *E. coli*	A more pronounced effect was recorded against *E. coli*, with 25.67 ± 0.58 mm diameters and MIC value of 1.49 ± 0.00 µg/mL. While the lowest activity was against *S. aureus,* with inhibition diameters of 14 ± 0.00 and MIC value of 5.96 ± 0.00 µg/mL.	El Brahimi et al., 2023
	S. avromanica	EO and MeOH extract	Disc diffusion method, MIC determination against six Gram-positive and three Gram-negative bacteria.	*B. cereus, B. pumilus,* and *S. aureus* were all susceptible to the EO and the extract.	Abdali et al., 2017
	S. hortensis	Extracts containing flavonoids, vitamins and fatty acids from the seeds of *S. hortensis*	Well agar method against *E. coli, K. pneumoniae, S. aureus* and *Bacillus megaterium*	Extract containing vitamins had the strongest antibacterial activities (12.1 ± 0.1 mm to 23.4 ± 0.3 mm). The effect of the extract rich in flavonoids was between 15.3 ± 0.3 mm – 35.1 ± 0.1 mm.	Emre et al., 2021
		EO	Micro dilution assay against *Helicobacter pylori*	MIC value of EO was 2 µL/mL.	Lesjak et al., 2016
		EO	Broth microdilution assay, microtitre plate test for antibiofilm inhibition, analysis of gene expression involved in quorum sensing (QS) inhibitory activity in *S. aureus*	MIC and MBC were both 0.125 µl/ml. The EO displayed significant inhibitory effects on biofilm formation and disrupted preformed *S. aureus* biofilms at sub-MIC concentration.	Sharifi et al., 2018
		EO	*Fusobacterium nucleatum* membrane permeability was tested by measuring the *N*-phenyl-1-naphthylamine uptake and bacterial viability was tested by Live/Dead BacLight kit.	At concentrations of 5, 10, and 25 µL/mL the EO increased the outer membrane permeability and decreased the ratio of live/dead bacteria in a range of time from 0 to 5 min.	Zeidan-Chulia et al., 2015
		EO of different origin	Disk diffusion method and broth micro dilution assay against *C. albicans*, *S. aureus* and *E. coli.*	MICs were 0.025 - 0.500 µL/mL, MBC were 0.050 - 1.000 µL/mL while inhibition zone diameter was against *C. albicans* 17.10–26.10 mm, *S. aureus* 6.80–17.00 mm, and *E. coli* 9.20–15.05 mm.	Masoum et al., 2018
		EO and MeOH extract	Broth microdilution method against *Agrobacterium tumefacien*	MeOH extract showed an MIC value of 3 mg/mL while the EO inhibited *A. tumefaciens* at all dilutions tested.	Ramezani et al., 2016

		Mixture of *S. hortensis* and *O. vulgare* subsp. *hirtum* EOs	in vivo anti-*H. pylori* effect in mice models	The EO mixture successfully eradicated *H. pylori* in 70% of the mice.	Harmati et al., 2017
		EtOH extract	Broth microdilution assay and disk diffusion method against *S. aureus, B. subtilis*, *E. coli* and *P. vulgaris*	Zones of inhibition ranged from 19 to 277 mm. MIC ranged from 4.8 to 9.7 µg/mL.	Huwaimel et al., 2023
		EO	Broth microdilution method against *E. coli* and *S. aureus*	MIC against *E.coli*: was 0.12 µg/mL, MBC was 8 µg/mL, against *S. aureus* MIC and MBC were 2µg/mL.	Jafari et al., 2018
		Compounds isolated from the MeOH extract	Two fold broth microdilution method against ten bacterial strains for determination of the adjuvant effect of the compounds with antibiotics.	Naringenin and rosmarinic acid methyl ester decreased the MIC of ciprofloxacin against *Klebsiella pneumoniae* from 3.125 µM to 0.39 µM. In methicillin- and oxacillin-resistant *Staphylococcus aureus*, combinations with ampicillin lowered the MIC from 50 µM to 12.5 µM. Additionally, naringenin and 5,6-dihydroxy-7,3′,4′-trimethoxyflavone reduced the MIC of gentamicin against *Bacillus subtilis* from 0.125 µM to 0.03125 µM, demonstrating strong antibiotic adjuvant potential.	Alburqan et al., 2025
		EO	Disc diffusion assay against *Listeria monocytogenes* during refrigerated and frozen storage.	Chicken meat inoculated with *L. monocytogenes* (2×10^7 CFU/mL) and treated with the EO (2 µL/mL) showed ~1 log CFU/g reduction, especially within the first 5–7 days at +4 °C. Vacuum packaging enhanced the effect, achieving 1.1–1.3 log CFU/g reductions for up to 14 days at +4 °C and +10 °C, while limited activity was observed at −20 °C (0.1–0.5 log reduction).	Toplu and Önlü, 2025
		EO	*Satureja hortensis* EO (shO) encapsulated in nanoliposomes (shNls) and tested against *Escherichia coli, Pseudomonas aeruginosa, and Staphylococcus aureus* using disc diffussion and micro well dillution assays	ShNls showed strong antibacterial activity against *E. coli* (MIC = 5.187 µg/µL), *P. aeruginosa*, and *S. aureus* (MIC = 2.59 µg/µL), comparable to the free oil. It also reduced DNA damage in human lymphocytes (CBMN assay), indicating genoprotective effects and suggesting a safe and effective antibacterial nanoliposomal formulation.	Cagal et al., 2025
		Nano-liposomal water extracts	Antibacterial activity was evaluated using the broth microdilution MIC method. Antibiofilm activity was assessed using the minimum biofilm inhibitory concentration (MBIC) assay, and the anti-attachment effect was evaluated by measuring *Streptococcus mutans* adherence to glass and enamel surfaces.	Nano-liposomal *S. hortensis* extract showed enhanced antibiofilm and anti-attachment activity against *S. mutans* compared to the aqueous extract, although no significant antibacterial effect was observed; however, its anti-attachment effect on enamel was weaker than chlorhexidine and nano-liposomal *Zataria multiflora*.	Baseri et al., 2025
	S. khuzestanica	EO incorpo-rated in biocomposite films	Agar diffusion and viable cell count methods against *S. aureus* and *E. coli*	In both methods, the films' antibacterial activity against *S. aureus* was higher than that against *E. coli*, and this effect grew as the EO content increased.	Hasheminya et al., 2019

		Nanoemulsified form of EO	measurements of optical density, time killing assay, and the release of cellular components of *E. coli* such as protein, nucleic acid, and potassium	The nanoemulsion significantly disrupted the *E. coli* bacterial membrane, releasing cytoplasmic components and inhibiting bacterial activity within 20 minutes, causing rapid degradation and nearly complete inhibition of bacterial activity.	Alvand, et al., 2021
		EO and carvacrol (**5**)	Disc diffusion, broth microdilution, checkerboard microtiter assay against *E. coli*	MIC of EO was 0.14±0.08 µL/mL, MIC of carvacrol (**5**) 0.09±0.04µL/mL. Significant synergistic effect was found (fractional inhibitory concentration index < 0.05) between the EO and gentamicin.	Mahboubi and Kazempour, 2016
		Nanoemulsion prepared from the EO	Broth microdilution against *E. coli* and *B. atrophaeus*	MIC values against *E. coli* and *B. atrophaeus* were 400 and 1600 µg/mL, respectively. In case of microfluidic system MIC values reduced to 11-50 µg/mL.	Alvand , et al., 2022
		EO	Broth microdilution against oral pathogens	MIC was in the range of 0.015 – 0.25 µL/mL. MBC was in the range of 0.031 – 0.5 µL/mL.	Zomorodian et al., 2015
		O-loaded alginate/CMC and alginate nanoparticle/CC hydrogels	Disc diffusion assay against *Staphylococcus aureus* and *Pseudomonas aeruginosa.*	The nanoparticle hydrogel showed enhanced antibacterial activity, achieving 100% inhibition of *S. aureus* and *P. aeruginosa.*	Saboori et al., 2025
		Nanocomposites of EO	Agar disk diffusion method against *Escherichia coli* and *Staphylococcus aureus* strains.	The inclusion of the EO provided strong antibacterial activity with inhibition zones measuring up to 26 mm.	Rezaei et al., 2025
	S. bachtiarica	EO	Broth microdilution against oral pathogens	MIC was in the range of 0.031 – 0.5 µL/mL. MBC was in the range of 0.062 – 0.5 µL/mL.	Zomorodian et al., 2015
		EO	Broth microdilution method against *E. coli* and *S. aureus*	Against *E.coli* MIC was 0.12 µg/mL while MBC 0.25 µg/mL, against *S. aureus* MIC and MBC were 2 µg/mL.	Jafari et al., 2018
		EO	Disk diffusion method	Inhibition zone diameters (7 – 77 mm) showed a dose-dependent increase against *E. coli*, *S. aureus*, *S. epidermidis*, and *C. albicans*.	Alizadeh, 2016
		n-BuOH extract	An evaluation of the biofilm inhibitory activity of the extract and isolated compounds was made against mature biofilms of *Acinetobacter baumannii, Escherichia coli, Listeria monocytogenes, Pseudomonas aeruginosa*, and *Staphylococcus aureus* using the resazurin microtiter-plate assay.	At 80 µg/mL, biphenyl derivatives significantly inhibited mature biofilms, showing 52.76–75.02% inhibition against *A. baumannii* and *L. monocytogenes*, 69.28% against *E. coli*, 59.38–81.08% against *P. aeruginosa*, and up to 82.94% against *S. aureus.*	Samani et al., 2024
	S. thymbra	Nanovesicles loaded with EO	Microdilution method against *B. cereus, S. aureus, L. monocytogenes, E. coli, P. aeruginosa, S. enterica Serovar Typhimurium*	MIC was in the range from 0.010 to 0.04 µL/mL. MBC was in the range from 0.02 to 0.08 µL/mL.	Vanti et al., 2021
		EO	Broth microdilution method against *Aeromonas veronii* bv. *sobria*	IC_{50} was 43.4 ± 3.9 µg/mL	Mandalakis et al., 2021

		EO from different altitudes and year	Agar-well diffusion, broth dilution method against *S. aureus, B. subtilis, E. coli, P. aeruginosa, P. mirabilis,K. pneumonia, C- albicans*	EO from high altitudes exhibited strong antimicrobial activity with MIC/MLC values of 0.125/0.25 – 0.313/0.375 µg/mL against *S. aureus, P. mirabilis*, *B. subtilis*, *K. pneumonia*, *C. albicans*. EO from lower altitude showed potent activity against *B. subtilis* MIC/MBC 0.188/0.25 µg/mL	Khalil et al., 2020
		EO	Broth microdilution method against nine fish bacterial pathogens	IC_{50} ranged from 26 ± 1 to 92 ± 5 µg/mL–	Anastasiou et al., 2020
	S. intermedia	EO	Microdilution method against oral pathogens	MIC was in the range of 4.2 ± 0.0 – 12.5 ± 0.2 µL/mL. MBC was in the range of 19.4 ± 0.1 – 38.4 ± 0.2µL/mL.	Sharifi-Rad et al., 2015
		EO	Broth microdilution method	MIC was against *E.coli* 0.25 µg/mL, MBC was 0.5 µg/mL, against *S. aureus* MIC and MBC were 4 µg/mL.	Jafari et al., 2018
	S. rechingeri	EO	Disc diffusion method, broth microdilution method against seven bacterial strains compared to standard ampicillin antibiotic.	*S. epidermidis* was the most sensitive (ZOI: 30±0.2 mm, MIC: 0.35 mg/mL) and *K. pneumoniae* was the most resistant (ZOI: 12±0.7 mm, MIC: 7.5 mg/mL) bacterium.	Arman et al., 2022
		Silver nanoparticles loaded with the extract	Microdilution method against *E. coli* and *S. aureus.*	Against *E. coli* MIC was 6.25 µg/mL and against *S. aureus* 1.56 µg/mL.	Narchin et al., 2018
		EO	EO effects on *Listeria monocytogenes* attachment and biofilms were measured by crystal violet and MTT assays.	The EO inhibited *L. monocytogenes* by disrupting biofilms (significantly reducing biomass and metabolic activity), damaging cell membranes and increasing permeability, suppressing motility (complete inhibition at >0.25 µL/mL), and downregulating genes involved in attachment, biofilm formation, and quorum sensing.	Maktabi et al., 2024
	S. kitaibelii different plant parts	EtOH extract	Agar diffusion method against *Micrococcus luteus* and *P. aeruginosa*	The extracts demonstrated antibacterial activity against *Micrococcus luteus* and *Pseudomonas aeruginosa*, with inhibition zones ranging from 20 to 30 mm and 16 to 26 mm, respectively.	Gopčević et al., 2019
		aqueous and EtOAc extracts	Broth microdilution assay	Both extracts were the most active against strains of *Bacillus cereus* and *Micrococcus favus* (MIC 1.70 – 1.99 mg/mL and 1.99 – 3.41 mg/mL, respectively)	Gopčević et al., 2022
		EO	The activity of the EO samples, selected components, and some of their combinations were tested against seven different bacteria and one fungal strain by micro-well dilution assay.	The oils showed MIC values of 160–10,000 µg/mL and MMC values of 630–20,000 µg/mL. Geraniol, borneol, limonene, and p-cymene were the major components, with geraniol showing intrinsic antimicrobial activity (MIC 40–5,000 µg/mL).	Dimitrijević et al., 2024
	S. boissieri	EtOH and aqueous extracts	Disc diffusion assay against *B. subtilis, S. aureus, B. megaterium*, *E. aerogenes, E. coli, K. pneumoniae*, and *P. aeruginosa*.	The EtOH extract showed a strong effect on *B. megaterium* (13.0 ± 1.24 mm inhibition zone).	Aras et al., 2018
	S. laxiflora	EO	Broth microdilution method against *E. coli* and *S. aureus*	Against *E.coli* MIC and MBC were 1 µg/mL, against *S. aureus* MIC and MBC were 0.5 µg/mL	Jafari et al., 2018

	S. cuneifolia	*S. cuneifolia*-extract loaded sodium alginate/polyethylene glycol scaffolds	Agar disk diffusion method against *E. coli* and *S. aureus*	3D printed scaffolds have showed an excellent antibacterial effect, especially against gram-positive bacteria.	Ilhan et al., 2020
	S. mutica	nanofibrous scaffolds loaded with *S. mutica* and *O. decumbens* EO	Colony count method	Loading with 10% of *S. mutica* or *O. decumbens* EO broadened the microbicidal activity of the CS/PVA-PVP/MD scaffolds.	Barzegar et al., 2021
	S. thymbrifolia	EO	The broth microdilution technique was used against seven microbial species.	Broad-spectrum activity against bacteria and fungi was evaluated, and MIC range of 0.05–25 µL/mL was measured. The highest effect was shown against *Candida albicans*.	Jaradat et al., 2025
	S. thymbra<br>*S. parnassica*	EtOAc extract	MIC and MBC of the extracts was determined against eight oral bacteria and *Candida albicans*. Microtiter plate test was conducted to evaluate the antibiofilm activity against *Streptococcus mutans.*	The extract effectively inhibited the growth of obligate anaerobic oral bacteria and demonstrated moderate to high antibiofilm activity against *S. mutans* at concentrations ≥0.15 mg/mL, with effects comparable to chlorhexidine.	Hickl et al., 2024
		Hydroalcoholic extract	MIC and MBC of the extracts against seven oral bacteria and *Candida albicans* was determined.	The extract was effective at concentrations up to 0.6 mg/mL.	Bartels et al., 2025
	S. candidissima	EO	Disk diffusion and microdilution assays were performed against Gram-positive bacteria.	Zones of inhibition ranged from 7 to 37 mm, MIC did not exceed 6.25 µl/ml.	Saidi et al., 2025
	S. calamintha<br>*S. alpina*<br>*S. hortensis*<br>*S. montana*	EO	The antimicrobial activity of the EO as well as its synergism with gentamicin and amphotericin B was evaluated by agar disc diffusion and microwell dilution methods against six pathogenic bacteria and four *Candida* strains.	EOs of Satureja spp. showed antimicrobial activity, with MIC/MBC values of 0.07–36.68 mg/mL (bacteria) and 0.28–9.17 mg/mL (Candida). *S. montana* (0.07–1.14 mg/mL) and *S. hortensis* (0.28–4.46 mg/mL) were the most potent, while *S. calamintha* (17.90–35.80 mg/mL) and *S. alpina* (18.34–36.68 mg/mL) were less active. All oils showed strong synergism with gentamicin against bacteria (4–512-fold MIC reduction) and moderate effects against *Candida* (1–8-fold), with *S. calamintha* also reaching FICI = 0.5 for some strains.	Abbad et al., 2025
	S. macrantha	water, MeOH extracts and EO	Disc diffusion and micro well dillution assays were used.	*S. macrantha* EO was moderate effective (MIC: 125 µg/mL; 6–8 mm), while water and methanol extracts were weak active or inactive (≤6 mm; MIC: 500 µg/mL).	Elmdoustazar et al 2025

Antifungal	*S. kermanica*	nanocomposite incorporated with EO and extract	Inhibition of mycelial growth method	The prepared chitosan nanocomposite displayed enhanced antifungal activity against *Rhizoctonia solani*, *Alternaria alternate*, *Botrytis cinerea*, *Sclerotinia sclerotiorum*, *Fusarium oxysporum*, *Pythium aphanidermatum* strains compared to free EO and the extract.	Payandeh et al, 2022
	S. montana	EO	Microdilution method	EO was more efficient than clotrimazole. MIC value was between 0.0019 – 1% v/v against thirty strains of *C. albicans* from vaginal swab on CHROMagarTM Candida Medium	Bona et al., 2016
		EO	Broth microdilution assay	*S. montana* EO showed strong antifungal activity (MIC 0.9–1.0 g/mL; MFC 1.0–3.0 µg/mL) in comparison to other Lamiaceae EOs against oral isolates of *Candida spp.*	Nikolic et al., 2014
		EO	The fungicidal effect of the EO on stationary phase *Candida albicans* was evaluated by 96-pin replicator. The effect on the hyphae morphology was determined by fluorescent microscopy. FICI values of the EO combined with amphotericin B was determined by checkerboard dilution method.	The EO showed strong antifungal activity. It remained fungicidal at low concentrations (0.25–0.125%). Effectively inhibited stationary-phase cells and hyphal growth, and showed synergy with amphotericin B, especially against stationary-phase infections.	Yuan et al., 2024
		EO	Micro broth dilution assay was made againt *Candida* species. The effect of the EO on biofilm-formation ability were conducted under static conditions in 96-well microplates using the CV method.	All investigated fungi were susceptible to the EO with MIC values ranging from 0.312 to 1.250 mg/mL. Additionally, the oil exhibited a strong antibiofilm activity with a reduction percentage greater than 70%.	Dimitrijević et al., 2025
	S. montana *S. subspicata*	MeOH and EtOH extracts	Broth microdilution assay	Clinical isolates of *Candida spp.* were susceptible to all extracts with MIC values ranging from 3.6 ± 0.1 to 32.9 ± 2.2 µg/mL.	Kremer et al., 2015
	S. thymbra	Nanovesicles loaded with *O. onites* and *S. thymbra* EO	Microdilution method	MICs were in the range from 0.0002 to 0.0080 mg EO/mL of medium. MFCs were from 0.0003 to 0.0080 mg/mL against *A. fumigatus, A. niger, T. viride, P. verrucosum, C. albicans, C. krusei* strains.	Vanti et al., 2021
	S. hortensis	EO and its compounds	Fumigant Antifungal Activity Bioassay against phytopathogenic fungi	Against *Raffaelea querqus-mongolicae* the antifungal activity of the EO was 91.50% at 1.25 mg/paper disc concentration. Against *Rhizoctonia solani* the antifungal activity of the EO was 100% at least 2.5 mg/paper disc concentration. Thymol and carvacrol (5) exhibited the strongest fumigant antifungal activities.	Kim et al., 2019
		EO	Micrrodilution assay, *in vitro* food model system	Against *Aspergillus fumigatus* MIC and MFC values of the EO were both 125 µL/mL.	Sasanian et al., 2018
		EtOH extract	Broth microdilution assay	The studied extract showed higher capacity to inhibit fungal strains *A. fumigatus C. albicans* than bacterial strains	Huwaimel et al., 2023

		EO	Disc diffusion, broth microdilution method	MIC and MFC ranged from 4 to 8 mg/mL against *Alternaria sp., Bipolaris sorokiniana* and *Acremonium sclerotigenum*	Mafakheri and Mirghazanfari, 2018
	S. khuzistanica	Hydrophilic peptide Skh-AMP1	Radial diffusion assay, microdilution assay	MIC ranged from 19.8 to 23.4 µM, and MFC ranged from 39.6 to 58.5 µM against 3 *Aspergillus* and 3 *Candida* species.	Khani et al., 2019
		EO nanoemulsions	Against cucumber powdery mildew using *in vitro* conidia germination assays and greenhouse trials	The nanoemulsion inhibited conidia germination by 83% and reduced disease severity by 54.05% at 3 g/L (up to 72.41% with preventive application).	Abbasi et al., 2025
	S. cilicica	EO	The antifungal potential of the oil against *Sclerotinia sclerotiorum* (Lib.) de Bary, a soil-borne plant pathogen, was evaluated using the mycelial growth inhibition method	EO of *S. cilicica* completely inhibited the growth of fungal mycelia at a dose of 1.0 µL/Petri dish, indicating strong antifungal activity.	Maral et al., 2025
Anthelmintic	*S. thymbra*	EOs of plants from different altitude	On *Allolobophora caliginosa* time of paralysis was recorded when no movement was observed, while time of death when the worms didn't move	EOs showed strong anthelmintic activity against tested earthworm, significantly higher than piperazine citrate.	Khalil et al., 2020
	S. hortensis	70% EtOH extract	On *Ascaris suum* eggs hatching and larval development tests	The extract showed a strong efficacy in egg/larval development inhibition with all tested concentrations.	Băies et al., 2022
	S. montana<br>*S. hortensis*	EO	To assess the in vitro and in vivo anthelmintic potential by Egg Hatch Test, Faecal Egg Count Reduction Test on sheep gastrointestinal nematodes	Inhibition of egg hatchability was 100% at 0.781 – 50 mg/ml concentration for both EO.	Štrbac et al., 2022
	S. hortensis<br>*S. montana*	EO	In vitro and in vivo evaluation of anthelmintic potential against gastrointestinal nematodes: egg hatch test, faecal egg count reduction test	Egg hatch inhibition rate of both EO was 95.3–100%, showing the similar anthelmintic activity to that of thiabendazole (98.0%).	Štrbac et al., 2022
	S. montana	EO	The anthelmintic activity of *Satureja montana* EO was evaluated against sheep gastrointestinal nematodes using *in vitro* egg hatch tests and *in vivo* fecal egg count reduction tests, along with toxicity and coproculture analyses.	The EO showed dose-dependent ovicidal activity (17.0–83.3%, IC_{50} = 0.59 mg/mL) and moderate field efficacy (33–65% egg count reduction) without toxic effects, suggesting potential for integrated parasite control in sheep.	Štrbac et al 2025
Antiparasitic	*S. hortensis*	70% EtOH extract	Anticoccidial activity against *Eimeria spp. Oocysts* isolated from piglets by measuring the percentage of sporulated and destroyed oocysts	After 96 hours of incubation, LC_{50} was 23.99 mg/mL after 96 h of incubation and the percentage of destroyed oocysts was 24.21 ± 2.45% at 5% concentaration after 96 h.	Băies et al., 2023
	S. montana subsp. *montana*<br>*S. montana*	EO	Larvicidal activity and agar penetration assay against *Anisakis simplex* larvae, inhibition of acetylcholinesterase.	*S. montana* subsp. *montana:* The EO caused 100% mortality of *Anisakis* L3 at 1 µL/mL (24–48 h) with LC_{50} values of 151.67–106.34 nL/mL and reduced larval penetration; it also inhibited AChE by 56.72% (IC_{50}: 488.88 nL/mL).	Les et al., 2024

	subsp. *variegata*			*S. montana* subsp. *variegata:* The EO also produced 100% mortality at 1 µL/mL (24–48 h) with LC_{50} values of 222.74–159.78 nL/mL and reduced penetration, while AChE inhibition reached 53.23% (IC_{50}: 872.04 nL/mL).	
	S. montana	EO, carvacrol, p-cymene and γ-terpinene	The nematicidal activity of the EO, its respective dominant volatiles (>5%), their binary combinations, and the reconstituted EO were tested using 96-well microtiter dilution assay against the pinewood nematode.	The EO and carvacrol caused 100% mortality, while p-cymene and γ-terpinene were weakly active (inhibition 16–24%), though their mixture showed synergy (63.7%). The reconstituted EO was most potent (EC_{50} 0.117 mg/mL), stronger than the whole EO (0.151 mg/mL), indicating synergistic interactions among major volatiles.	Pereira and Faria 2024
		EO	The acaricidal efficacy of eight selected EOs was tested on adult mites over 10 days in laboratory conditions using the Petri-dish method, through direct exposure for 1 min (contact toxicity) and subsequent exposure for 1 h (residual toxicity)	The most effective EO in direct exposure-contact after 48 h of observation was *S. montana* (100% toxicity).	Ratajac et al., 2024
Antiprotozoal	*S. khuzestanica*	MeOH extract	Trypan Blue exclusion assay	Against *Trichomonas vaginalis* clinical isolates IC50 measured at 24 and 48 hours were 205.8 and 5.1 µg/mL, respectively. Mortality rate was 100% at 500 µg/mL after 48 hours.	Mahmoudvand et al., 2018
		EO	Promastigotes of *Leishmania major* were treated with EO. The expression of the MDR1 gene was measured using real-time PCR, with GAPDH used as the housekeeping gene for normalization	No significant change in MDR1 expression was observed at 5, 10, or 20 µg/mL compared to the control ($p > 0.05$). However, 15 µg/mL of the EO caused a significant increase in MDR1 gene expression ($p < 0.05$), suggesting that some concentrations may induce drug-resistance mechanisms in the parasite.	Jahanshahi et al., 2024
Antimalarial	*S. mutica*	MeOH extract	Inhibition test of heme detoxification (ITHD)	Showed significant inhibition through plasmodium growth and heme detoxification (>90%)	Mosaddegh et al., 2018
Antioxidant	*S. montana*	MeOH and EtOH extracts collected in seven populations	Radical-scavenging activity, β-carotene bleaching assay, Fe2+ chelating activity	Radical scavenging activity ranged from 31.10 ± 2.58 to 84.05 ± 5.30 µg/mL. Antioxidant activity in β-carotene-linoleate test ranged from 12.59 ± 0.95 to 16.87 ± 0.75 µg/mL. Metal chelating activity ranged from 630.83 ± 52.72 to 3620.23 ± 231.72 µg/mL.	Kremer et al., 2015
		EO	DPPH scavenging and β-carotene bleaching tests	IC50 in the DPPH assay: 200.72 (g/mL), IC50 in the β-carotene bleaching assay: 45.50 (g/mL)	Rezende et al., 2022
		EtOH, MeOH and water extracts	DPPH scavenging test	IC50 for the ethanol extract was 57.3 (µg/mL), for the methanol extract 61.9 (µg/mL) while for the water extract was 113.8 (µg/mL)	Jafri et al., 2023
		extract prepared with high hydrostatic pressure	DPPH, ABTS and FRAP assays	Regarding the antioxidant activity high pressure-assisted extraction was more efficient than atmospheric pressure extraction.	Moreira et al., 2020

		EO	ABTS radical test	IC50 values ranging from 30.02 to 34.5 mg/ml.	Caprioli, etal., 2019
		MeOH, EtOH, and aqueous extracts	DPPH assay	In DPPH assay, the inhibition (%) was 61.16 ± 0.86, 63.76 ± 0.62 and 62.52 ± 1.54 for methanolic, ethanolic, and aqueous extracts, respectively, at 250 µg/mL.	Pavlovic´ et al., 2021
		EO	DPPH, ABTS and FRAP assays	*S. montana* EO showed the highest activity (IC_{50}: 2.685 µL/mL) compared to other medicinal plants.	Raikwar et al., 2025
		EO, water and MeOH extracts	DPPH and ABTS assays	In the $ABTS^{\bullet +}$ assay the water extract showed the highest inhibition (39.763 ± 0.015%), followed by the methanol extract (26.885 ± 0.028%), while the EO exhibited low activity. In the DPPH• assay, the water extract demonstrated the strongest effect (36.289 ± 0.027%), followed by the methanol extract (24.370 ± 0.027%), with no activity detected for the EO.	Coban et al., 2025
		EO	DPPH and ABTS assays	Results. DPPH IC_{50} (1200 µg/mL) and ABTS IC_{50} (500 µg/ml)	Basha et al., 2025
		Water extract	DPPH, ABTS, CURPAC and FRAP scavaging activity	The microwave extract showed stronger radical scavenging activity than the infusion in DPPH (IC_{50}: 2.63 vs 3.73 µg/mL) and ABTS (4.84 vs 6.12 µg/mL) assays. In FRAP, the infusion demonstrated slightly better reducing power (2.64 vs 3.08 mmol Fe/g DE). In CURBAC, both extracts showed comparable activity (554.42 vs 559.33 µg TE/mg DE).	Teofilovi´c et al., 2025
		Dry extract	ORAC, HORAC, and electrochemical methods	The dried extract exhibited considerable antioxidant activity (ORAC=8529.3 µg TE/mg ±159.0, HORAC= 2114.4 µg TE/mg ±17.6 and AOA =25.6 %).	Vilmosh et al., 2024
	S. calamintha spp. *nepeta*	EOs extracted from wild and domesticated *plant*	DPPH and FRAP tests	In the DPPH assay IC_{50} values were between 23.03 – 4.30 and 24.09 – 4.38 µg/mL, while in the FRAP assay, the EC_{50} values were between 55.38 – 2.16 and 60.72 – 7.71 µg/mL for EOs of wild and domesticated plants, respectively.	El Brahimi et al., 2023
	S. boissieri	EtOH and aqueous extracts	DPPH, ABTS assays	DPPH: IC50 were 27.1 ± 1.3 µg/mL and 35.3 ± 1.8 µg/mL for EtOH and water extract, respectively.<br>ABTS: IC_{50} were 22.7 ± 1.1 µg/mL and 51.9 ± 2.3 µg/mL for EtOH and water extract, respectively.	Aras et al., 2018
	S. rechingeri	EO	reducing power and DPPH assays	in the reducing power assay the highest absorption (2.6 nM) was at a concentration of 500 µg/mL, and in the DPPH assay 50% free radical inhibition was found at a 375 µg/mL concentration.	Arman et al., 2022

	S. subspicata	MeOH and EtOH extracts from seven populations	DPPH, β-carotene bleaching, and Fe2+ chelating assays	Radical scavenging activity ranged from 15.65 ± 0.82 to 40.96 ± 2.75 µg/mL. Antioxidant activity in β-Carotene-linoleate test ranged from 13.21 ± 1.66 to 20.88 ± 13.11 µg/mL. Metal chelating activity ranged from 600.39 ± 83.38 to 3052.59 ± 441.68 µg/mL.	Kremer et al., 2015
	S. avromanica	EO and the MeOH extract	DPPH assay	The MeOH extract had the greatest scavenging activity (IC50 21.58 µg/mL), which was followed by the EO (111.34 µg/mL) compared to the IC50 of 22.45 µg/mL for BHT.	Abdali et al., 2017
	S. thymbra	EO, carvacol (**5**), thymol (**7**), p-cymene (**6**), γ-terpinene (**8**)	DPPH assay	IC50: for the EO was 0.22±0.25% (v/v), for carvacrol (**5**) and thymol (**7**) were estimated to be 2.28±0.45 mM and 1.14±0.18 mM, respectively. γ-Terpinene (**8**) and p-cymene (**6**) did not show any significant scavenging activity.	Fitsiou et al., 2016
		EO	Oxygen radical absorbance capacity (ORAC) and CUPric reducing antioxidant capacity (CUPRAC), and ABTS assays	ABTS: 2591 µmol/mL; CUPRAC: 931 µmol/mL, ORAC: 4025 µmol/mL	Anastasiou et al., 2020
	S. parnassica	EO	DPPH assay	IC_{50} was 0.4±0.3% (v/v)	Fitsiou et al., 2016
	S. hortensis	EO	DPPH assay	IC_{50} was 66.85 ± 1.01%	Emre et al., 2021
		EtOH extract	ABTS and hydrogen peroxide radical scavenging assays	IC_{50} in ABTS assay was 2.44 while in the hydrogen peroxide radical scavenging assay was 28.04 µg/mL	Huwaimel et al., 2023
		Extracts prepared by different extraction techniques	Total antioxidant capacity test, inhibition of lipid peroxidation, scavenging of hydroxyl radicals and DPPH assays.	subcritical water extraction resulted the highest antioxidant activity (IC_{50} 23.33 ± 0.48 µg/mL), while Soxhlet extraction, showed the lowest potential (IC_{50}: 51.79 ± 0.47 µg/mL)	Mašković et al., 2017
		MeOH-H_2O extract of plant collected in different phases of vegetation	DPPH assay	Extracts from plants in the budding phase contained the highest levels of compounds with strong radical scavenging activity and antioxidant properties.	Bimbiraite-Survilien et al., 2021
		MeOH extract	DPPH assay	IC_{50} value was 95.52 µg/mL.	Rahimmalek et al., 2020
		EtOH extract	DPPH and FRAP assays	In the DPPH assay, the IC_{50} value was 439.37 µg/mL. In the FRAP assay, the reducing capacity was 24.3%.	Jovanova and Panovska, 2019

		MeOH and dichloro-methane extracts	DPPH assay	Comparing to quercetin (IC_{50} = 26.51 ± 0.06 µg/mL), none of th extracts were effective.	Jafari et al., 2018
		EO	DPPH assay	IC50 value was 4.4 mg/mL.	Shanaida et al., 2018
		EO, water and MeOH extracts	DPPH and ABTS assays	In the ABTS assay (70 µg/mL), the EO showed the highest inhibition (83.973 ± 0.017%), while the methanol extract exhibited moderate activity (37.732 ± 0.219%). In the DPPH• assay (100 µg/mL), the water (20.590 ± 0.002%) and methanol extracts (19.748 ± 0.021%) showed moderate effects, whereas the EO displayed minimal activity (0.646 ± 0.197%).	Coban et al., 2025
		EtOH extracts	DPPH and ABTS assays	The IC_{50} values were in the range of 0.020 to 0.047 mg/mL, while EC_{50} ranged from 0.029 mg/mL to 0.041 mg/mL	Maškovi´c et al., 2024
	S. khuzestanica	biocomposite films incorporated the EO	DPPH assay	Through increase in EO concentration, antioxidant activity increased significantly compared to the control film.	Hasheminya et al., 2019
	S. subspicata	EO, MeOH and water extracts	DPPH and FRAP assay	The MeOH extract has higher antioxidant potential in the DPPH and FRAP assays (IC50 0.45 g/L and 1879.43 µM Fe2+ equiv.) as EO.	Bektašević et al., 2017
	S. mutica	nanofibrous scaffolds loaded with EO	DPPH assay	EO enhanced the antioxidant activity of the scaffolds	Barzegar et al., 2021
	S. bachtiarica	MeOH extract	FRAP test	153.0 ± 5.5 mmol of FeSO4.7H2O equivalent in 100 g of the dried extract	Soodi et al., 2016
		EO obtained by different extraction techniques	DPPH, FRAP tests	IC_{50}s were in the range of 3.04 ± 0.01–3.93 ± 0.011 mg/mL and the results of EC_1 were in the range of 0.38 ± 0.00 to 0.86 ± 0.00 mg/mL	Memarzadeha et al., 2020
		EtOH-water extract	DPPH assay	IC_{50} was ~40 µg/mL compared to BHT (120.48 ± 1.42 µg/mL)	Asadi-Samani et al., 2019
		EO	DPPH, FRAP tests	The IC_{50} values in the DPPH assay ranged from 30.24 to 37.24 mg/mL, while in the FRAP assay varied from 36.45 to 43.24 µM quercetin equivalents per gram of dw.	Alizadeh, 2016
		MeOH extract	DPPH assay	The IC50 value was 114.68 ± 1.67 µg/mL.	Rahimmalek et al., 2020

		EO	DPPH assay	IC_{50} of the EO was 0.095 mg/mL.	Rabiei et al., 2022
		MeOH and dichloro-methane extracts	DPPH assay	Comparing to quercetin (IC50 = 26.51 ± 0.06 µg/mL), none of the extracts demonstrated efficacy	Jafari et al., 2018
		n-BuOH extract	DPPH assay	The antiradical activity of the extract was IC_{50} 85.31 µg/mL, using ascorbic acid as a reference compound (1.35 µg/mL).	Samani et al., 2024
	S. intermedia	MeOH and dichloro-methane extracts	DPPH assay	Comparing to quercetin (IC_{50} = 26.51 ± 0.06 µg/mL), none of the extracts demonstrated efficacy	Jafari et al., 2018
	S. sahendica	MeOH extract	DPPH assay	The IC_{50} value was 374.74 ± 2.58 µg/mL.	Rahimmalek et al., 2020
	S. laxiflora	MeOH and dichloro-methane extracts	DPPH assay	Comparing to quercetin (IC50 = 26.51 ± 0.06 µg/mL), none of the extracts demonstrated efficacy	Jafari et al., 2018
	S. kitaibelii	aqueous and EtOAc extracts	DPPH, ABTS, FRAP and total reducing power (TRP) tests	The aqueous extract from leaves and flowers showed antioxidant activity: SC_{50} value for DPPH was 20 ± 10 µg/mL. ABTS: 2.834±0.02 mg AA/g. FRAP: 1.922±0.03 µmol Fe+2 /mg. TRP: 16.4±1.0.	Gopčević et al., 2022
		EtOH extract	Total antioxidant capacity (TAC), DPPH assay, ferric ion reducing test	DPPH: SC_{50} ranged from 71.20 to 125.65 µg/mL. TAC values ranged from 272.37 to 714.12 mg ascorbic acid equivalents per gram. Ferric ion reducing antioxidant power ranged from 0.74 to 1.94 µmol Fe/mg.	Gopčević et al., 2019
	S. pilosa	Dichloromethane, acetone and MeOH extracts	DPPH, ABTS, and CUPRAC assays	Cu^{2+} reducing (CUPRAC, mmol TR/g): Leaf methanol extract = 2.40 ± 0.07; leaf DCM = 1.13 ± 0.04; branch DCM decreased ~50% vs leaf; branch acetone extract had the highest activity.<br>Radical scavenging: DCM extracts (leaves and branches) had the lowest activity; acetone extract showed the highest $ABTS^{\cdot+}$ DPPH scavenging.	Kinoglu et al., 2024
	S. macrantha<br>*S. hortensis*	EO, MeOH and water extracts	ABTS and DPPH assays	At 50 µg/mL, *S. macrantha* showed higher antioxidant activity than *S. hortensis*, with extracts more active than EOs. In $ABTS^{\cdot+}$, *S. macrantha* water extract was highest (52.1%), while *S. hortensis* showed lower activity (methanol: 17.6%; EO: 10.7%). In $DPPH^{\cdot}$, *S. macrantha* remained higher (28.8%; EO: 10.1%), whereas *S. hortensis* showed weak activity (4.4%; EO: 4.3%).	Elmdoustazar et al., 2025
	S. cilicica	EO	DPPH assay	The EO showed low antioxidant activity, weak DPPH radical scavenging capacity (IC_{50} = 39.884 $mg \cdot mL^{-1}$)	Maral et al., 2025

	S. spicigera *S. aintabensis*	MeOH extract	DPPH, DMPD, ABTS, FRAP, CUPRAC assays	*S. aintabensis* showed high antioxidant activity with Fe^{3+} (0.597) and Cu^{2+} (1.016) reducing power, strong ABTS (IC_{50}: 8.77 µg/mL), moderate DPPH (13.07 µg/mL), and weak DMPD (30.13 µg/mL). *S. spicigera* showed moderate activity, with lower Fe^{3+} (0.421) and similar DPPH (12.37 µg/mL) and ABTS (9.49 µg/mL), but weak DMPD (33.00 µg/mL).	Yilziz et al., 2025
	S. thymbrifolia	EO	DPPH assay	The EO showed a strong anti-DPPH radical activity (IC_{50}=15.84 ±0.11 µg/mL) compared to Trolox (IC_{50}=2.88 ± 0.57 µg/mL).	Jaradat et al., 2025
	S. hasturkii	DCM, EtOAc, EtOH extracts and EO	DPPH, AChE, BChE, TYR, elastase, collagenase, XO inhibitory, and metal chelating activity	The extracts exhibited low to moderate enzyme inhibition at 2 mg/mL. The EtOAc extract showed the highest elastase (16.70 ± 2.08%) and collagenase (13.96 ± 3.70%) inhibition. XO inhibition was strongest with the EtOH extract (34.34 ± 0.60%), followed by EtOAc (30.32 ± 2.03%). The DCM extract demonstrated the highest metal-chelating activity (72.38 ± 1.86%)	Buyukyildirim et al., 2025
	S. barceloi different parts	MeOH, EtOAc, hexan, and water extracts	ABTS and DPPH assays	Antioxidant activity of *S. barceloi* varied significantly by solvent and plant organ. Methanolic extracts showed the highest activity, particularly stems (DPPH: 50.5 mg TE/g Ex) and leaves (DPPH: 43.77 mg TE/g Ex; ABTS: 84.91 mg TE/g Ex), while hexane extracts of flowers and roots showed the lowest activity in both DPPH (0.36–0.77 mg TE/g Ex) and ABTS (0.97–1.55 mg TE/g Ex) assays.	Raadani et al., 2024
	S. kermanica	Nanoemulsion of the MeOH extract	DPPH scavenging activity	The methanolic extract showed an IC_{50} of 30.105 ± 2.781 µg/mL in the free radical scavenging assay, while the nanoemulsion showed an IC_{50} of 58.14 ± 0.84 µg/mL.	Hassanabadi et al., 2024
	Satureja fatty acid of *S. hortensis, S. rechingeri, S. sahendica, S. bakhtiarica, S. khozestanica* and *S. mutica*	Fatty acids	ABTS	*Satureja* fatty acid displayed strong antioxidant capacity (with IC_{50} ranging from 354 to 428 µg/mL).	Obeidnejad et al., 2024
	S. candidissima	EO	DPPH scavenging activity, β-carotene bleaching	The EO showed low antioxidant activity, with weak DPPH radical scavenging capacity (IC_{50} = 9.36 mg·mL^{-1}). Consistently, in the β-carotene–linoleic acid bleaching assay, it exhibited low inhibition (13.92%).	Krimat et al., 2025
Modification of antioxidant enzymes	*S. khuzestanica*	EO	Levels of glutathione (GSH), activity of CAT, SOD, GPX, was determined with the adequate kits in alloxan-induced Type 1 diabetic rat	Diabetic animals treated with the EO significantly inhibited the decrease of GSH, SOD, GPX, and CAT activity compared to untreated animals, with a reduction of 47.7%, 19.92%, 21.15%, and 39.34%, respectively.	Ahmadvand, 2014

Antitumor	*S. thymbra* and *S. parnassica*	EO carvacrol (**5**), thymol (**7**), p-cymene (**6**), γ-terpinene (**8**)	Sulforhodamine B assay for antiproliferative activity	Against MCF-7 cells: EC_{50} of the EO (*S. thymbra*) was 0.002±0.00038% (v/v), and EO (*S. parnassica*) 0.08±0.03% (v/v), for γ-terpinene (**8**) was 4.6±0.11 mM and for p-cymene (**6**) 5.11±0.5 mM. Against A549 cells EC_{50} of carvacrol (**5**) was 0.118±0.0012. EC_{50} of thymol (**7**) was 0.181±0.016 mM against Hep3B. γ-Terpinene (**8**) and p-cymene (**6**) had limited bioactivity.	Fitsiou et al., 2016
	S. cuneifolia	EO and EtOH extract	MTT assay	The EOs showed notable cytotoxic activity against A549 cells.	Yıldız et al., 2023
	S. montana	Hexane, dichloro-methane and butanol extracts	MTT assay	Hexane and dichloromethane fractions showed IC_{50} values ranging from 32.1 to 47.8 µg/ml for K562 cells and 44.3 to 45.7 µg/ml for Jurkat cells. They induced apoptosis, increased cell death in the sub-G1 phase, and significantly increased caspase-3 activity in both cell lines.	Asadipour and Amirghofran, 2019
		EO	MTT assay	The EO displayed cytotoxic activity with IC_{50} values ranging from 40.13 to 65.51 µg/mL.	Nikolic et al., 2014
		EtOH and supercritical CO_2 extract	*In vivo* model of Ehrlich ascites carcinoma (EAC) in mice, and redox status of malignant cells	The volume of the ascites significantly decreased. The extracts induced oxidative stress in malignant cells. Pretreatment and treatment increased xanthine oxidase activity, lipid peroxidation, and decreased catalase activity.	Vladić et al., 2020
		MeOH, EtOH, and aqueous extracts	MTT, NBT, Griess, and comet assays	All extracts exhibited genoprotective potential on plasmid DNA. The antitumor effects were associated with the modulation of ROS and NO production, which led to genotoxic effects on tumor cells and a decrease in their proliferation.	Pavlovic´ et al., 2021
		EO	MTT assay	Cytotoxicity testing showed low activity with IC_{50} values of 1.27 mg/mL in endometrial (BEND) cells and IC_{50} 1.02–1.56 mg/mL in kidney (MDBK) cells across exposure times.	Ratajac et al., 2025
	S. intermedia	EO	Crystal violet staining method	The EO exhibited concentration-dependent effects on the viability of Hep-G2 and MCF-7 cell lines, ($p < 0.05$)	Sharifi-Rad et al., 2015
	S. thymbra	EOs from plants of different altitude	Sulforhodamine B assay	EO of plants from high altitude displayed a notably better cytotoxic effect, particularly against HCT-116 cells, with an IC_{50} value of 2.45 ± 0.21 µg/mL than low altitude plants.	Khalil et al., 2020
	S. hortensis	EtOH extract	MTT assay, annexin V-FITC assay, cell cycle analysis by CycleTESTTM PLUS DNA Reagent Kit.	The extract showed IC_{50} value of 113.05 µg/mL against-549 cell line. Apoptosis and cell cycle studies for this cell line revealed that the cell cycle was halted at S-phase.	Huwaimel et al., 2023
		EtOH extract prepared by different extraction methods	MTT assay for determination of the cytotoxic activity	The IC_{50} values for Hep2c, RD, I2OB cell lines were found to be 13.23–35.29, 18.43–31.03, and 20.51–34.09 µg/mL, respectively. subcritical water extraction extracts had the lowest IC_{50} values.	Mašković et al., 2017

		MeOH extract	MTT assay for determination of the cytotoxic activity	The extract was effective against K562 cell line with a IC_{50} value of 52 μg/mL and against Jurkat cells with IC_{50} 66.7 μg/mL.	Esmaeilbeig, et al., 2015
		EO and MeOH extract	Potato disc tumor inhibition method.	The MeOH extract showed antitumor activity,with an IC_{50} value of 1.8 mg/ml, and the EO showed 100% tumor growth inhibition at at dilutions of 0.001 and 0.0001 (v/v).	Ramezani et al., 2016
		MeOH-H_2O extract	MTT test for assay cells viability	IC_{50} of the extracts from different vegetation phases were between 25 μg/mL to 8.535 mg/mL for melanoma cells.	Bimbiraite-Survilien et al., 2021
		MeOH extract	The cytokinesis-block micronucleus cytome assay, RT-MLPA apoptosis assay and the mice reticulocytes micronucleus assay	Minor genotoxic potential at the tested concentrations, but no cytostatic or cytotoxic effects *in vitro*. At 200 mg/kg per os extracts decreased reticulocyte micronuclei frequency in mice, and downregulated pro-apoptotic and anti-apoptotic genes.	Čakar et al., 2018
	S. subspicata	silver nanoparticles loaded with aqueous extract	MTT assay	The most significant cytotoxicity was observed at a concentration of 100 μg/mL on HT-29 cells, with lower toxicity reported on HEK-293 cell.	Narchin et al., 2018
	S. rechingeri	EtOH-water extract	MTT assay.	The extract was inactive against CF7 and MDA-MB231 cell lines, IC_{50} values were above 300 μg/mL.	Asadi-Samani et al., 2019
	S. bachtiarica	MeOH extract	MTT assay	The extract was effective against K562 cell line with a IC_{50} value of 28 μg/mL and against Jurkat cells with IC_{50} 125 μg/mL. The extract had less effect on Raji, Fen, and HeLa cell lines.	Esmaeilbeig, et al., 2015
	S. khuzistanica	Silver nanoparticles from the aqueous extract	MTT assay, real-time PCR, and annexin V/PI assay	A cell viability reduction, a decrease in the expression of the anti-apoptotic gene bcl-2, an increase in the mRNA expression ratio of bax gene and a significant increase in the apoptotic index, bax/bcl-2 expression ratio was observed.	Mohammadi-Ziveh et al, 2020
		extract	MTT assay, and the expression of BAX, BCL2, SMAC, P53, MMP9, CASP9, and SUR genes was analyzed using real time PCR.	Increase was observed for *S. khuzistanica* extract in the expression of BAX (4.21-fold), SMAC (3.055-fold), P53 (5.105-fold), and CASP9 (2.216-fold) ($p < 0.0001$) compared to the control. In contrast, BCL2, MMP2, SUR, and MMP9 expression decreased by 1.1% relative to the control. Combined treatment with *S. khuzistanica* and nisin or doxorubicin produced greater changes in gene expression than the extract treatment alone.	Kheiri et al., 2025
		Skh-AMP1 peptide	MTT assay	At 25.2 µM the peptide exhibits 3.6% cytotoxicity after 48 hours on HEK293 cells.	Khani et al., 2019
	S. isophylla different parts	EO	MTT assay	The EOs showed their strongest effect against MCF-7 cells (human breast adenocarcinoma) (48.64% inhibition for stem oil; 22.14% for flower oil), while only mild activity was observed on PC-12 (adrenal	Aghaaliakbari et al., 2024

				phaeochromocytoma) and AsPC-1 cells (human pancreatic cancer) (inhibition ≈5–8%).	
	S. thymbrifolia	EO	MTT assay	IC_{50} values: 192.15 µg/mL (CaCo-2), 194.80 µg/mL (HeLa)	Jaradat et al., 2025
	S. kermanica	Nanoemulsion of the MeOH extract	MTT assay	In the MCF-7 breast cancer cell line, the IC_{50} was 1251.088 µg/mL for the methanolic extract and 201.745 ± 4.554 µg/mL for the nanoemulsion.	Hassanabadi et al., 2024
	Fatty acid from *S. hortensis, S. rechingeri, S. sahendica, S. bachtiarica, S. khuzistanica* and *S. mutica*	Fatty acids	MTT assay	The fatty acids from *Satureja* species reduced the viability of hematopoietic mouse macrophage cells in a concentration-dependent manner. The cytotoxicity rapidly increased at doses greater than 0.12 mg/mL.	Obeidnejad et al. 2024
Protective activity against the side effects of chemotherapy	*S. khuzistanica*	EO	busulfan-induced damage on testis in male mice, MTT assay, TUNEL assay	pretreatment and cotreatment with EO decreased cytotoxic effects of busulfan and cause the improvement of sperm parameters during experimental chemotherapy.	Nasimi et al., 2016
	S. montana	80% ethanol + 1% HCl extract	Determination of oxidative stress markers, serum testosterone, LH and FSH levels, testicular and apoptotic markers, and apoptosis-related parameters in rats. Histopathological studies.	Protective effect of *S. montana* extract in testicular toxicity was demonstrated: testicular weight, serum testosterone levels, and alkaline phosphatase activity was restored, lipid peroxidation decreased, antioxidant capacity improved, testicular DNA fragmentation mitigated, gene expression, and up-regulated PPAR-c and Akt1 levels decreased.	Abd El Tawab et al., 2014
	S. hortensis	mucoadhesive gel containing 1% extract	Double-blind clinical trial on 60 children who were affected by mucositis following chemotherapy	Pain severity reduced from 3.5±2.1 to 0.0 ± 0.0 in *S. hortensis* group and 3.1 ± 1.8 to 0.4 ± 1.0 in control group in fifth day ($p < 0.001$).	Bostanabad et al., 2018
		MeOH extract	Assessing tissue morphology, ameliorated levels of serum parameters for liver, renal and testes function, tissue oxidative stress parameters, and increased Bcl-2/Bax ratio in Wistar rats.	Treatment with the extract improved kidney, liver, testicular tissue structure, serum parameters, reduced oxidative stress, and improved the Bcl-2/Bax ratio, suggesting a protective effect against cell damage caused by cisplatin.	Boroja et al., 2018
Effects on fatty liver syndrome	*S. khuzistanica*	EO	EO was administrated via oral gavage to 250 male broiler chickens	The administration of the EO reduced the liver fat in broiler chickens, but the study did not identify a consistent effective dosage for all ages.	Mirderikvandi, et al., 2020
Hemolytic	*S. khuzistanica*	Skh-AMP1 peptide	Haemolytic activity on human erythrocytes	Skh-AMP1 showed hemolytic activity for human erythrocytes ranging from 0.98-2.1% at concentrations 36-72 µM, and low activity at MIC 25.2 µM.	Khani et al., 2019

Anti-inflammatory	*S. hortensis*	Dicloro-methane, butanol and hexan extracts	Lipopolysaccharide -activated J774.1 macrophages were treated with extracts, and the expression and release of various inflammatory factors by macrophages were examined.	The extracts significantly decreased gene expression of various proteins, including iNOS (<0.44 fold of control), COX-2 (<0.29 fold) , IL-1β (<0.41 fold), IL-6 (<0.25 fold), and TNF-α (<0.2 fold), and reduced macrophage protein production.	Farzaneh et al., 2018
	S. montana	EO	The inhibitory effects was measured on the release of the cytokine tumor necrosis factor-α (TNF-α) and the chemokine (C-C motif) ligand 2 (CCL2) in human acute monocytic leukemia cells stimulated by LPS.	The EO demonstrated toxicity to the chemokine (C-C motif) ligand 2 (CCL2) at the lowest tested concentration (3 μg/mL).	Miguel et al., 2020
		Dry extract	Immunomodulatory activity was evaluated in acute and chronic stress models by measuring the serum levels of cytokines TNF-α, IL-6, and IL-1β in a cohort of 112 male 8-week-old Wistar rats.	A dose of 250 mg/kg of *S. montana* significantly decreased IL-6 in comparison to carvacrol and significantly reduced TNF-α and IL-6 in comparison to rosmarinic acid.	Vilmos et al., 2024
	S. khuzistanica	EO	Evaluation of intracranial pressure, neurological outcomes, brain cytokines and astrocytic and neuronal activation in male Wistar rats induced with traumatic brain injury	A concentration of 200 mg/kg administered 30 min, significantly reduced brain oedema, blood-brain barrier damage, and intracranial pressure increase, improving veterinary coma scores, reducing inflammatory markers, and affecting astrocytic activation.	Abbasloo et al., 2016
	Fatty acids from *S. hortensis, S. rechingeri, S. sahendica, S. bachtiarica, S. khuzistanica* and *S. mutica*	Fatty acids from	Hydrogen peroxide, NOX, NF-kB, and NRF2 in lipopolysaccharide-stimulated macrophage through Nrf2/NF-kB/ NADH oxidase pathway were meased.	Fatty acids of *Satureja* species reduced NOX and NF-κB expression, hydrogen peroxide production, and increased NRF2 expression, indicating antioxidant and anti-inflammatory effects mediated through the Nrf2/NF-κB pathway at 0.04 mg/mL concentration.	Obeidnejad et al., 2024
Preventing opioid analgesic tolerance	*S. khuzistanica*	EtOH extract	Measuring the reversal in spinal cord changes (GFAP and TNFa levels) in adult male Wistar rats who developed tolerance to morphine	Extract prevented the development of morphine tolerance in rats when combined with morphine. It also increased levels of glial fibrillary acidic protein (GFAP) and tumor necrosis factor alpha (TNFα) in the spinal cord, which were reversed with 100 mg/kg extract.	Esmaeili-Mahani et al., 2015
Antiepileptic	*S. bachtiarica*	EO	Measuring latency to the first seizure, head tic frequency, total frequency of the head and upper limbs seizures, frequency of tonic seizures, frequency of repeated spin and jump, survival rate against PTZ-induced seizure in mice	Administering the EO to PTZ-treated mice increased latency to first seizure, survival, and decreased frequency of head and upper limb seizures, total body seizures, tonic seizures, and jumping.	Rabiei et al., 2022
Analgesic	*S. hortensis*	EO	Hot-plate test and the tail-flick test	Significant increase in the response times was observed. The onset of its analgesic effects was 60 min post-treatment in the hot-plate test and 90 minutes post-treatment in the tail-flick test.	Asghari et al., 2018

Improve memory impairment	*S. bachtiarica*	MeOH extract	Frap assay, in vitro AChE activity, behavioral tests, immunohistochemical evaluation, measurement of brain AChE activity and lipid peroxidation	The extract attenuated Aβ-induced memory deficits, reduced cholinergic neurons in the hippocampus, mitigated Aβ-induced lipid peroxidation, and increased acetylcholinesterase activity.	Soodi et al., 2016
	S. hortensis *S. macrantha*	EO, water and MeOH extracts	Acetylcholinesterase (AChE) and butyrylcholinesterase (BChE) inhibitory assays.	*S. hortensis* showed slightly higher AChE inhibition (methanol extract: 16.05%; EO: 14.41%; water extract: 6.56%) than *S. macrantha* (methanol: 12.72%; water: 9.80%; EO: 7.84%). For BChE inhibition, *S. hortensis* exhibited stronger activity (EO 35.68% and methanol extract 28.01 % inhibition), while the water extract was inactive. In comparison, *S. macrantha* showed moderate BChE inhibition (methanol: 22.33%) and lower activity of the water extract (9.86%) and EO (10.45%).	Elmdoustazar et al., 2025
	S. aintabensis *S. spicigera*	MeOH extracts	Acetylcholinesterase (AChE) and butyrylcholinesterase (BChE) inhibition assays	*S. aintabensis* showed moderate inhibition of AChE (31.8 ± 2.2%) at 40 μg/mL, while exhibiting weak inhibition of BChE (9.4 ± 1.4%). *S. spicigera* demonstrated slightly higher AChE inhibition (39.7 ± 1.6%) but very weak BChE inhibition (1.3 ± 0.7%) at the same concentration.	Yilziz et al., 2025
	S. barceloi different parts	MeOH, EtOAc, hexane and water extracts	Acetylcholinesterase (AChE) and butyrylcholinesterase (BChE) inhibition assays	Strongest inhibition was shown by the methanolic leaf extract (0.224 mg ED/g) and methanolic stem extract (0.219 mg ED/g). The weakest inhibition was found for methanolic root and ethyl acetate leaf extracts (0.01 mg DE/g).	Raadani et al., 2024
	S. pilosa	DCM, acetone and MeOH extracts	Acetylcholinesterase (AChE) and butyrylcholinesterase (BChE) inhibition assays	The leaf methanol extract showed the strongest AChE inhibition with an IC_{50} of 41.2 ± 5.6 μg/mL, while the leaf dichloromethane extract exhibited the highest BChE inhibition with an IC_{50} of 52.3 ± 8.6 μg/mL.	Kinoglu et al., 2024
Immunostimulant	*S. khuzestanica*	EtOH extract	Lysozyme activity, antibody titers, complement activity, and bactericidal activity in the serum of the fish.	The extract enhance only some immunity indices including lysozyme and bactericidal activity in non-vaccinated fish.	Alishahi et al., 2015
Antiglycation	*S. hortensis, S. bachtiarica, S. sahendica*	MeOH extract	Determination of protein glycation and AGEs formation capacity, Congo red binding assay	The highest and lowest anti-glycative activities showed *S. hortensis* and *S. sahendica*, respectively, following the anti-AGE order: *S. hortensis* > *S. bachtiarica* > *S. sahendica.*	Rahimmalek et al., 2020
Prebiotic	*S. hortensis*	EO emulsion	Humanized gnotobiotic mouse model, microbiota analysis by 16S rRNA gene sequencing, determination L-carnitine, TMA and TMAO levels in plasma	Gut microbiota induced by EO as prebiotics could regulate cardiovascular and metabolic factors	Sánchez-Quintero et al., 2022
Antispasmodic and antidiarrheal	*S. montana*	EO	The spasmolytic activity was assesed on isolated rat ileum function. The in vivo antidiarrheal activity was evaluated in young and adult rats.	The EO showed ex vivo spasmolytic activity in isolated rat ileum. It produced an in vivo anti-diarrheal effect in young rats, and EO-treated groups showed a significant reduction in castor oil-induced diarrhea compared to control group.	Kulić et al., 2023

Anti-diabetic	*S. hortesis*<br>*S. macrantha*	MeOH extract and EO	α-Glucosidase and α-amylase enzyme inhibition	*S. macrantha* exhibited notable α-glucosidase inhibition at 1000 µg/mL, with water (49.4%) and methanol (39.4%) extracts exceeding acarbose (34.98%), while its EO was inactive; no α-amylase inhibition was observed. In contrast, *S. hortensis* showed no α-glucosidase activity, and only its EO displayed moderate α-amylase inhibition (41.33%), below acarbose (64.5%).	Elmdoustazar et al., 2025
	S. hortensis<br>*S. montana*	EO, water and MeOH extracts	α-Glucosidase, α-amylase, butyrylcholinesterase and acetylcholinesterase enzyme inhibition	*S. montana* showed no α-amylase inhibition (1–5000 µg/mL); at 1000 µg/mL, its EO exhibited the highest BChE inhibition (54.71 ± 1.82%), followed by the methanol extract (37.58%). In contrast, *S. hortensis* displayed strong α-glucosidase inhibition at 5000 µg/mL (methanol 69.84%, water 49.45%), with no α-amylase activity. In cholinesterase assays (1000 µg/mL), its EO showed the highest AChE inhibition (31.85 ± 0.39%) and notable BChE inhibition (40.20%).	Coban et al., 2025
	Different parts of *S. isophylla*	EO	Butyrylcholinesterase and acetylcholinesterase enzyme inhibition	The EOs showed below-average inhibitory activity against AChE (IC_{50} > 500 µg/mL), while exhibited strong inhibition of BChE, with IC_{50} values ranging from 68.08 ± 1.12 to 110.88 ± 1.34 µg/mL.	Aghaaliakbari et al., 2024
	S. khuzestanica	Capsulated ground dried leaves	In this double-blind, placebo-controlled RCT, 78 T2DM patients (39 per group) received 500 mg/day *S. khuzestanica* (SK) or placebo for 12 weeks. Anthropometric, blood pressure, liver, glycemic, and lipid parameters were measured pre- and post-intervention.	SK supplementation significantly improved glycemic and lipid parameters compared to placebo. It reduced FBS (−12.6 vs +3.5 mg/dL; p = 0.007), HbA1c (−0.28% vs +0.11%; p < 0.001), insulin (−1.65 vs +2.09 mIU/L; p = 0.03), total cholesterol (−14.6 vs +8.2 mg/dL; p < 0.001), and LDL (−4.6 vs +5.8 mg/dL; p < 0.001), while increasing HDL (+3.9 vs +0.9 mg/dL; p = 0.005).	Roosta et al., 2024
	Fatty acid from *S. hortensis, S. rechingeri, S. sahendica, S. bachtiarica, S. khuzestanica* and *S. mutica*	Fatty acids	Antilipase and antiamylase activities.	*Satureja* fatty acid displayed strong anti-lipase capacity (IC_{50} ranging from 354 to 428 µg/mL), and anti-amylase capacity (IC_{50} ranging from 370 to 390 µg/mL).	Obeidnejad et al., 2024
	Different parts of *S. barceloi*	MeOH, EtOAc, hexane and water extracts	Measuring α-amylase inhibitory activity	All extracts inhibited α-amylase, reducing potential carbohydrate absorption. The highest inhibition showed the aqueous leaf (305.61 mg EA/g Ex), stem (257.36 mg EA/g Ex), flower extract (249.14 mg EA/g Ex); lowest inhibition the hexane leaf extract (2.53 mg EA/g Ex).	Raadani et al., 2024
Cardioprotective	*S. hortensis*	Hydroalcoholic extract	Rats were orally pretreated for 30 days with the extract (200 or 400 mg/kg) or metoprolol (10 mg/kg), followed by two subcutaneous doses of isoproterenol (ISO) to induce myocardial infarction. Serum cardiac markers—creatine kinase-MB (CK-	Pretreatment with 400 mg/kg of the extract significantly (P < 0.001) prevented ISO-induced increases in CK-MB, cTnI, LDH, AST, and ALT, improved ECG patterns, reduced oxidative stress markers, and preserved myocardial structure. The extract effectively protected	Muthukumar et al., 2024

			MB), troponin I (cTnI), alanine transaminase (ALT), aspartate transaminase (AST), and lactate dehydrogenase (LDH)—electrocardiogram (ECG), histopathology, malondialdehyde (MDA) levels, and antioxidant enzymes were evaluated.	against both biochemical and histological changes associated with myocardial infarction.	
Wound-healing activities	*S. montana*	Hydroxypropyl-β-cyclodextrin glycerol-assisted extract	DPPH antiradical activity, β-carotene-linoleic acid, anti-lipoxygenase, anti-heat-induced ovalbumin coagulation, and UV absorbance assays.	The extracts showed strong antioxidant and cosmeceutical activities, surpassing standards in multiple assays. They maintained >80% HaCaT cell viability and significantly improved wound closure (~48.6% vs. 34.8% control), indicating high biocompatibility and potential for direct cosmetic application.	Jakupovic et al., 2025
		Hydrolate	Full-thickness burn wounds were induced in rabbits and treated with hydrolates of *Satureja montana* or *Origanum vulgare,* then redox (MDA, SOD, GSH), inflammatory cytokines (TNF-α, IL-1, IL-10), and histological healing markers were evaluated over 14 days.	Both hydrolates improved oxidative and inflammatory status in burn wounds, reducing MDA and $CD45^+$ cells and restoring antioxidant levels, with *S. montana* showing the strongest modulation of cytokines and overall wound-healing effects.	Demyashkin et al., 2025
Organ protection against heavy metal damage	*S. hortensis*	EO	The EO was evaluated for its protective effect against lead acetate–induced multi-organ toxicity in rats by administering two doses for 10 days and assessing behavioral tests, ECG, biochemical markers, and histopathology.	The high dose significantly improved cardiac activity, behavior, and locomotion while reducing inflammatory and oxidative stress markers and improving liver, kidney, and heart function, indicating protective effects against lead-induced organ damage.	Aboubaker et al., 2025

References

Abbad, I.; Soulaimani, B.; Iriti, M.; Barakate, M. Chemical Composition and Synergistic Antimicrobial Effects of Essential Oils From Four Commonly Used Satureja Species in Combination With Two Conventional Antibiotics. *Chem. Biodivers*. **2025**, *22*, e202402093.

Abbasi, A.T.; Ebrahimi, L.; Farzaneh, M. Antifungal efficacy of plant essential oil nanoemulsions against cucumber powdery mildew. *Sci. Rep.* **2025**, *15*, 40291.

Abbasloo, E.; Dehghan, F.; Khaksari, M.; Najafipour, H.; Vahidi, R.; Dabiri, S.; Sepehri, G.; Asadikaram, G. The anti-inflammatory properties of *Satureja khuzistanica* Jamzad EO attenuate the effects of traumatic brain injuries in rats. *Sci. Rep.* **2016**, *6*, 31866.

Abd El Tawab, A.M.; Shahin, N.N.; AbdelMohsen, M.M. Protective effect of *Satureja montana* extract on cyclophosphamide-induced testicular injury in rats. *Chem. Biol. Interact.* **2014**, *224*, 196-205.

Abdali, E.; Javadi, S.; Akhgari, M.; Hosseini, S.; Dastan, D. Chemical composition and biological properties of *Satureja avromanica* Maroofi. J. *Food. Sci. Technol.* **2017**, *54*, 727–734.

Aboubaker, D. H.; Shaffie, N. A.; Shabana, M. F.; Abd Elghafour, A.; Ibrahim, B. M. M. Protective role of savory essential oil on vital organs in rats against deleterious effects induced by lead acetate. *Biotechnol. Rep.* **2025**, *45*, e00871.

Aghaaliakbari, B.; Mojtahedi, M.M.; Hajiaghaee, R.; Abaee, M.S.; Besati, M.; Ghafarzadegan, R.; Tavakoli, S. Chemical composition, Cholinesterase inhibitory effect and Cytotoxic activity study of essential oils extracted from different parts of *Satureja isophylla* Rech. f. *Jundishapur J. Nat. Pharm. Prod.* **2024**, *19*, 19.

Ahmadvand, H. Amelioration of altered antioxidant enzyme activity by Satureja khuzistanica essential oil in alloxan-induced diabetic rats. *Chin. J. Nat. Med*. **2014**, *12*, 9, 672-676.

Alburqan, M.; Kincses, A.; Paizs, M.; Barta, A.; Veres, K.; Csámpai, A.; Yazdani, M.; Hohmann, J. Adjuvant potential of *Satureja hortensis* metabolites with antibiotics against Gram-positive and Gram-negative bacterial strains. *Pharm. Biol.* **2025**, *63*, 716-729.

Alishahi, M.; Halimi, M.; Khansari, A.; Yavari, V. Extracts of *Oliviera decumbens* and *Satureja* khuzestanica as immunostimulants affect some innate immunity indices of *Cyprinus carpio* against *Aeromonas hydrophila* infection. *Aquac. Res*. **2015**, *47*, 2909-2916.

Alizadeh, A. Essential oil constituents and biological activities of different ecotypes of *Satureja bachtiarica* Bunge. as a traditional herbal drug in southwestern*, Iran. J. Essent. Oil. Bearing. Plant.* **2016**, *19*, 1328-1339.

Alvand, Z.M.; Rahimi, M.; Rafati, H. A microfluidic chip for visual investigation of the interaction of nanoemulsion of *Satureja Khuzistanica* essential oil and a model gram-negative bacteria. *Int. J. Pharm.* **2021**, *607*, 121032.

Alvand, Z.M.; Rahimi, M.; Rafati, H. Interaction of a natural compound nanoemulsion with Gram negative and Gram positive bacterial membrane; a mechanism based study using a microfluidic chip and DESI technique. *Int. J. Pharm.* **2022** *626*, 122181.

Anastasiou, T.I.; Mandalakis, M.; Krigas, N.; Vézignol, T.; Lazari, D.; Katharios, P.; Dailianis, T.; Antonopoulou, E. Comparative Evaluation of essential oils from Medicinal-Aromatic Plants of Greece: Chemical Composition, Antioxidant Capacity and Antimicrobial Activity against Bacterial Fish Pathogens. *Molecules* **2020**, *25*, 148.

Aras, A.; Bursal, E.; Alan, Y.; Turkan, F.; Alkan, H.; Kılıç, Ö. Polyphenolic Content, Antioxidant Potential and Antimicrobial Activity of *Satureja boissieri*. *Iran. J. Chem. Chem. Eng.* **2018**, *37*,6, 209-219.

Arman, M.; Pirian, K.; Alinaghizadeh, M.; Khosheghbal, F.; Nahavandi, R.; Jahromi, S.T. Study of compounds, cytotoxicity and biological activities of essential oil of *Satureja rechingeri* Jamzad. *Adv. Trad. Med.* **2022**, *22*, 789-796.

Asadipour, M.; Amirghofran, Z. *Satureja hortensis* induces cell death and inhibited cell cycle progression in K562 myelogenous and Jurkat T cell leukemia cell lines. *J. Immunoassay. Immunochem.* **2019**, *40*, 459–472.

Asadi-Samani, M.; Rafieian-Kopaei, M.; Lorigooini, Z.; Shirzad, H. A screening of anti breast cancer effects and antioxidant activity of twenty medicinal plants gathered from Chaharmahal va Bakhtyari province. *Iran. J. Pharm. Pharmacogn. Res*. **2019**, *7*, 213-222.

Asghari, M.H.; Babaei, E.; Fallah, M.; Mahmoodifar, F. A comparative study on the analgesic properties of five members of Lamiaceae family using two pain models. *Res. J. Pharm*. **2018**, *5*, 31-39.

Băieş, M.-H.; Gherman, C.; Boros, Z.; Olah, D.; Vlase, A.-M.; Cozma-Petruț, A.; Györke, A.; Miere, D.; Vlase, L.; Crișan, G.; et al. The Effects of *Allium sativum* L., *Artemisia absinthium* L., *Cucurbita pepo* L., *Coriandrum sativum* L., *Satureja hortensis* L. and *Calendula officinalis* L. on the Embryogenesis of *Ascaris suum* Eggs during an In Vitro Experimental Study. *Pathogens* **2022**, *11*, 1065.

Băieş, M.-H.; Györke, A.; Cotuţiu, V.-D.; Boros, Z.; Cozma-Petruț, A.; Filip, L.; Vlase, L.; Vlase, A.-M.; Crişan, G.; Spînu, M.; et al. The In Vitro Anticoccidial Activity of Some Herbal Extracts against *Eimeria* spp. Oocysts Isolated from Piglets. *Pathogens* **2023**, *12*, 258.

Bartels, N.; Argyropoulou, A.; Al-Ahmad, A.; Hellwig, E.; Skaltsounis, A.L.; Wittmer, A.; Vach, K.; Karygianni, L. Antibiofilm potential of plant extracts: inhibiting oral microorganisms and Streptococcus mutans. *Front. Dent. Med.* **2025**, *6*, 1535753.

Barzegar, S.; Zare, M.R.; Shojaei, F.; Zareshahrabadi, Z.; Koohi-Hosseinabadi, O.; Saharkhiz, M.J.; Iraji, A.; Zomorodian, Khorram, M. Core-shell chitosan/PVA-based nanofibrous scaffolds loaded with *Satureja mutica* or *Oliveria decumbens* essential oils as enhanced antimicrobial wound dressing. *Int. J. Pharm.* **2021**, *597*, 120288.

Baseri, M.; Naseri, A.; Radmand, F.; Hamishehkar, H.; Memar, M.Y.; Ebrahimi, A.; Asnaashari, S.; Kouhsoltani, M. Effect of nano liposomal herbal extracts against biofilm formation and adherence of *streptococcus mutans*. *Sci. Rep.* **2025**, *15*, 1, 21917.

Basha, E.; Mamoçi, E.; Sharma, A.; Hodaj-Çeliku, E.; Zejnelhoxha, S.; Medeleanu, M.L.; Socaci, S.A.; Bisha, B. Essential Oils from Wild Albanian Lamiaceae: GC-MS Profiling, Biological Activity, and Enhanced Delivery via Nanoencapsulation. *Molecules* **2025**, *30*, 16, 3329.

Bektašević, M.; Carev, I.; Roje, M.; Jurin, M.; Politeo, O. Phytochemical composition and antioxidant activities of the essential oil and extracts of *Satureja subspicata* Vis. growing in Bosnia and Herzegovina. *Chem. Biodivers.* **2017**, *14*, e1700239.

Bimbiraitė-Survilienė, K.; Stankevičius, M.; Šuštauskaitė, S.; Gęgotek, A.; Maruška, A.; Skrzydlewska, E.; Barsteigienė, Z.; Akuņeca, I.; Ragažinskienė, O.; Lukošius, A. Evaluation of chemical composition, radical scavenging and antitumor activities of *Satureja hortensis* L. Herb Extracts. *Antioxidants* **2021**, *10*, 53.

Bona, E.; Cantamessa, S.; Pavan, M.; Novello, G.; Massa, N.; Rocchetti, A.; Berta, G.; Gamalero, E. Sensitivity of *Candida albicans* to essential oils: are they an alternative to antifungal agents?. *J. Appl. Microbiol.* **2016**, *121*, 1530–1545.

Boroja, T.; Katanić, J.; Rosić, G.; Selaković, D.; Joksimović, J.; Mišić, D.; Stanković, V.; Jovičić, N.; Mihailović, V. Summer savory (*Satureja hortensis* L.) extract: Phytochemical profile and

modulation of cisplatin-induced liver, renal and testicular toxicity. *Food. Chem. Toxicol.* **2018**, *118*, 252–263.

Bostanabad, M.A.; Hiradfar, A.; Mohammadpoorasl, A.; Javadzadeh, Y.; Khalvati, B.; Alvandnezhad, T. The effect of mucoadhesive gel containing *Satureja hortensis* extract 1% on severity of chemotherapy-induced mucositis pain in children: a randomized clinical trial. *Int. J. Pediatr*. **2018**, *6*, 7605-7614.

Buyukyildirim, T.; Ocal Ozdamar, F.; Baysal Furtana, G.; Gok, H.N.; Ozek, T.; Orhan, I.E.; Senol Deniz, F.S.; Duman, H. Enzyme inhibitory and antioxidant potential with phytochemical analysis of *Satureja hasturkii* H. Duman & Dirmenci: A new record from Türkiye. *Chem. Biodivers.* **2025**, *22*, 9, e202500328.

Cagal, M.M.; Taner, G.; Kalaycı, S.; Duman, G. Enhanced antibacterial and genoprotective properties of nanoliposomal *Satureja hortensis* L. essential oil. *Drug. Chem. Toxicol*. **2025**, *48*, 1, 180–186

Cagnoli, G.; Bertelloni, F.; Ebani, V.V. In Vitro Antibacterial Activity of Essential Oils from *Origanum vulgare*, *Satureja montana*, *Thymus vulgaris*, and Their Blend Against Necrotoxigenic (NTEC), Enteropathogenic (EPEC), and Shiga-Toxin Producing *Escherichia coli* (STEC) Isolates. *Pathogens* **2024**, *13*, 1077.

Čakar, J.; Lojo, N.K.; Haverić, A.; Hadžić, M.; Lasić, L.; Zeljković, S.Ć.; Haverić, S.; Bajrović, K. *Satureja subspicata* and *S. horvatii* Extracts induce overexpression of the BCl-2 family of anti-apoptotic genes and reduce micronuclei frequency in mice*.* *Nat. Prod. Commun.* **2018**, *13*, 723-726.

Caprioli, G.; Lupidi, G.; Maggi, F. Comparison of chemical composition and antioxidant activities of two Winter savory subspecies (*Satureja montana* subsp. *variegata* and *Satureja montana* subsp. *montana*) cultivated in Northern Italy. *Nat. Prod. Res.* **2019**, *33*, 3143–3147.

Coban, F.; Lan, Y.; Yetisgin, G.; Yuca, H.; Aydın, B.; Angın, H.; Demirci, B.; Karakaya, S. Phytochemical composition and bioactivities of *Satureja montana* L. and *Satureja hortensis* L.: Culinary herbs with antidiabetic, anticholinesterase, and antioxidant potential. *PLoS. One.* **2025**, *20*, *9*, e0332178.

Demyashkin, G.; Tokov, A.; Belokopytov, D.; Shchekin, V.; Borovaya, T.; Lukash, D.; Yuferov, D.; Kulchenko, N.; Tarasov, V.; Blinova, E.; Tsomartova, D.; Shegai, P.; Kaprin, A. Effects of *Satureja montana* L. and *Origanum vulgare* L. Hydrolates in Rabbit Burn Wound Model: Evaluation of Inflammatory, Antioxidant Activity, and Pro-Regenerative Properties in the Skin. *Int. J. Mol. Sci.* **2025**, *26*, 17, 8628.

Dimitrijević, M.; Stojanović-Radić, Z.; Radulović, N.; Nešić, M. Chemical Composition and Antifungal Effect of the Essential Oils of *Thymus vulgaris* L., *Origanum vulgare* L., and *Satureja montana* L. Against Clinical Isolates of *Candida* spp. *Chem. Biodivers.* **2025**, *22*, e202500270.

Dimitrijević, M.V.; Miladinović, L.C.; Marković, M.S.; Arsić, B.; Mihajilov-Krstev, T.M.; Miladinović, D.L. New facts on the antimicrobial Essential oil of *Satureja kitaibelii. Chem. Biodivers.* **2024**, *21*, 2, e202301418.

El Brahimi, R.; El Barnossi, A.; El Moussaoui, A.; Chebaibi, M.; Kachkoul, R.; Baghouz, A.; Nafidi, H.-A.; Salamatullah, A.M.; Bourhia, M.; Bari, A. Phytochemistry and biological activities of essential oils from *Satureja calamintha* Nepeta. *Separations* **2023**, *10*, 344.

Elmdoustazar, P.; Aydın, B.; Önal, M.; Yuca, H.; Karadayı, M.; Gülşahin, Y.; Demirci, B.; Karakaya, S.; Güvenalp, Z. Phytochemical Traits and Biological Activity of *Satureja hortensis* and *Satureja macrantha* as Culinary Spices Using GC–MS/MS and LC–MS/MS Techniques. *Food Sci. Nutr.* **2025**, *13*, e70733.

Emre, İ.; Kurşat, M.; Yilmaz, Ö.; Erecevit, P. Chemical compositions, radical scavenging capacities and antimicrobial activities in seeds of *Satureja hortensis* L. and *Mentha spicata* L. subsp. *spicata* from Turkey. *Braz. J. Biol.* **2021**, *81*, 144–153.

Esmaeilbeig, M.; Kouhpayeh, S.A.; Amirghofran, Z. An Investigation of the growth inhibitory capacity of several medicinal plants from Iran on tumor cell lines. *Iran. J. Cancer. Prev*. **2015**, *8*, e4032.

Esmaeili-Mahani, S.; Ebrahimi, B.; Abbasnejad, M.; Rasoulian, B.; Sheibani, V. *Satureja khuzestanica* prevents the development of morphine analgesic tolerance through suppression of spinal glial cell activation in rats. *J. Nat. Med*. **2015**, *69*, 165-170.

Farzaneh, Z.; Kalantar, K.; Iraji, A.; Amirghofran, Z. Inhibition of LPS-induced inflammatory responses by *Satureja hortensis* extracts in J774.1 macrophages. *J. Immunoass. Immunochem.* **2018**, *39*, 274–291.

Fitsiou, E.; Anestopoulos, I.; Chlichlia, K.; Galanis, A.; Kourkoutas, I.; Panayiotidis, M.I.; Pappa, A. Antioxidant and antiproliferative properties of the essential oils of *Satureja thymbra* and *Satureja parnassica* and their major constituents. *Anticancer. Res.* **2016**, *36*, 5757–5763.

Fratini, F.; Pecorini, C.; Resci, I.; Copelotti, E.; Nocera, F.P.; Najar, B.; Mancini, S. Evaluation of the Synergistic Antimicrobial Activity of Essential Oils and Cecropin A Natural Peptide on Gram-Negative Bacteria. *Animals* **2025**, *15*, 2, 282.

Gopčević, K.; Grujić, S.; Arsenijević, J.; Džamić, A.; Veličković, I.; Izrael-Živković, L.; Medić, A.; Mudrić, J.; Soković, M.; Đurić, A. Bioactivity and phenolics profile of aqueous and ethyl acetate extracts of *Satureja kitaibelii* Wierzb. ex Heuff. obtained by ultrasound-assisted extraction. *Sci. Rep.* **2022**, *12*, 21221.

Gopčević, K.; Grujić, S.; Arsenijević, J.; Karadžić, I.; Izrael-Živković, L.; Maksimović, Z. Phytochemical properties of *Satureja kitaibelii*, potential natural antioxidants: a new insight. *Plant. Food. Hum. Nutr.* **2019**, *74*, 179–184.

Harmati, M.; Gyukity-Sebestyen, E.; Dobra, G.; Terhes, G.; Urban, E.; Decsi, G.; Mimica-Dukić, N.; Lesjak, M.; Simin, N.; Pap, B.; Nemeth, I.B.; Buzas, K. Binary mixture of *Satureja hortensis* and *Origanum vulgare* subsp. *hirtum* essential oils: in vivo therapeutic efficiency against *Helicobacter pylori* infection. *Helicobacter* **2017**, *22*, e12350.

Hasheminya, S.M.; Mokarram, R.R.; Ghanbarzadeh, B.; Hamishekar, H.; Kafil, H.S.; Dehghannya, J. Development and characterization of biocomposite films made from kefiran, carboxymethyl cellulose and *Satureja Khuzestanica* essential oil. *Food. Chem.* **2019**, *289*, 443–452.

Hassanabadi, N.; Meymand, Z.M.; Ashrafzadeh, A.; Sharififar, F. Antioxidant and cytotoxicity activity of a nanoemulsion from *Satureja kermanica* (Lamiaceae). *Ann. Pharm. Fr.* **2024**, *82*, 4, 645-653.

Hickl, J.; Argyropoulou, A.; Al-Ahmad, A.; Hellwig, E.; Skaltsounis, A.L.; Wittmer, A.; Vach, K.; Karygianni, L. Unleashing nature's defense: potent antimicrobial power of plant extracts against oral pathogens and *Streptococcus mutans* biofilms. *Front. Oral. Health.* **2024**, *5*, 1469174.

Huwaimel, B.; Abouzied, A.S.; Anwar, S.; Elaasser, M.M.; Almahmoud, S.A.; Alshammari. B.; Alrdaian, D.; Alshammari. R.Q. Novel landmarks on the journey from natural products to pharmaceutical formulations: Phytochemical, biological, toxicological and computational activities of *Satureja hortensis* L. *Food Chem. Toxicol*. **2023**, *179*, 113969.

Ilhan, E.; Cesur, S.; Guler, E.; Topal, F.; Albayrak, D.; Guncu, M.M.; Cam, M.E.; Taskin, T.; Sasmazel H.T.; Aksu, B.; Oktar, F.N.; Gunduz, O. Development of *Satureja cuneifolia*-loaded sodium alginate/polyethylene glycol scaffolds produced by 3D-printing technology as a diabetic wound dressing material. *Int. J. Biol. Macromol.* **2020**, *161*, 1040–1054.

Jafari, F.; Farmani, F.; Zomorodian, K.; Moein, M.; Faridi, P.; Zarshenas, M. M. A study on essential oil chemical compositions, antioxidant, and antimicrobial activities of native and endemic *Satureja* species growing in Iran. *Pharm. Chem. J.* **2018**, *52*, 63-68.

Jafri, S.A.A.; Khalid, Z.M.; Khan, M.R.; Ashraf, S.; Ahmad, N.; Karami, A.M.; Rafique, E.; Ouladsmane, M.; Al Suliman, N.M.S.; Aslam, S. Evaluation of some essential traditional medicinal plants for their potential free scavenging and antioxidant properties. *J. King. Saud. Univ. Sci.* **2023**, *35*, 102562.

Jahanshahi, S.; Kheirandish, F.; Kazemi, B.; Montazeri, M.; Fallahi, S.; Rouzbahani, A.K.; Mamaghani, A.J. Investigating the Effect of *Satureja khuzestanica* Essential oil on MDR1 Gene Expression in Leishmania major. *Acta. Parasitol.* **2024**, *69*, 526-532.

Jakupović, L.; Strawa, J.W.; Nižić Nodilo, L.; Marijan, M.; Hafner, A.; Jakimiuk, K.; Tomczykowa, M.; Tomczyk, M.; Končić, M.Z. Cosmeceutical and Wound-Healing Activities of Green Hydroxypropyl-*β*-Cyclodextrin-Glycerol-Based *Satureja montana* Extracts. *Molecules* **2025**, *30*, 2638.

Jaradat, N.; Hawash, M.; Al-Maharik, N.; Qadi, M. Phytochemical Profiling and Bioactive Properties of Essential Oils from Endemic Palestinian *Satureja thymbrifolia*. *Chem. Biodivers.* **2025**, *22*, e202402136.

Jovanova, B.; Panovska, T.K. Evaluation of the antioxidant effects and cytotoxic potential of selected herbs used in traditional medicine. *J. Anim. Plant Sci*. **2019**, *29*, 1466-1475.

Khalil, N.; El-Jalel, L.; Yousif, M.; Gonaid, M. Altitude impact on the chemical profile and biological activities of *Satureja thymbra* L. essential oil. *BMC complement. Med. Ther.* **2020**, *20*, 1, 186.

Khani, S.; Seyedjavadi, S.S.; Zare-Zardini, H.; Hosseini, H.M.; Goudarzi, M.; Khatami, S.; Amani, J.; Imani Fooladi, A.A.; Razzaghi-Abyaneh, M. Isolation and functional characterization of an antifungal hydrophilic peptide, Skh-AMP1, derived from *Satureja khuzistanica* leaves. *Phytochemistry* **2019**, *164*, 136–143.

Kheiri, E.; Bonab, Z.H.; Soltanzadeh, H. Investigating the Expression of Apoptosis and Metastasis Genes in *Satureja khuzistanica* along with Nisin and Doxorubicin in Colorectal Cancer. *Pharmacog. Res.* **2025**, *17*, 1197-204.

Kim, J.E.; Lee, J.E.; Huh, M.J.; Lee, S.C.; Seo, S.M.; Kwon, J.H.; Park, I.K. Fumigant antifungal activity via reactive oxygen species of *Thymus vulgaris* and *Satureja hortensis* essential oils and constituents against *Raffaelea quercus-mongolicae* and *Rhizoctonia solani*. *Biomolecules* **2019**, *9*, 561.

Kınoğlu, B.K.; Gülçin, İ.; Gören, A.C. Quantification of secondary metabolites of *Satureja pilosa* (Lamiaceae) by LC-HRMS and evaluation of antioxidant and cholinergic activities, *Rec. Nat. Prod.* **2024**, *18*, 6, 674-686.

Kremer, D.; Kosir, I.J.; Koncic, M.Z.; Cerenak, A.; Potocnik, T.,; Srecec S.; Kosalec, I. Antimicrobial and antioxidant properties of *Satureja Montana* L. and *S. Subspicata* Vis. (Lamiaceae). *Curr. Drug. Targets* **2015**, *16*, 1623–1633.

Krimat, S.; Dahmane, D.; Senani, R.; Merah, S.; Ksouri, A.; Tigrine, C.; Benyammi, R.; Alili, M.; Metidji, H.; Nouasri, A.; Dob, T. Essential oil of Algerian endemic *Satureja candidissima* (Munby): A study of its biological activities. *J. Essent. Oil-Bear. Plants.* **2025**, *28*, 1357-1368.

Kulić, M.; Drakul, D.; Sokolović, D.; Kordić-Bojinović, J.; Milovanović, S.; Blagojević, D. Essential oil of *Satureja montana* L. from Herzegovina: Assessment of composition, antispasmodic, and antidiarrheal effects. *Rec. Nat. Prod*. **2023**, *17*, 536-548.

Les, F.; Galiffa, V.; Cásedas, G.; Moliner, C.; Maggi, F.; López, V.; Gómez-Rincón, C. Essential Oils of Two Subspecies of *Satureja montana* L. against Gastrointestinal Parasite *Anisakis simplex* and Acetylcholinesterase Inhibition. *Molecules* **2024**, *29*, 19, 4640.

Lesjak, M.; Simin, N.; Orcic, D.; Franciskovic, M.; Knezevic, P.; Beara, I.; Aleksic, V.; Svircev, E.; Buzas, K.; Mimica-Dukic, N. Binary and tertiary mixtures of *Satureja hortensis* and *Origanum vulgare* essential oils as potent antimicrobial agents against *Helicobacter pylori*. *Phytother. Res.* **2016**, *30*, 476–484.

Mafakheri, H.; Mirghazanfari, S.M. Antifungal activity of the essential oils of some medicinal plants against human and plant fungal pathogens. *Cell Mol. Biol*. **2018**, *64*, 13-19.

Mahboubi, M.; Kazempour, N. The antibacterial activity of *Satureja khuzestanica* essential oil against clinical isolates of *E. coli*. *J. Nat. Pharm. Prod.* **2016**, *11*, e30034.

Mahmoudvand, H.; Badparva, E.; Baharvand, Z.; Lalehmarzi, H.S. Anti-Trichomonas vaginalis activities and apoptotic effects of some Iranian medicinal plants. *Trop. Biomed*. **2018**, *35*, 347-353.

Maktabi, S.; Rashnavadi, R.; Tabandeh, M.R.; Sourestani, M.M. Effective Inhibition of *Listeria monocytogenes* Biofilm Formation by *Satureja rechingeri* Essential Oil: Mechanisms and Implications. *Curr. Microbiol.* **2024**, *81*, 77.

Mandalakis, M.; Anastasiou, T.I.; Martou, N.; Keisaris, S.; Greveniotis, V.; Katharios, P.; Lazari, D.; Krigas, N.; Antonopoulou, E. Antibacterial effects of essential oils of seven medicinal-aromatic plants against the fish pathogen *Aeromonas veronii* bv. sobria: to blend or not to blend?. *Molecules* **2021**, *26*, 2731.

Maral, H.; Oğuz, M.; Türkmen, M.; Soylu, S. Chemical profile and bioactivity of essential oils from five Turkish thyme species against white mold fungal disease agent *Sclerotinia sclerotiorum*. *Sci. Rep.* **2025***, 15*, 35699.

Maravić-Vlahoviček, G.; Kindl, M.; Andričević, K.; Obranić, S.; Vladimir-Knežević, S. Modulatory Effects of *Satureja montana* L. Essential Oil on Biofilm Formation and Virulence Factors of *Pseudomonas aeruginosa*. *Pharmaceuticals* **2025**, *18*, 1269.

Mašković, J. M.; Jakovljević, V.; Živković, V.; Mitić, M.; Kurćubić, L. V.; Mitić, J.; Mašković, P. Z. Optimization of Ultrasound-Assisted Extraction of Phenolics from *Satureja hortensis* L. and Antioxidant Activity: Response Surface Methodology Approach. *Processes* **2024**, *12*, 2042.

Mašković, P.; Veličković, V.; Mitić, M.; Đurović, S.; Zeković, Z.; Radojković, M.; Cvetanović, A.; Švarc-Gajić, J.; Vujić, J. Summer savory extracts prepared by novel extraction methods resulted in enhanced biological activity. *Ind. Crop. Prod.* **2017**, *109*, 875-881.

Masoum, S.; Samadi, N.; Mehrara, B.; Mahboubi, M. otentiality of independent component regression in assessment of the peaks responsible for antimicrobial activity of *Satureja hortensis* L. and *Oliveria decumbens* Vent. using GC–MS. *J. Iran. Chem. Soc*. **2018**, *15*, 2007-2016.

Memarzadeh, S. M.; Gholami, A.; Pirbalouti, A. G.; Masoum, S. Bakhtiari savory (*Satureja bachtiarica* Bunge.) essential oil and its chemical profile, antioxidant activities, and leaf micromorphology under green and conventional extraction techniques. *Ind. Crop. Prod.* **2020**, *154*, 112719.

Miguel, M.G.; da Silva, C.I.; Farah, L.; Castro Braga, F.; Figueiredo, A.C. Effect of essential oils on the release of TNF-α and CCL2 by LPS-stimulated THP-1 Cells. *Plants* **2020**, *10*, 50.

Mirderikvandi, M.; Khosravinia, H.; Parizadian Kavan, B. Independent and combined effects of *Satureja khuzistanica* essential oils and acetic acid on prevalence and intensity of fatty liver syndrome in broiler chickens. *J. Anim. Physiol. An. N.* **2020**, *104*, 166–177.

Mohammadi-Ziveh, Z.; Mirhosseini, S.A.; Hosseini, H.M. *Satureja Khuzestanica* mediated synthesis of silver nanoparticles and its evaluation of antineoplastic activity to combat colorectal cancer cell line. *Iran. J. Pharm. Res*. **2020**, *19*, 169–180.

Moreira, S.A.; Pintado, M.E.; Saraiva, J.A. Optimization of antioxidant activity and bioactive compounds extraction of winter savory leaves by high hydrostatic pressure. *High Press. Res*. **2020**, *40*, 543-560.

Mosaddegh, M., Irani, M. Inhibition test of heme detoxification (ITHD) as an approach for detecting antimalarial agents in medicinal plants. *Res. J. Pharmacogn*. **2018**, *5*, 5-11.

Muthukumar, A.; Mittal, S.; Choezom, T.; Bhavani, K.; Das, K.; Joyce, N.; Almuqbil, M.; Almadani, M.E.; Ahmad, F.; Yasmin, F. Evaluation of the cardioprotective activity of summer savory (*Satureja hortensis* L.) extract in experimental rats with Isoproterenol-induced myocardial infarction. *J. King Saud Univ. Sci.* **2024**, *36*, 103236.

Narchin, F.; Larijani, K.; Rustaiyan, A.; Ebrahimi, S.N.; Tafvizi, F. Phytochemical synthesis of silver nanoparticles by two techniques using *Saturaja rechengri* Jamzad extract: identifying and comparing *in Vitro* anti-proliferative activities. *Adv. Pharmaceut. Bull.* **2018**, *8*, 235–244.

Nasimi, P., Vahdati, A.; Tabandeh, M.R.; Khatamsaz, S. Cytoprotective and anti-apoptotic effects of *Satureja khuzestanica* essential oil against busulfan-mediated sperm damage and seminiferous tubules destruction in adult male mice. *Andrologia* **2016**, *48*, 74–81.

Nikolić, M.; Jovanović, K.K.; Marković, T.; Marković, D.; Gligorijević, N.; Radulović, S.; Soković, M. Chemical composition, antimicrobial, and cytotoxic properties of five Lamiaceae essential oils. *Ind. Crops. Prod.* **2014**, *61*, 225-232.

Obeidnejad, E.; Kavoosi, G.; Saharkhiz, M.J. Antioxidant, anti-amylase, anti-lipase, and efficiency of *Satureja* fatty acid on the anti-inflammatory parameters in lipopolysaccharide-stimulated macrophage through Nrf2/NF-kB/NADH oxidase pathway. *Sci. Rep.* **2024**, *14*, 12490.

Oliveira-Pinto, P.R.; Oliveira-Fernandes, J.; Mariz-Ponte, N.; Monge-Mora, P.; Guido, L.F.; Fernandes-Ferreira, M.; Sousa, R.M.O.F.; Santos, C. *Satureja montana* L. essential oil and montmorillonite nanoclay modulate the phenylpropanoid pathway and polyphenols biosynthesis of tomato plants suffering from bacterial spot disease. *Planta* **2025**, *262*, 121.

Pavlović, O.M.; Kolarević, S.; Đorđević, J.; Jovanović Marić, J.; Lunić, T.; Mandić, M.; Kračun Kolarević, M.; Živković, J.; Alimpić Aradski, A.; Marin, P.D.; Šavikin, K. A study of phytochemistry, genoprotective activity, and antitumor effects of extracts of the selected Lamiaceae species. *Plants* **2021**, *10*, 2306.

Payandeh, M.; Ahmadyousefi, M.; Alizadeh, H.; Zahedifar, M. Chitosan nanocomposite incorporated *Satureja kermanica* essential oil and extract: synthesis, characterization and antifungal assay. *Int. J. Biol. Macromol.* **2022**, *221*, 1356–1364.

Pereira, G.; Faria, J.M.S. Activity of *Satureja montana* Allelochemical Volatiles Against the Pinewood Nematode. *Chem. Proc.* **2024**, *16*, 8.

Raadani, A.; Boulila, A.; Yangui, I.; Boussaid, M.; Messaoud, C.; Ben Elhadj Ali, I. Variation in Phenolic Content, Antioxidant Activity and Alpha-amylase and Acetylcholinesterase Inhibitory Capacities of Different Extracts from Tunisian *Satureja barceloi* (Willk) L. *Chem. Biodivers*. **2024**, *21*, e202302109.

Rabiei, Z.; Shirchi, M.; Rafieian-Kopaei, M.; Asgharzade, S. Effects of *Satureja bachtiarica* essential oil in preventing seizure in pentylenetetrazol-kindled mice. *Basic Clin. Neurosci*. **2022**, *13*, 465–475.

Rahimmalek, M.; Afshari, M.; Sarfaraz, D.; Miroliaei, M. Using HPLC and multivariate analyses to investigate variations in the polyphenolic compounds as well as antioxidant and antiglycative activities of some Lamiaceae species native to Iran. *Ind. Crops. Prod.* **2020**, *154*, 112640.

Raikwar, G.; Mohan, S.; Dahiya, P. Combined antibacterial effect of essential oils from three Indian medicinal plants and antibiotic tetracycline on MRSA using simplex centroid mixture design. *Sci. Rep.* **2025**, *15*, 34816.

Ramezani, M.; Ehtesham-Gharaee, M.; Khazaie, M.; Behravan, J. *Satureja hortensis* L. methanolic extract and essential oil exhibit antitumor activity. *J. Essent. Oil Bear. Pl*. **2016**, *19*, 148-154.

Ratajac, R.; Pavlićević, A.; Petrović, J.; Stojanov, I.; Orčić, D.; Štrbac, F.; Simin, N. In vitro evaluation of acaricidal efficacy of selected essential oils against *Dermanyssus gallinae*. *Pak. Vet. J.* **2024**, *44*, 93-98.

Ratajac, R.; Štrbac, F.; Petrović, J.; Stojanov, I.; Pušić, I.; Kačarević, T.; Simin, N.; Orčić, D.; Stojanović, D.; Hailan, W.A.; Mares, M.M. Evaluation of antibacterial potential of *Satureja montana* L., *Ocimum basilicum* L. and *Salvia officinalis* L. essential oils against reproductive tract pathogens in cattle and their toxicity impact on endometrial and kidney cells. *Pak. Vet. J.* **2025**, *45*, 1122-1134.

Rezaei, A.; Monfared-Hajishirkiaee, R.; Hosseinzadeh-Moghaddam, S.; Behzadi, M.; Shahangian, S.S. Enhancing leachate management with antibacterial nanocomposites incorporating plant-based carbon dots and *Satureja Khuzestanica* essential oils. *Colloids. Surf. B.* **2025**, *245*, 114296.

Rezende, D.; Oliveira, C.D.; Batista, L.R.; Ferreira, V.R.; Brandão, R.M.; Caetano, A.R.; Alves, M.V.; Cardoso, M.G. Bactericidal and antioxidant effects of essential oils from *Satureja montana* L., *Myristica fragrans* H. and *Cymbopogon flexuosus*. *Lett. Appl. Microbiol.* **2022**, *74*, 741–751.

Roosta, S.; Ghasemi, F.; Mokhayeri, Y.; Choobkar, S.; Nikbakht, M.R.; Falahi, E. Effects of *Satureja Khuzestanica* supplementation on glycemic indices and lipid profile in type 2 diabetes patients: a randomized controlled clinical-trial. *BMC Complement. Med. Ther.* **2024**, *24*, 201.

Saboori, K.; Nassira, M.; Safari, M.; Namdar, N.; Montaseri, Z.; Osanloo, M. Antibacterial activity of a conventional hydrogel and a nanoparticle based hydrogel containing *Satureja khuzestanica* essential oil. *Sci. Rep.* **2025**, *15*, 1, 21267.

Saidi, A.E.; Bouzidi, N.; Ziane, M.; Gherib, M.; Rahila, C.; Mioc, M. In silico and in vitro studies: investigating the chemical composition, DFT, molecular docking, and dynamic simulation of *Satureja candidissima* (Munby) Briq essential oil as a potential antibacterial agent. *J. Biomol. Struct. Dyn.* **2025**, *43*, 4043–4062.

Samani, R. M.; D'Urso, G.; Nazzaro, F.; Fratianni, F.; Masullo, M.; Piacente, S. Phytochemical Investigation and Biofilm-Inhibitory Activity of Bachtiari Savory (*Satureja bachtiarica* Bunge) Aerial Parts. *Plants* **2024**, *13*, 67.

Sánchez-Quintero, M.J.; Delgado, J.; Medina-Vera, D.; Becerra-Muñoz, V.M.; Queipo-Ortuño, M.I.; Estévez, M.; Plaza-Andrades, I.; Rodríguez-Capitán, J.; Sánchez, P.L.; Crespo-Leiro, M.G.; Jiménez-Navarro, M.F. Beneficial effects of essential oils from the Mediterranean diet on gut microbiota and their metabolites in ischemic heart disease and type-2 diabetes mellitus. *Nutrients* **2022**, *14*, 4650.

Sasanian, N.; Sari, A.A.; Mortazavian, A.M. Effects of *Thymus daenensis* and *Satureja hortensis* L. essential oils on quality properties of Iranian Doogh. *J. Food. Saf.* **2018**, *38*, e12527.

Shanaida, M. Antioxidant activity of essential oils obtained from aerial part of some Lamiaceae species. *Int. J. Green Pharm*. **2018**, *12*, 200-204.

Sharifi, A.; Mohammadzadeh, A.; Zahraei Salehi, T.; Mahmoodi, P. Antibacterial, antibiofilm and antiquorum sensing effects of *Thymus daenensis* and *Satureja hortensis* essential oils against *Staphylococcus aureus* isolates. *J. Appl. Microbiol.* **2018**, *124*, 379–388.

Sharifi-Rad, J.; Sharifi-Rad, M.; Hoseini-Alfatemi, S.M.; Iriti, M.; Sharifi-Rad, M.; Sharifi-Rad, M. Composition, cytotoxic and antimicrobial activities of *Satureja intermedia* C.A. mey essential Oil. *J. Mol. Sci.* **2015**, *16*, 17812–17825.

Šimunović, K.; Bucar, F.; Klančnik, A.; Pompei, F.; Paparella, A.; Smole Možina, S. In vitro effect of the common culinary herb winter savory (*Satureja montana*) against the infamous food pathogen *Campylobacter jejuni*. *Foods* **2020**, *9*, 537.

Soodi, M.; Saeidnia, S.; Sharifzadeh, M.; Hajimehdipoor, H.; Dashti, A.; Sepand, M.R.; Moradi, S. *Satureja bachtiarica* ameliorate beta-amyloid induced memory impairment, oxidative stress and cholinergic deficit in animal model of Alzheimer's disease. *Metab. Brain. Dis*. **2016***, 31*, 395–404.

Štrbac, F.; Bosco, A.; Maurelli, M.P.; Ratajac, R.; Stojanović, D.; Simin, N.; Orčić, D.; Pušić, I.; Krnjajić, S.; Sotiraki, S.; Saralli, G. Anthelmintic properties of essential oils to control gastrointestinal nematodes in sheep—In vitro and in vivo studies. *Vet. Sci*. **2022**, *9*, 93.

Štrbac, F.; Krnjajić, S.; Ratajac, R.; Rinaldi, L.; Musella, V.; Castagna, F.; Stojanović, D.; Simin, N.; Orčić, D.; Bosco, A. Anthelmintic activity of winter savory (*Satureja montana* L.) essential oil against gastrointestinal nematodes of sheep. *BMC Vet. Res.* **2025**, *21*, 405.

Teofilović, B.; Gligorić, E.; Ninić, M.; Vukmirović, S.; Gagić, Ž.; Mandić-Kovačević, N.; Tubić, B.; Đukanović, Đ.; Grujić-Letić, N. Green Extraction Combined with Chemometric Approach: Profiling Phytochemicals and Antioxidant Properties of Ten Species of the Lamiaceae Family. *Separations* **2025**, *12*, 155.

Toplu, Y.; Önlü, H. Anti-Listerial Effects of *Satureja hortensis* Essential Oils in Ready-to-Eat Poultry Meat Stored at Different Temperatures. *Microbiol. Res.* **2025**, *16*, 195.

Valentini, F.; Colasanti, I.A.; Zaratti, C.; Filimon, D.; Macchia, A.; Neri, A.; Relucenti, M.; Reverberi, M.; Allegrini, I.; Guerriero, E.; et al. TiO_2 and $CaCO_3$ Microparticles Produced in Aqueous Extracts from *Satureja montana:* Synthesis, Characterization, and Preliminary Antimicrobial Test. *Molecules* **2025**, *30*, 4138.

Vanti, G.; Tomou, E.M.; Stojković, D.; Ćirić, A.; Bilia, A.R.; Skaltsa, H. Nanovesicles loaded with *Origanum onites* and *Satureja thymbra* essential oils and their activity against food-borne pathogens and spoilage microorganisms. *Molecules* **2021**, *26*, 2124.

Vilmosh, N.; Georgieva-Kotetarova, M.; Kandilarov, I.; Zlatanova-Tenisheva, H.; Murdjeva, M.; Kirina, V.; Dimitrova, S.; Katsarova, M.; Denev, P.; Kostadinova, I. Anti-inflammatory and in vitro antioxidant activities of *Satureja montana* dry extract. *Fol. Med.* **2024**, *66*, 1, 114-122.

Vitanza, L.; Maccelli, A.; Marazzato, M.; Scazzocchio, F.; Comanducci, A.; Fornarini, S.; Crestoni, M.E.; Filippi, A.; Fraschetti, C.; Rinaldi, F.; Aleandri, M. *Satureja montana* L. essential oil and its antimicrobial activity alone or in combination with gentamicin. *Microb. Pathog.* **2019**, 126, 323–331.

Vladić, J.; Ćebović, T.; Vidović, S.; Jokić, S. Evaluation of anticancer activity of *Satureja montana* supercritical and spray-dried extracts on Ehrlich's ascites carcinoma bearing mice. *Plants*, **2020**, *9*, 11, 1532.

Yıldız, A.N.; Çarıkçı, S.; Dirmenci, T.; Kartal, M.; Gülcin, ˙ I.; Gören, A.C. Secondary Metabolite Profiling of *Satureja aintabensis* P.H. Davis and *Satureja spicigera* (K. Koch) Boiss. by LC-HRMS and Evaluation of Antioxidant and Anticholinergic Activities. *Life* **2025**, *15*, 1272.

Yıldız, G.; İlgün, S.; Şeker Karatoprak, G.; Köse, Y. B.; Göger, F.; Temel, H.E.; Demirci, B. Chemical profile, *in vitro* pharmacological activity and *Satureja cuneifolia* Ten. evaluation of essential oil based on distillation time. *Int. J. Environ. Health. Res.* **2023**, *20*, 1–17.

Yuan, X.; Cao, D.; Xiang, Y.; Jiang, X.; Liu, J.; Bi, K.; Dong, X.; Wu, T.; Zhang, Y. Antifungal activity of essential oils and their potential synergistic effect with amphotericin B. *Sci. Rep.* **2024**, *14*, 31125.

Yuan, Y.; Hui, X.; Liu, Z.; Sun, J.; Raka, R.N.; Xiao, J.; Zhang, Z.; Wu, H. Investigation of differential Multi-Mode antibacterial mechanisms of essential oils of *Satureja montana* L. and *Leptospermum scoparium* JR Forst. & G. Forst. Against *Porphyromonas gingivalis*. *BMC Complement. Med. Ther.* **2025**, *25*, 283.

Zeidán-Chuliá, F.; Keskin, M.; Könönen, E.; Uitto, V.J.; Söderling, E.; Moreira, J.C.; Gürsoy, U.K. Antibacterial and antigelatinolytic effects of *Satureja hortensis* L. essential oil on epithelial cells exposed to *Fusobacterium nucleatum*. *J. Med. Food*. **2015**, *18*, 503–506.

Zomorodian, K.; Ghadiri, P.; Saharkhiz, M.J.; Moein, M.R.; Mehriar, P.; Bahrani, F.; Golzar, T.; Pakshir, K.; Fani M.M. Antimicrobial activity of seven essential oils from Iranian aromatic plants against common causes of oral infections. *Jundishapur J. Microbiol*. **2015**, *8*, e17766.
